# First series of *N*-alkylamino peptoid homooligomers: solution phase synthesis and conformational investigation

**DOI:** 10.3762/bjoc.18.85

**Published:** 2022-07-14

**Authors:** Maxime Pypec, Laurent Jouffret, Claude Taillefumier, Olivier Roy

**Affiliations:** 1 Université Clermont Auvergne, Clermont Auvergne INP, CNRS, ICCF, F-63000 Clermont–Ferrand, France

**Keywords:** *cis*/*trans* isomerism, peptoid, structure, *trans*-inducing side chain

## Abstract

The synthesis and conformational analysis of the first series of peptoid oligomers composed of consecutive *N*-(alkylamino)glycine units is investigated. We demonstrate that *N*-(methylamino)glycine homooligomers can be readily synthesized in solution using *N*-Boc-*N*-methylhydrazine as a peptoid submonomer and stepwise or segment coupling methodologies. Their structures were analyzed in solution by 1D and 2D NMR, in the solid state by X-ray crystallography (dimer **2**), and implicit solvent QM geometry optimizations. *N*-(Methylamino)peptoids were found to preferentially adopt *trans* amide bonds with the side chain N–H bonds oriented approximately perpendicular to the amide plane. This orientation is conducive to local backbone stabilization through intra-residue hydrogen bonds but also to intermolecular associations. The high capacity of *N*-(methylamino)peptoids to establish intermolecular hydrogen bonds was notably deduced from pronounced concentration-dependent N–H chemical shift variation in ^1^H NMR and the antiparallel arrangement of mirror image molecules held together via two hydrogen bonds in the crystal lattice of dimer **2**.

## Introduction

**Figure 1:**
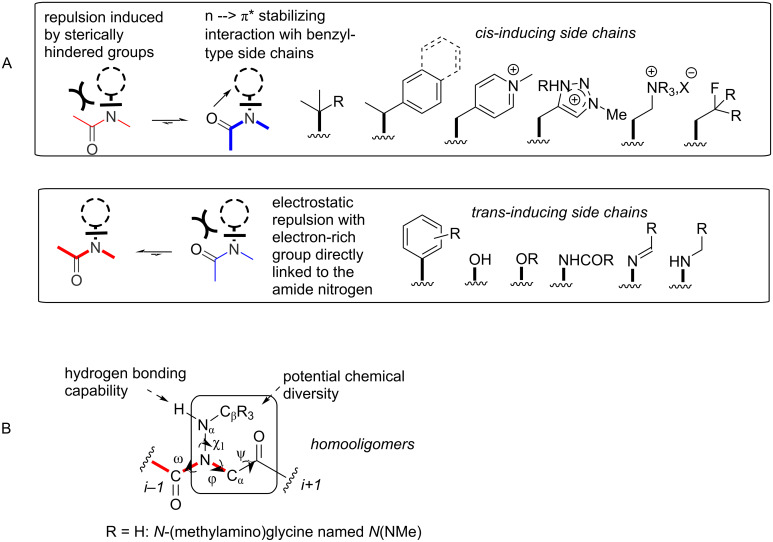
(A) Summary of the main side chains exerting significant steric and/or electronic effects and influencing the amide conformation of peptoids. (B) Atom labels in *N*-(methylamino)glycine monomers.

The term “peptoids” refers to the family of artificial oligo(poly)mers consisting of *N*-substituted glycines [1–2]. They retain the same backbone as peptides except that the side chains are located on the nitrogen atoms of the amide bonds and thus represent an important class of peptide biomimetics [3–5], generally with improved cell permeability [6] and proteolytic resistance [7–8]. Beyond their resemblance to peptides, the obvious interest in this family of peptidomimetics arises from their ease of synthesis by the modular submonomer protocol [9] which enables the incorporation of numerous primary amine synthons in a sequence-controlled manner [10], and application of solid-supported combinatorial approaches [11–13]. The most relevant comparison of peptoids with peptides is in fact with polyprolines due to the presence of backbone tertiary amide linkages, much more prone to *cis*/*trans* equilibria than secondary amides. Indeed, in proteins, *cis*-amide bonds are most often observed for Xaa–Pro amide bonds and polyproline chains can adopt either the all-*trans* type II (PPII) or the all-*cis* type I (PPI) helical conformations, the latter being only observed in alcohol-type solvents [14]. In contrast to the prolyl-amide bond in acyclic peptides (≈5% of *cis*-Pro) [15], the *cis* conformation of peptoid amide bonds is generally much more populated, leading to substantial conformational heterogeneity [16]. Thus, adoption of well-defined secondary structures requires fine control of backbone amide isomerism. Considerable efforts have been made to regulate the conformation of peptoids through steric and electronic interactions involving peptoid amides and nearby side chains [17–18]. For example, *N*-substituted monomers bearing benzylic-type *N*α-chiral groups including the phenylethyl [19–21], naphthylethyl [17,22–24], and triazolium groups [25–27], alkyl ammonium [28], *tert*-butyl/α,α-*gem*-dimethyl [29], or fluorinated groups [30] will preferentially form *cis*-amides (Figure 1A). Peptoid helicity modulation has also been investigated through specific placement of chiral and achiral monomers [31–32]. Comparatively fewer *N*-functional monomers capable of promoting *trans*-peptoid amides were designed. Among these are the *N*-aryl [33–35], *N*-hydroxy [36], *N*-alkoxy [37], and *N*-(acylhydrazide)glycines (Figure 1A) [38–39]. Recently, while our work was in progress, *N*-imino and *N-*(alkylamino)glycines have also been proposed to build up peptoids with *trans* amide bonds [40]. In this seminal publication, hydrazones were utilized as submonomers in the displacement step of resin-bound bromoacetylated peptoids and cleavage from the resin with TFA containing 5% of triethylsilane resulted in a concomitant reduction of the imine functions in *N*-alkylamino groups. In this work, however, the *N*-alkylamino-containing glycine units were not introduced consecutively but every two or three residues. We describe here the synthesis and study of the first representatives of peptoids containing exclusively *N*-alkylamino-substituted amides. As the first representatives of this family we chose to synthesize peptoid oligomers containing *N*-substituted methylamino amides, considering that the methods developed could be used for the synthesis of other members of this family (Figure 1B).

## Results and Discussion

### Synthesis

**Figure 2:**
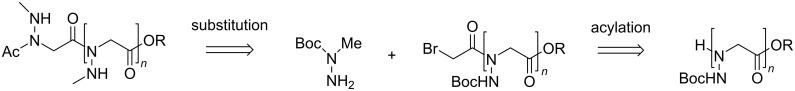
Solution-phase synthesis of *N*-(methylamino)glycine oligomers using *N*-Boc-*N*-methylhydrazine as a submonomer.

**Scheme 1:**
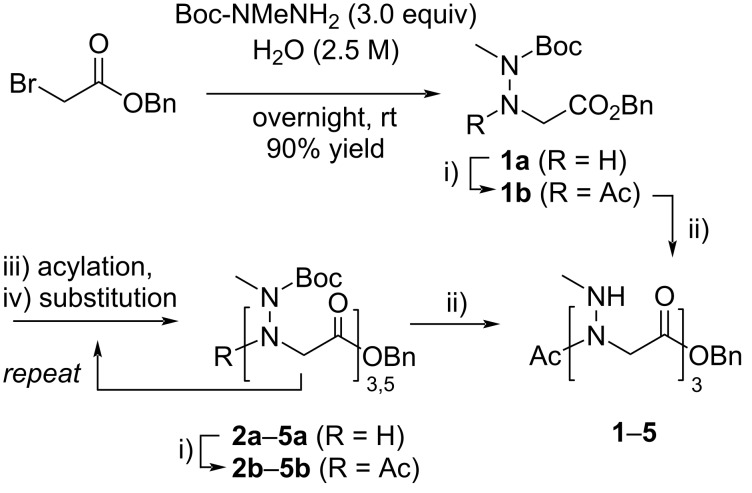
Submonomer synthesis used for the construction of peptoids **1**–**5** containing *N*-methylamino side chains. Conditions: i) Ac_2_O (8.0 equiv), Et_3_N (4.0 equiv), EtOAc (0.2 M), rt, 48 h; ii) TFA/CH_2_Cl_2_ 1:1, 0 °C to rt, 30 min; iii) BrCH_2_COBr (1.5 equiv), Et_3_N (2.0 equiv), THF (0.2 M), −10 °C, from 30 min to 2 h; iv) *N*-Boc-*N*-methylhydrazine (3.0 equiv), H_2_O/MeOH 1:1 (1.25 M), 60 °C, overnight.

A solution-phase approach using commercially available *N*-Boc-*N*-methylhydrazine as a submonomer was adopted in this work (Figure 2). Benzyl bromoacetate, rather than *tert-*butyl bromoacetate, successfully used in the past for the synthesis of peptoids in solution [22], was chosen as the starting substrate to ensure orthogonality of the *C*-terminal protecting group with respect to the Boc side chain protections. For the submonomer solution-phase synthesis of monomer **1** and oligomers **2**–**5**, modifications from the standard synthesis conditions were required, notably for the substitution reaction. Thus, the first substitution reaction between benzyl bromoacetate and *N*-Boc-*N*-methylhydrazine (3.0 equiv) was conducted in water at a concentration of 2.5 M at room temperature (rt) for overnight to afford monomer **1a** in 88% yield after SiO_2_ chromatography [41]. Standard substitution conditions in EtOAc or THF as solvent in the presence of triethylamine did not allow full conversion of the starting bromoacetate at rt or on heating to 50 °C. The further substitution reactions, during peptoid elongation, were carried out in a 1:1 MeOH/H_2_O mixture (1.25 M) at 60 °C, using three equivalents of the Boc-protected hydrazine reagent. These distinct substitution conditions, together with standard acylation conditions in solution (Scheme 1) allowed us to reach the pentamer length with good yields for each substitution–acylation submonomer cycle (from 56 to 76% yield, Supporting Information File 1, Scheme S1). All compounds were acetylated at the *N*-terminus followed by removal of the Boc protecting groups to obtain peptoids **1**–**5** with high purity at the scale of several hundred milligrams (Table 1). Synthesis details are provided in Supporting Information File 1, along with analysis data. The main limitation of this synthetic route is the somewhat delicate purification of the products after the substitution step, due to the close polarities of the products and starting hydrazine reagent. So we turned our attention to a fragment-based coupling approach for synthesizing the hexamer peptoid **6**.

**Table 1:** Structures, purity, retention time, and calculated and observed masses for peptoids **1**–**6**.

peptoid	sequence	% purity^a^	retention time	calculated mass	observed mass

**1**	Ac-*N*NMe-OBn	100	8.18	236.1161	237.1234 [M + H]^+^
**2**	Ac-(*N*NMe)_2_-OBn	98	7.71	322.1641	323.1714 [M + H]^+^
**3**	Ac-(*N*NMe)_3_-OBn	97	7.57	408.2121	409.2194 [M + H]^+^
**4**	Ac-(*N*NMe)_4_-OBn	93	7.55	494.2601	495.2674 [M + H]^+^
**5**	Ac-(*N*NMe)_5_-OBn	92	7.55	580.3081	581.3152 [M + H]^+^
**6**	Ac-(*N*NMe)_6_-OBn	98	7.85	666.3562	667.3625 [M + H]^+^

^a^Determined from the HPLC UV trace at 214 nm (conditions in Supporting Information File 1).

**Scheme 2:**
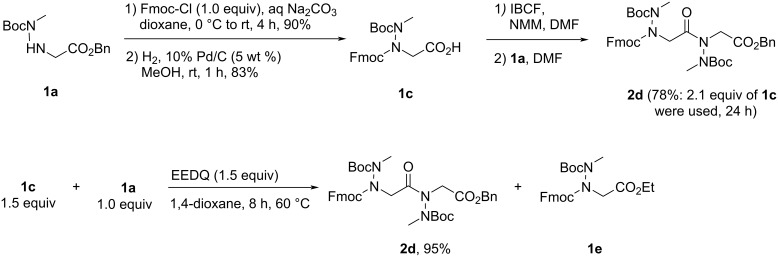
Evaluation of the efficiency of mixed anhydride methods by coupling of **1a** and **1c**.

Thus, several coupling methods were evaluated, initially starting from the hydrazine and acid monomers **1a** and **1c**, respectively (Scheme 2). The *N*-Fmoc-protected acid partner **1c** was readily prepared from **1a** under standard conditions. After a few unsuccessful attempts of coupling using the azabenzotriazole-based coupling reagent HATU [42] or via the formation of an acid chloride with thionyl chloride, we turned to the mixed anhydride activating method using isobutyl chloroformate (IBCF) in the presence of *N*-methylmorpholine (NMM) at 0 °C in DMF for 10 min, followed by the addition of hydrazine **1a** [43–44]. The best results were obtained with two equivalents of preformed mixed anhydride, pure dimer **2d** being isolated in 78% yield after chromatography. The use of stoichiometric amounts of both partners led to incomplete conversion, with about 1/3 of the starting hydrazine being recovered after two days of reaction. We then tested 2-ethoxy-1-ethoxycarbonyl-1,2-dihydroquinoline (EEDQ), in dioxane, a method for in situ generation of a mixed anhydride [45]. This method gave the highest coupling yield to dimer **2d**, but again with the condition of using an excess of the acid partner, as some of it was consumed to give the byproduct ethyl ester **1e**. Dimer **2d** was obtained for example in 95% yield from 1.5 equivalents of acid and carrying out the reaction at 60 °C. The formation of byproduct **1e** was also observed at room temperature and attempts to trap the ethanol released in the reaction by molecular sieves did not give any improvement.

**Scheme 3:**
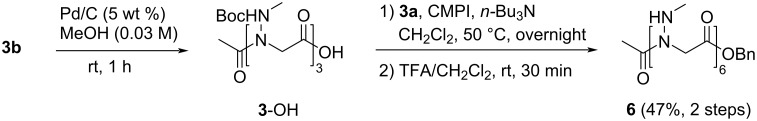
(3 + 3) segment coupling of trimers **3**-OH onto trimer hydrazine **3a**.

Finally, we tested *N*-methyl-2-chloropyridinium iodide (Mukaiyama reagent) [46] by applying the method to a (3 + 3) segment coupling of the *N*-acetylated trimer **3**-OH onto trimer hydrazine **3a**, in equimolar proportions, resulting directly in hexamer **6** after removal of the Boc groups (Scheme 3). In this way, the desired peptoid **6** was isolated in a satisfactory yield of 47% (2 steps) after coupling and TFA-mediated Boc removal.

### Structural characterization of *N*-methylamino peptoid oligomers

#### X-ray diffraction analysis of peptoid dimer **2**

**Figure 3:**
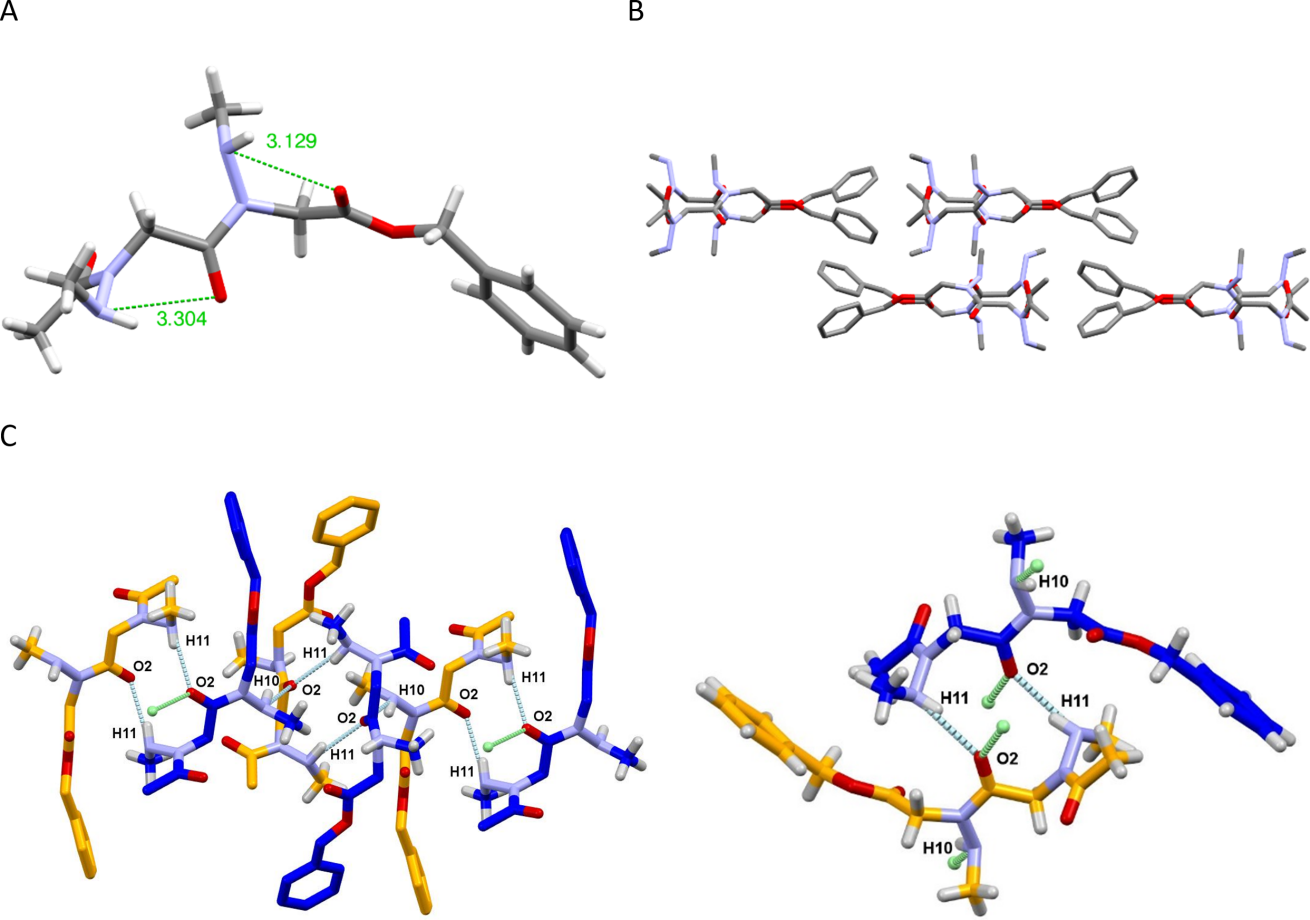
X-ray crystal structure of peptoid dimer **2**: (A) single molecule; (B) unit cell, view along *b* axis (hydrogen atoms removed for clarity); (C) overview of the hydrogen bonding network (conformation A depicted in blue, conformation B in orange), hydrogen bonds (light blue dashed lines), hanging hydrogen bonds (light green dashed lines).

Peptoid dimer **2** was crystallized by slow evaporation from chloroform, and its high resolution structure was determined by X-ray crystallography. The crystal structure of dimer **2** confirms the *trans* geometry of the two amide bonds (Figure 3A). The unit cell contains eight molecules, including two groups of four identical molecules (Figure 3B), the conformation of the first group (conformation A, Table 2) being the mirror image of that observed for the second group of molecules (conformation B). In the crystal lattice, each molecule establishes four intermolecular CO···HN hydrogen bonds. Only the inter-residue carbonyl (oxygen atom labeled O2, Figure 3C) participates in this network, making two hydrogen bonds with two different molecules and different NH groups (labeled H10 and H11, Figure 3C). The φ and ψ dihedral angle values are comparable to those measured by X-ray diffraction of monomers bearing benzylamino side chains [40], and of an *N*-aryl [33] and *N*-hydroxy peptoid dimers [36]. The latter dimer with φ angles of opposite sign was shown to form a unique sheet-like secondary structure, whereas molecular modeling showed that *N*-aryl peptoid oligomers composed of monomers in the same conformation (as observed in the crystal of dimer **2**) might adopt right or left-handed helical conformations that resemble the polyproline type II helix. The χ1 dihedral angles, −111.6 and −116.1 for residues 1 and 2, respectively (conformation A), are identical to one another in sign, a sign that corresponds to that of the φ dihedral angles. This results in an almost perpendicular orientation of the N–H bond with respect to the amide plane and a proper orientation of the NH and CO groups of the same residue for intramolecular hydrogen bonding, although the N^…^O distances of 3.13 and 3.30 Å are slightly above the accepted thresholds.

**Table 2:** Dihedral angles observed in dimer **2** crystal structure.

	residue	ω (°)	φ (°)	ψ (°)	χ1 (°)	d ^i^Nα---OC^i^ (Å)

conformation A	1	−176.5	−87.7	166.7	−111.6	3.30
	2	−173.7	−103.3	167.1	−116.1	3.13
conformation B	1	176.5	87.7	−166.7	111.6	3.30
	2	173.7	103.3	−167.1	116.1	3.13

Dihedral angles definition: ω [Cα(i−1); C(i−1); N; Cα], φ [C(i−1); N; Cα; C], ψ [N; Cα; C; N(i+1)], χ_1_ [C(i−1); N; N_α_; C_β_].

#### Conformational analysis and self-assembling properties

**Figure 4:**
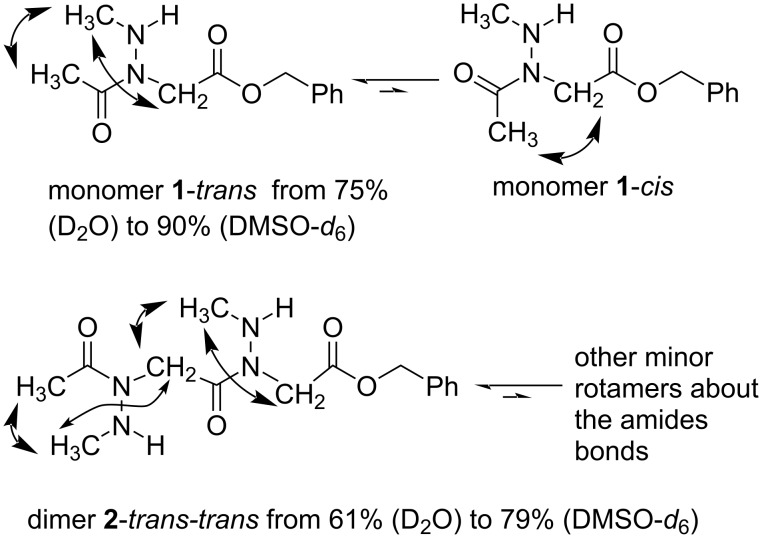
NOE effect interaction observed in the 2D-NOESY spectra of monomer **1** and dimer **2** in DMSO-*d*_6_.

The ^1^H NMR analysis of monomer **1** in various solvents including CDCl_3_, CD_3_CN, C_6_D_6_, CD_3_OD, D_2_O, and DMSO-*d*_6_ showed two sets of resonances in proportions varying from 75:25 to 90:10 (Table 3). These two sets of signals were characterized as the backbone *cis* and *trans*-amide rotamers using NOESY experiments (Figure 4 and Supporting Information File 1). Specifically, in DMSO-*d*_6_, the predominant rotamer of monomer **1** is characterized by a NOE cross-peak between the acetyl methyl group and side chain methyl group, indicative of a *trans*-amide bond geometry. A NOE cross-peak between the backbone methylene group and side chain methyl group is also observed in this *trans*-rotamer. For the second set of resonances, the presence of a NOE cross-peak between the backbone methylene group and acetyl methyl group confirmed the presence of a *cis*-amide bond. Similarly, dimer **2** and trimer **3** showed similar correlation patterns to those observed in the monomer involving two and three *trans*-amide bonds, respectively, for the predominant rotamers (Figure 4 and Supporting Information File 1).

**Table 3:** Average *trans* rotamer proportions (% *trans*) and *K*_cis/trans_ values in peptoids **1** and **2**, calculated from the integration of ^1^H NMR spectra in various solvents (8 mM).

	CDCl_3_		C_6_D_6_		(CD_3_)_2_SO		CD_3_CN		CD_3_OD		D_2_O	
	% trans	*K*_cis/trans_	% trans	*K*_cis/trans_	% trans	*K*_cis/trans_	% trans	*K*_cis/trans_	% trans	*K*_cis/trans_	% trans	*K*_cis/trans_

**1**	82	0.22^a^	84	0.18^a^	90	0.11^a^	85	0.17^b^	89	0.13^a^	75	0.34^c^
**2**	77	0.31^a^	^d^	^d^	79	0.26^a^	78	0.29^a^	75	0.33^a^	61	0.63^b^

^a^Calculated by averaging the integrations of 3 signals from the NMR spectrum. ^b^Calculated by averaging the integrations of 2 signals from the NMR spectrum. ^c^Calculated by averaging the integrations of 4 signals from the NMR spectrum. ^d^Not measurable.

We could also show that the two amides of dimer **2** have approximately the same *cis*/*trans* ratio, at least in CDCl_3_ and CD_3_OD. This suggests that the same may be true for longer oligomers. As the oligomer elongates, shoulders that may represent different rotamers in small proportions appear in the NMR spectra, but one isomer remains present predominantly. More specifically, in the case of trimer **3**, tetramer **4**, pentamer **5**, and hexamer **6**, we were able, for example, to establish that the majority rotamer is present at 70%, 84%, 80%, and 86%, respectively in DMSO-*d*_6_, based on the integration of the backbone methylene signals. Overall, DMSO seems to be the most structuring solvent for these oligomers, followed very closely by methanol and acetonitrile.

Having confirmed that the *N*-(methylamino)amide bonds of the synthesized oligomers are mainly in the *trans* conformation, it remained to be seen whether the NH of the side chains participate in hydrogen bonding either intra- or intermolecularly.

**Figure 5:**
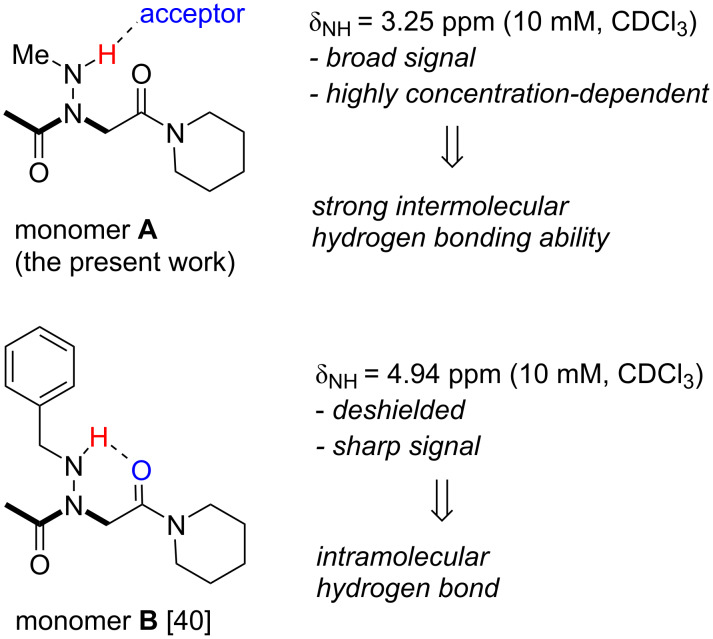
Comparison of monomers **A** and **B** with respect to their ability to form intramolecular and intermolecular hydrogen bonds.

The ^1^H NMR resonances of the N–H groups of peptoids **1**–**5** displayed broad signals at room temperature in CDCl_3_. This is likely due to proton exchange between various NH groups in low polar solvents or to exchanges between the NH protons and protons of residual H_2_O. In weakly polar aprotic solvents such as CDCl_3_, resonance broadening may also be due to intermolecular association. The chemical shift of donor protons (D–H) involved in intermolecular hydrogen bonds is generally very sensitive to concentration change. A variable concentration ^1^H NMR study of monomer **A** and trimer **3** was carried out in CDCl_3_. The piperidinyl amide-capped monomer **A** (Figure 5, see Supporting Information File 1 for synthesis), was preferred to monomer **1** to allow an unbiased comparison with a previous study from the Proulx group (monomer **B**, Figure 5) [40]. A large variation in the chemical shift of NH was observed over a concentration range of 2–50 mM for monomer **A** in CDCl_3_ (Δδ = 3.09 ppm, Supporting Information File 1, Figure S1), suggesting intermolecular hydrogen bonding, in sharp contrast to the Δδ = 0.01 ppm measured for the piperidinyl amide-capped *N*-benzylamino glycine monomer **B**, which is further characterized by a narrow NH signal in CDCl_3_ or DMSO-*d*_6_ (Figure 5). The same behavior was observed in the case of trimer **3** (Δδ = 2.61 ppm), again suggesting intermolecular hydrogen bonding. The minimal steric hindrance of the side chain *N*-methyl group thus seems favorable to intermolecular associations, as observed in the case of *N*-hydroxypeptoids [36]. Another notable difference between monomers **A** and **B** is the large difference in the chemical shift of the NH group at a given concentration (3.25 ppm for **A** and 4.92 ppm for **B** at 10 mM in CDCl_3_). The significant deshielding of the NH chemical shift in **B**, as compared to **A**, is consistent with intramolecular hydrogen bond interaction, which is accompanied by a reduction of the exchange rate of the NH proton. In DMSO, we observe reduced NH linewidths, consistent with the fact that this solvent has strong hydrogen bonding and solvation abilities which reduce significantly proton exchange. Overall, the NMR study suggests that the *N*-methylamino glycine monomer and oligomers have a strong propensity to form intermolecular hydrogen bonds, an interesting and sought-after property for self-assembling and interaction with biological targets.

Neat peptoids **1**–**6** were also characterized by Fourier-transform infrared spectroscopy. The spectra show two distinct N–H stretching bands in the 3500–3200 cm^−1^ region whose relative intensity varies with chain elongation. A first band, the more intense of the two, is observed at about 3300 cm^−1^. A higher energy band of much lower intensity at about 3450–3500 cm^−1^ is also observed, especially from the trimer stage. We can assume that the lower energy bands (3300 cm^−1^) are due to hydrogen-bonded N–H while the higher energy bands could be attributed to non-hydrogen bonded NH. This observation may indicate that not all NHs can form hydrogen bonds within an assembly, and that this is dependent on the size of the oligomers. Also, the region of the spectra corresponding to the C=O stretching (1800–1600 cm^−1^) show a band at 1743 cm^−1^ corresponding to C=O stretching of the ester and a band at 1648 cm^−1^ which increases with the oligomer length, corresponding to the amide I C=O stretching of the *N*-(methylamino)amides functions. Preliminary transmission electron microscopy (TEM) analysis of dimer **2** seems to suggest ordered self-assembled multilayered structures (Figure S5 in Supporting Information File 1). This observation now needs to be studied in detail.

### Computational studies

For the theoretical calculations, acetyl *N*-(methylamino)dimethylamide model peptoids (Ac-*N*(NMe)*n*-NMe_2_) were used instead of the corresponding synthesized benzyl esters. The model structures were generated using the coordinates extracted from the single crystal X-ray diffraction data of dimer **2**. Geometry optimizations were carried out with the B3LYP/6-31G(d,p) basis set as implemented in Gaussian 16, using tight convergence criteria (opt = tight) in chloroform (scrf = (solvent = chloroform)).

We first examined the preferred amide-bond geometry by a relaxed potential energy surface (PES) scan about the ω dihedral angle with a scan interval of 10° in 35 steps from −180° to 180°. The global minimum energy was observed at −174° and +176°, with a substantial energetic preference for the *trans*-amide bond of 6.5 kcal/mol, supporting the *trans* geometry determined from NMR and X-ray diffraction experimental data (Figure S3 in Supporting Information File 1). To understand how the methylamino-substituent will be oriented on oligo-*N*NMe peptoids, we have also performed a χ1 angle relaxed PES scan of acetyl-*N-*methylaminoglycine-dimethylamide (Figure S4 in Supporting Information File 1). The lowest energy was found for χ1 = 124°, an angle value slightly higher than those found in the crystallographic structure of compound **2** (111 and 116° for conformation B, Table 2). This difference may be a result of the 6-membered intramolecular hydrogen bond formed in the monomer model (d N^…^O = 2.88 Å, 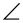
^i^N–H^…^O = 125°, Table S1 in Supporting Information File 1). Two hydrogen bonds were also present upon geometry optimization of the dimer model Ac-*N*-(NMe)_2_-NMe_2_ (Table S2 in Supporting Information File 1).

**Figure 6:**
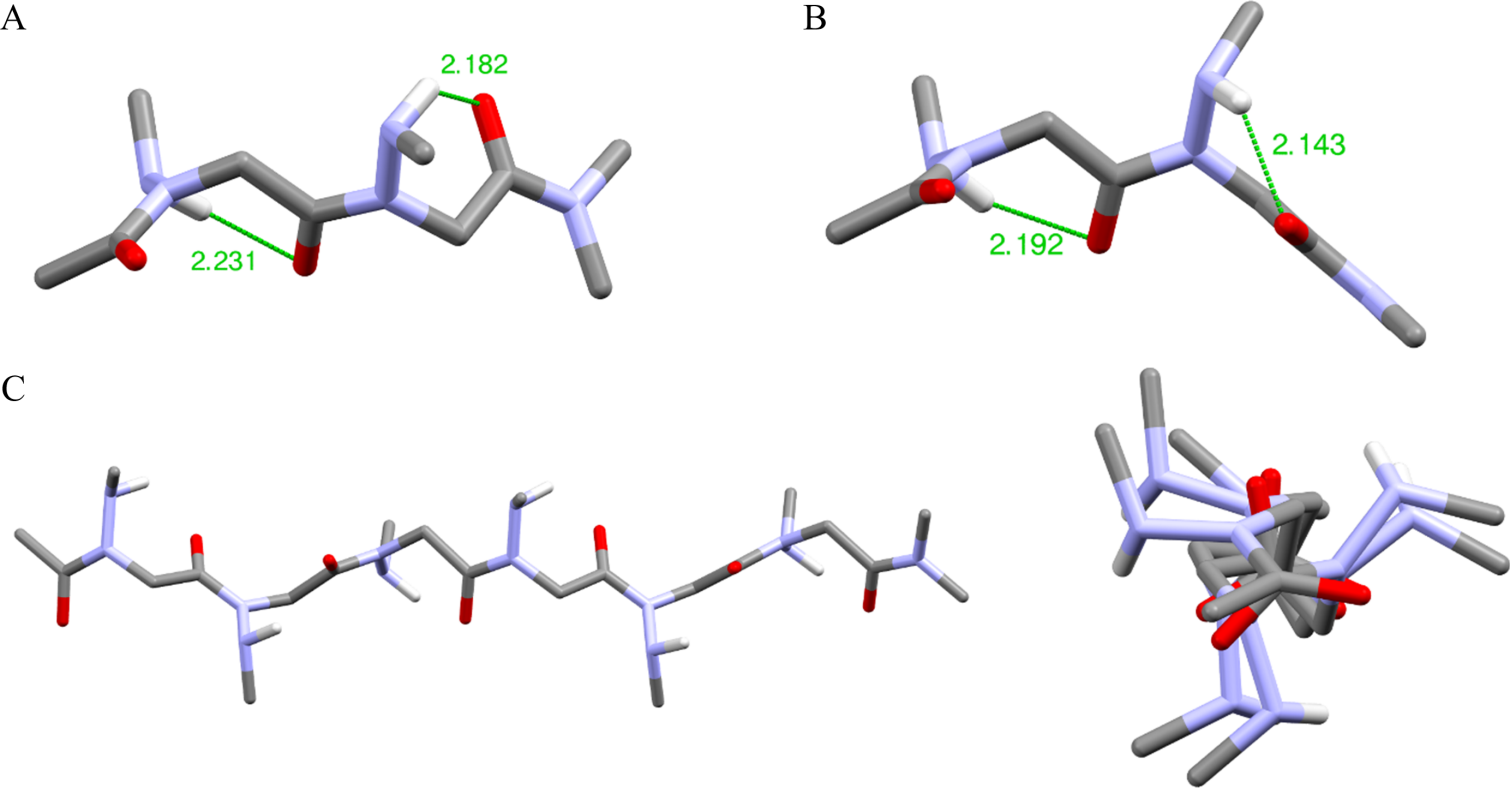
Model structure of *N*-(NMe)glycine peptoid. (A) dimer in the repeating (pp) conformation; (B) dimer in the alternating (pm) conformation; (C) hexamer in the repeating (p)_6_ conformation: side view (left), perpendicular to helix axis (right).

In the crystallographic structure of dimer **2**, the two constitutive monomers of a molecule are in the same conformation with either two positive values of the φ angle (conformation B) or two negative values for the mirror image conformation A. These two local conformations could exist at each monomer position, leading to a potential mixture of secondary structures. We therefore calculated the relative energy difference between the structure of a dimer consisting of two monomers in the same conformation (pp) with that of a dimer structure with monomers in two different mirror image conformations (pm) (Figure 6A and B). We found that the repeating (pp) conformation is only favored by 0.65 kcal·mol^−1^ over the (pm) alternated conformation, suggesting that these two conformations could coexist in solution. Furthermore, the (pm) dimeric model adopt a structure analogous to the solid-state structure of an *N*-hydroxypeptoid dimer, with very similar dihedral angles and φ angles opposite to one another in sign [36]. Calculation was also carried out at the hexamer length considering only the repeating (p)_6_ and alternating (pm)_3_ regular conformations (Table S3 in Supporting Information File 1). It resulted that the repeating conformation (p)_6_ is most stable by 2.9 kcal·mol^−1^ and forms an extended right-handed helical conformation with backbone torsion angle (φ, ψ) values in vicinity of (100°, −173°), approximately 3 residues per turn, and a helical pitch of 10 Å (Figure 6C). The structure resembles the type II polyproline helix, with a slightly larger pitch.

## Conclusion

This report describes for the first time the synthesis of peptoid oligomers consisting of consecutive *N*-alkylamino units, with the aim of creating peptoid oligomers with *trans*-amide linkages. For this first study, we focused on solution-phase synthesis of *N*-(methylamino)peptoids using a submonomer protocol and evaluated few segment-based coupling methods. The optimizations made with respect to the standard submonomer synthetic conditions will be useful for developing solid-phase synthesis and access longer and more diverse *N*-(alkylamino)peptoid oligomers in the future. NMR analysis of the synthesized oligomers **1**–**6** in various protic and aprotic solvents and of varying polarity indicates that the *N*-(methylamino)glycine units favor *trans*-amide bonds in proportions up to 90% in DMSO-*d*_6_. Adoption of *trans*-amide bonds was confirmed in the crystal structure of dimer **2**. In addition to the control of the amide bond geometry by the *N*-methylamino side chain, the presence of a hydrogen bond NH donor group is a key element in controlling the main-chain conformation and side chains orientation. It should be noted that to date, the presence of D–H donor groups on the side chains has been little exploited to control the folding and intermolecular association properties. The NMR study shows that this peptoid family has a strong propensity to form intermolecular assemblies in solution, whatever the nature of the solvent. TEM images of dimer **2** in water may suggest the formation of multilayered assemblies that deserve to be studied now in more detail. The crystal structure of dimer **2** also reveals the presence of intermolecular hydrogen bonding networks that could potentially be found in solution. It is also interesting to note that despite the lack of chirality, the two residues of peptoid **2** adopt the same conformation in the crystal, reminiscent to that found in helical PPII-like structures. Interestingly, peptoids containing *N*-(methylamino)amides are soluble in water and are capable of forming hydrogen bonds, two interesting characteristics for designing peptidomimetic molecules or biomaterials. Additional efforts are now focused on introducing chirality, which will provide a better understanding of their structural properties, including their potential to form PPII-like helices.

## Supporting Information

File 1 Experimental procedures, HPLC analytical data, NMR spectra and variable concentration study, infrared spectra, full X-ray data for **2**, computation data, and additional TEM images for compound **2**.

File 2 Crystallographic information file (CIF) for compound **2**, CCDC deposition number 2167472.
