# Supplementary material for: First series of N-alkylamino peptoid homooligomers: solution phase synthesis and conformational investigation
Source: Beilstein J Org Chem. 2022 Jul 14;18:845–54. doi: 10.3762/bjoc.18.85 (PMC9296984; doi:10.3762/bjoc.18.85)
Supplement: File 1 — Experimental procedures, HPLC analytical data, NMR spectra and variable concentration study, infrared spectra, full X-ray data for 2, computation data, and additional TEM images for compound 2. [file Beilstein_J_Org_Chem-18-845-s001.pdf]

BEILSTEIN JOURNAL OF ORGANIC CHEMISTRY

# Supporting Information

for

# First series of *N*-alkylamino peptoid homooligomers: solution phase synthesis and conformational investigation

Maxime Pypec, Laurent Jouffret, Claude Taillefumier and Olivier Roy

*Beilstein J. Org. Chem.* **2022,** *18,* 845–854. doi:10.3762/bjoc.18.85

# Experimental procedures, HPLC analytical data, NMR spectra and variable concentration study, infrared spectra, full X-ray data for 2, computation data, and additional TEM images for compound 2

License and Terms: This is a supporting information file under the terms of the Creative Commons Attribution License (https://creativecommons.org/licenses/by/4.0). Please note that the reuse, redistribution and reproduction in particular requires that the author(s) and source are credited and that individual graphics may be subject to special legal provisions.
The license is subject to the *Beilstein Journal of Organic Chemistry* terms and conditions: (https://www.beilstein-journals.org/bjoc/terms)

**Table of contents**


1. General methods ........ S2
2. Synthesis of peptoid oligomers **1**–**6** ........ S3
   a) Submonomer synthesis of peptoids **1**–**5** ........ S3
   b) General procedures ........ S3
   c) Segment-coupling reactions ........ S7
3. X-ray crystallographic data for peptoid **2** ........ S9
4. Analytical HPLC chromatograms of peptoids **1**–**6** and monomer **A** ........ S15
5. NMR spectra ........ S18
6. Variable concentration study ........ S41
7. Infrared spectra ........ S42
8. Computation ........ S44
   a) Geometry optimization of monomer model Ac-*N*NMe-$NMe_2$ ........ S44
   b) Geometry optimization of dimer model Ac-(*N*NMe)$_2$-$NMe_2$ ........ S46
   c) Geometry optimization of hexamer model Ac-(*N*NMe)$_6$-$NMe_2$ ........ S46
9. Transmission electron microscopy (TEM) ........ S46

## 1. General methods

Anhydrous THF, $CH_2Cl_2$, DMF (amine free), and all other solvents and chemicals were obtained from commercial sources and were used as received. NMR spectra were recorded on a 400 MHz Bruker Avance III HD spectrometer or a 500 MHz Bruker AC-500 spectrometer. Chemical shifts are referenced to the residual solvent peak, and *J* values are given in hertz. The following multiplicity abbreviations are used: s, singlet; d, doublet; t, triplet; q, quartet; m, massif or multiplet. Where applicable, assignments were based HMBC, HSQC, and $^{13}C$ NMR experiments. Thin-layer chromatography (TLC) was performed on Machery-Nagel Alugram TLC aluminum sheets, silica gel 60 and F254. Progression of reactions was, when applicable, followed by TLC. Visualizing of spots was effected with UV light and/or vanillin in EtOH/$H_2SO_4$. Flash chromatography was performed manually with VWR silica gel 40–63 µm or on Büchi C-815 Flash Pure chromatography system equipped with FlashPure ID silica cartridge. HRMS was recorded on a Micromass Q-Tof Micro (3000 V) apparatus or a Q Exactive Quadrupole-Orbitrap Mass Spectrometer. Liquid chromatography–mass spectrometry was recorded on a Q Exactive Quadrupole-Orbitrap mass spectrometer coupled to a UPLC Ultimate 3000 (Kinetex EVO C18); 1.7 µm; 100 mm × 2.1 mm column with a flow rate of 0.45 mL/min with the following gradient: a linear gradient of solvent B from 5 to 95% over 7.5 min (solvent A = $H_2O$ + 0.1% formic acid, solvent B = acetonitrile + 0.1% formic acid) equipped with a DAD UV–vis 3000 RS detector. X-ray data were collected at 100 K with an Oxford Diffraction Xcalibur 2 diffractometer equipped with a copper microsource ($\lambda$ = 1.5418 Å). HPLC analysis was performed on an Agilent 1100 series system composed of an autosampler G1329A, a G1379A degasser, a G1311A quaternary pump, a TCC-100 thermostated column compartment at 25 °C and a G1315A Diode Array Detector (DAD). Infrared analysis was recorded on a SHIMADZU FTIR-8400S spectrometer equipped with a Pike Technologies MIRacle™ ATR. Wavenumbers ($\nu$) are expressed in $cm^{-1}$. Spectra were recorded with 16 scans, between 800 and 4000 $cm^{-1}$.

## 2. Synthesis of peptoid oligomers 1–6

### a) Submonomer synthesis of peptoids 1–5

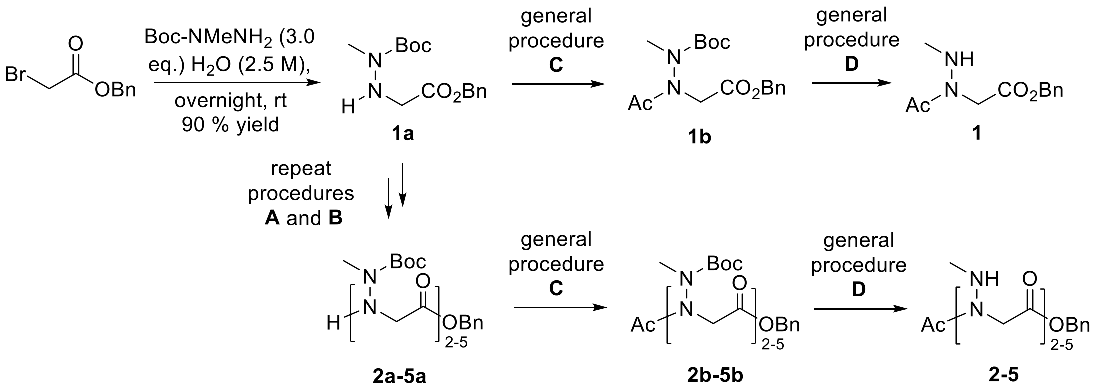


| Submonomer cycle (2 steps) | Yields (%) |
|---|---|
| **1a → 2a** | 76 |
| **2a → 3a** | 67 |
| **3a → 4a** | 56 |
| **4a → 5a** | 59 |

**Scheme S1.** Stepwise synthesis of peptoid **1–5** and yields for each acylation-substitution submonomer cycle.

### b) General procedures

#### General procedure A (peptoid bromoacylation)

To a solution of hydrazine **1a–4a** (1.0 equiv, 0.2 M/THF) and $Et_3N$ (1.2 equiv) under argon atmosphere at −10 °C, was slowly added bromoacetyl bromide (1.05 to 1.2 equiv) and the reaction mixture was stirred for 15 to 30 min at −10 °C. The reaction mixture was then diluted with ethyl acetate (10 mL/mmol) and the formed ammonium salts were filtered. The filtrate was evaporated, resulting in new salts, which were again filtered under vacuum. The filtrate was dried under vacuum and the crude bromoacylated peptoid was purified by column chromatography on silica gel.

#### General procedure B (substitution of the bromoacetamide intermediate)

To a solution of bromoacetamide (1.0 equiv, 1.25 M/1:1 $H_2O$/MeOH) was added 1-Boc-1-methylhydrazine (3.0 equiv) and the solution was stirred overnight at 60 °C. The reaction mixture was then diluted with water and methanol was evaporated under vacuum. The peptoid was then extracted three times with ethyl acetate and the organic layer washed with water (3 ×) and brine. The organic phase was dried over $MgSO_4$, filtered and concentrated under reduced pressure. The crude was purified by column chromatography on silica gel.

#### General procedure C (acetylation)

To a solution of peptoid (1.0 equiv, 0.2 M/EtOAc) and $Et_3N$ (4.0 equiv) was added acetic anhydride (8.0 equiv). The mixture was stirred for 48 h at room temperature. After completion of the reaction, the solution was diluted with ethyl acetate and successively washed with aq. saturated $NaHCO_3$ (3 ×), water, aq. saturated $NH_4Cl$ (3 ×), water and brine. The organic phase was dried over $MgSO_4$, filtered, and concentrated under reduced pressure. The crude acetylated peptoid thus obtained was purified by flash chromatography on silica gel.

### General procedure D (Boc deprotection)

The protected peptoid was solubilized in a TFA/$CH_2Cl_2$ 1:1 mixture at 0 °C. The mixture was then stirred at room temperature for 30 min. After that, the solution was concentrated under vacuum, the resulting oil was redissolved in $CH_2Cl_2$, and the solution was evaporated again under vacuum. This operation was repeated several times to eliminate the remaining traces of TFA. The final compounds were purified by flash chromatography on silica gel.

### Monomer 1

Benzyl bromoacetate (0.50 mL, 3.14 mmol, 1.0 equiv) was reacted with 1-Boc-1-methylhydrazine (1.4 mL, 9.42 mmol, 3 equiv) in 1.26 mL (2.5 M) of water, at room temperature, overnight. The mixture was then diluted with water and the desired compound was extracted three times into ethyl acetate. The organic layer was washed with water (3 ×) and brine, dried over $MgSO_4$, and evaporated under reduced pressure. The remaining residue was purified on silica gel (cyclohexane/EtOAc 4:1) to produce 92% of monomer **1a**. The latter was acetylated (general procedure **C**), to yield the Boc-protected monomer **1b** in 91% yield after purification on silica gel (cyclohexane/EtOAc 4:1). Finally, the Boc group was removed following general procedure **D**, yielding monomer **1** as a colourless oil (594 mg, 2.52 mmol) in an overall yield of 83% after purification on silica gel (cyclohexane/EtOAc 3:2).

$R_f$ = 0,27 (Cyclohexane/EtOAc 1:1), HRMS (TOF MS ES$^+$): *m/z* calcd for $C_{12}H_{16}O_3N_2$ $[M+H]^+$ 237.1161, found 237.1234. $^1H$ NMR (400 MHz, DMSO-d6): $\delta$ (ppm): 1.96 (s, 0.32H, Ac *cis* rotamer), 2.05 (s, 2.68H, Ac *trans* rotamer), 2.40 and 2.44 (2×s, 3H, C***H₃***NH), 4.22 (s, 1.80H, NC$\boldsymbol{H_2}$C=O *trans* rotamer), 4.35 (s, 0.20H, NC$\boldsymbol{H_2}$C=O *cis* rotamer), 4.92 (m, 1H, N**H**), 5.13 (s, 1.80H, C$\boldsymbol{H_2}$Ph *trans* rotamer), 5.17 (s, 0.20H, C$\boldsymbol{H_2}$Ph *cis* rotamer), 7.35-7.37 (m, 5H, $C_6H_5$).

$^{13}C$ NMR (100 MHz, $CDCl_3$): $\delta$ (ppm): 20.5 ($CH_3$, Ac *trans* rotamer), 21.0 ($CH_3$, Ac *cis* rotamer), 35.3 ($CH_3$, $\boldsymbol{C}H_3$NH *trans* rotamer), 36.8 ($CH_3$, $\boldsymbol{C}H_3$NH *cis* rotamer), 43.9 ($CH_2$, N$\boldsymbol{C}H_2$C=O *trans* rotamer), 52.3 ($CH_2$, N$\boldsymbol{C}H_2$C=O *cis* rotamer), 67.0 ($CH_2$, $\boldsymbol{C}H_2$Ph *trans* rotamer), 67.4 ($CH_2$, $\boldsymbol{C}H_2$Ph *cis* rotamer), 128.3, 128.5, 128.6 (5 CH, $C_6H_5$), 135.2 ($C_{ipso}$, $C_6H_5$), 168.9 (C, $\boldsymbol{C}O_2$Bn *cis* rotamer), 169.3 (C, $\boldsymbol{C}O_2$Bn *trans* rotamer), 170.3 (C, $CH_3\boldsymbol{C}$(=O)N *cis* rotamer), 174.4 (C, $CH_3\boldsymbol{C}$(=O)N *trans* rotamer).

### Dimer 2 (stepwise synthesis from monomer 1a)

Monomer **1a** (0.958 g, 2.85 mmol, 1equiv) was bromoacylated according to general procedure **A** (purification $SiO_2$ column, cyclohexane/EtOAc 4:1). Next, the formed bromoacetamide intermediate was engaged in the substitution step with 1-Boc-1-methylhydrazine according to general procedure **B**, yielding dimer **2a** in 76% yield (2 steps) after column chromatography ($SiO_2$, cyclohexane/EtOAc 4:1). Acetylation of **2a** (general procedure **C**), gave the Boc-protected dimer **2b** in 82% yield after column chromatography ($SiO_2$, cyclohexane/EtOAc 7:3). Finally, the Boc groups were removed following general procedure **D**, to give dimer **2** as a colourless oil which solidified upon redissolution in chloroform and evaporation (342 mg, 1.06 mmol) in an overall yield of 37% after $SiO_2$ chromatography ($CH_2Cl_2$/MeOH 98:2).

$R_f$ = 0.21 ($CH_2Cl_2$/MeOH 98:2), HRMS (TOF MS ES$^+$): *m/z* calcd for $C_{15}H_{22}N_4O_4$ $[M+H]^+$ 323.1641, found 323.1714. $^1H$ NMR (400 MHz, DMSO-d6): $\delta$ (ppm): 1.84 (s, 0.4H, Ac), 2.03-2.05 (m, 2.6H, Ac), 2.40-2.46 (m, 6H, 2×C$\boldsymbol{H_3}$NH), 4.25-4.28 (m, 2H, C$\boldsymbol{H_2}CO_2$Bn), 4.46-4.48 (m, 2H, NC$\boldsymbol{H_2}$C=O), 4.62 (m, 1H, NH), 4.93 (m, 1H, NH), 5.14(s, 1.9H, C$\boldsymbol{H_2}$Ph), 5.18 (s, 0.1H, C$\boldsymbol{H_2}$Ph), 7.33-7.38 (m, 5H, $C_6H_5$).

$^{13}C$ NMR (100 MHz, $CDCl_3$) of the major conformer: $\delta$ (ppm): 20.7 ($CH_3$, Ac), 34.9, 35.2 ($CH_3$, 2×NH$\boldsymbol{C}H_3$), 42.7, 44.0 ($CH_2$, 2×N$\boldsymbol{C}H_2$C=O), 67.3 ($CH_2$, $\boldsymbol{C}H_2$Ph), 128.4, 128.6, 128.7 (5$\boldsymbol{C}$H, $C_6H_5$), 135.0 ($C_{ipso}$, $C_6H_5$), 168.8 (C, $\boldsymbol{C}O_2$Bn), 171.7 (C, $NCH_2\boldsymbol{C}$(=O)N), 175.0 (C, $CH_3\boldsymbol{C}$(=O)N).

**Trimer 3 (stepwise synthesis from dimer 2a)**

Dimer **2a** (1.356 g, 2.59 mmol, 1 equiv) was bromoacylated according to general procedure **A**, followed by substitution of the bromine atom with 1-Boc-1-methylhydrazine according to general procedure **B** to produce 67% over two steps of trimer **3a**. Acetylation of **3a** (general procedure **C**), gave the Boc-protected trimer **3b** in 84% yield after column chromatography ($SiO_2$, cyclohexane/EtOAc 7:3). Finally, the Boc groups were removed following general procedure **D**, to give trimer **3** as a pale yellow oil (347 mg, 0.850 mmol) in an overall yield of 33% after $SiO_2$ chromatography ($CH_2Cl_2$/MeOH 97:3).

$R_f$ = 0.13 ($CH_2Cl_2$/MeOH 98:2), HRMS (TOF MS ES$^+$): *m/z* calcd for $C_{18}H_{29}N_6O_5$ $[M+H]^+$ 409.2121 found 409.2194. $^1H$ NMR (400 MHz, DMSO-d6): $\delta$ (ppm): 1.92 and 2.03-2.05 (m, 3H, Ac), 2.38-2.47 (m, 9H, 3×C***H₃***NH), 4.27-4.29 (m, 2H, C***H₂***CO₂Bn), 4.46-4.57 (m, 4H, 2×NC***H₂***C=O), 4.95 (m, 1H, NH), 5.14(s, 1.95H, C***H₂***Ph), 5.19 (s, 0.05H, C***H₂***Ph), 7.33-7.38 (m, 5H, $C_6H_5$).

$^{13}C$ NMR (100 MHz, $CDCl_3$) of the major conformer: $\delta$ (ppm): 20.6 ($CH_3$, Ac), 34.8, 34.9, 35.1 ($CH_3$, 3×NH***C***H₃), 43.0, 43.2, 44.1 ($CH_2$, 3×N***C***H₂C=O), 67.3 ($CH_2$, ***C***H₂Ph), 128.4, 128.6, 128.7 (5***C***H, $C_6H_5$), 134.9 ($C_{ipso}$, $C_6H_5$), 168.8 (C, ***C***O₂Bn), 171.3, 171.8 (C, 2×NCH₂***C***(=O)N), 175.1 (C, CH₃***C***(=O)N).

**Tetramer 4 (stepwise synthesis from trimer 3a)**

Trimer **3a** (0.992 g, 1.49 mmol) was bromoacetylated according to general procedure **A**, followed by substitution of the bromine atom with 1-Boc-1-methylhydrazine according to general procedure **B** to produce 56% over two steps of tetramer **4a** (0.709 g, 0.83 mmol). Acetylation of **4a** (general procedure **C**), gave the Boc-protected tetramer **4b** (0.610 g, 0.68 mmol) in 82% yield after column chromatography ($SiO_2$, cyclohexane/EtOAc 7:3). Finally, the Boc groups were removed following general procedure **D**, to give tetramer **4** as a pale yellow oil (0.207 g, 0.42 mmol) in an overall yield of 29% after $SiO_2$ chromatography ($CH_2Cl_2$/MeOH 95:5).

$R_f$ = 0.26 ($CH_2Cl_2$/MeOH 95:5), HRMS (TOF MS ES$^+$): *m/z* calcd for $C_{21}H_{35}N_8O_6$ $[M+H]^+$ 495.2601 found 495.2674. $^1H$ NMR (400 MHz, DMSO-d6): $\delta$ (ppm): 1.92-2.06 (m, 3H, Ac), 2.41-2.47 (m, 12H, 4×C***H₃***NH), 4.19-4.30 (m, 2H, C***H₂***CO₂Bn), 4.46-4.55 (m, 6H, 3×NC***H₂***C=O), 4.68-4.96 (m, 4H, 4×NH), 5.14-5.19 (m, 2H, C***H₂***Ph), 7.33-7.38 (m, 5H, $C_6H_5$).

$^{13}C$ NMR (100 MHz, $CDCl_3$) of the major conformer: $\delta$ (ppm): 20.6 ($CH_3$, Ac), 34.8, 34.9, 35.1 ($CH_3$, 4×NH***C***H₃), 43.0, 43.3, 43.4, 44.1 ($CH_2$, 4×N***C***H₂C=O), 67.4 ($CH_2$, ***C***H₂Ph), 128.4, 128.7 (5***C***H, $C_6H_5$), 134.9 ($C_{ipso}$, $C_6H_5$), 168.8 (C, ***C***O₂Bn), 171.2 (C, 3×NCH₂***C***=O).

**Pentamer 5 (stepwise synthesis from tetramer 4a)**

Tetramer **4a** (0.913 g, 1.07 mmol) was bromoacetylated according to general procedure **A**, followed by substitution of the bromine atom with 1-Boc-1-methylhydrazine according to general procedure **B** to produce 59% over two steps of pentamer **5a** (0.657 g, 0.63 mmol). Acetylation of **5a** (general procedure **C**), gave the Boc-protected pentamer **5b** (0.500 g, 0.46 mmol) in 73% yield after column chromatography ($SiO_2$, cyclohexane/EtOAc 7:3). Finally, the Boc groups were removed following general procedure **D**, to give pentamer **5** as colourless foam (0.195 g, 0.33 mmol) in an overall yield of 31% after $SiO_2$ chromatography ($CH_2Cl_2$/MeOH 95:5).

$R_f$ = 0.23 ($CH_2Cl_2$/MeOH 95:5), HRMS (TOF MS ES$^+$): *m/z* calcd for $C_{24}H_{41}N_{10}O_7$ $[M+H]^+$ 581.3081 found 581.3152. $^1H$ NMR (400 MHz, DMSO-d6): $\delta$ (ppm): 1.92-2.03 (m, 3H, Ac), 2.42-2.47 (m, 15H, 5×NHC***H₃***), 4.19-4.30 (m, 2H, C***H₂***CO₂Bn), 4.40-4.48 (m, 8H, 4×NC***H₂***C=O), 4.69-4.95 (m, 5H, 5×NH), 5.14-5.19 (m, 2H, C***H₂***Ph), 7.32-7.38 (m, 5H, $C_6H_5$).

$^{13}C$ NMR (100 MHz, $CDCl_3$) of the major conformer: $\delta$ (ppm): 20.6 ($CH_3$, Ac), 34.8, 35.1 ($CH_3$, 5×NH***C***$H_3$), 43.6, 43.7, 44.4 ($CH_2$, 5×N***C***$H_2$C=O), 67.3 ($CH_2$, ***C***$H_2$Ph), 128.3, 128.6, 128.7 (5***C***H, $C_6H_5$), 135.0 ($C_{ipso}$, $C_6H_5$), 168.9 (C, ***C***$O_2$Bn).

**Monomer A**

To a solution of piperidine (0.5 mL, 5.05 mmol, 1.0 equiv) in 25 mL of THF cooled to −10 °C under argon atmosphere was added triethylamine (0.86 mL, 6.12 mmol, 1.2 equiv), followed by slow addition of bromoacetyl bromide (0.54 mL, 6.12 mmol, 1.2 equiv). The mixture was stirred for 1 h at −10 °C. It was then diluted with 50 mL of ethyl acetate and the formed salts were filtered off before evaporating the solvent under reduced pressure. The crude piperidinyl bromoacetamide was then reacted with 1-Boc-1-methylhydrazine (2.27 mL, 3 equiv) in 2 mL of water, following the protocol for the synthesis of monomer **1a**. The desired compound, isolated in 60% yield (0.822 g, 3.03 mmol), was acetylated in 74% yield (0.697 g, 2.23 mmol) following general procedure **C**. Finally, the Boc group was removed following general procedure **D**, yielding model peptoid **A** in 54% yield (0.256 g, 1.20 mmol).

$R_f$ = 0.17 ($CH_2Cl_2$/MeOH 98:2), $^1H$ NMR (400 MHz, $CDCl_3$): $\delta$ (ppm): 1.56-1.66 (m, 6H, pip), 2.08 (s, 0.28H, Ac *cis* rotamer), 2.25 (s, 2.72H, Ac *trans* rotamer), 2.56 (s, 2.79H, C***$H_3$***NH *trans* rotamer), 2.69 (s, 0.21H, C***$H_3$***NH *cis* rotamer), 3.35-3.55 (m, 4H, pip), 4.42 (s, 2H, NC***$H_2$***C=O).

### c) Segment-coupling reactions

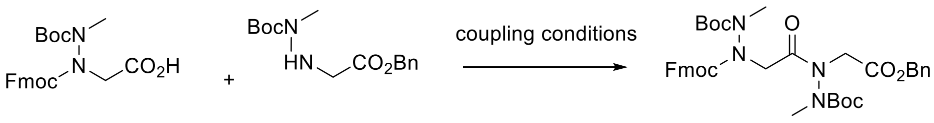


**1c** **1a** **2d**

**Scheme S2.** Evaluation of mixed anhydride methods: (1+1) coupling of acid **1c** and hydrazine **1a.**

**Synthesis of the acid partner 1c from 1a.**

To a solution of monomer **1a** (1.541 g, 5.24 mmol) in dioxane (7 mL) cooled to 0 °C, was added 14 mL of a 10% w/w aqueous solution of $Na_2CO_3$, followed by the slow addition of a solution of FmocCl (1.35 g, 5.23 mmol, 1.0 equiv) in 14 mL of dioxane. Stirring was maintained at 0 °C for 1 h, and for 3 h at room temperature. The desired compound was then extracted with ethyl acetate and the organic layer dried over $MgSO_4$ and evaporated under vacuum. The mixture was purified by flash chromatography on silica gel (cyclohexane/EtOAc 4:1; 2.43 g, 4.71 mmol, 90% yield). For the hydrogenolysis of the benzyl ester, the obtained compound was dissolved in 110 mL of MeOH and 10% Pd/C (118 mg) was added to the solution. Hydrogen was bubbled into the solution until it was saturated ($H_2$ inflated balloon) and the mixture was stirred for 1 h, while maintaining a hydrogen atmosphere above the solution. Pd/C was then filtered off through a pad of celite and the filtrate evaporated under reduced pressure. Pure monomer acid **1c** (1.55 g, 3.64 mmol) was isolated as a colourless solid in 77% yield after purification by chromatography on silica gel (cyclohexane/EtOAc 1:1 to 1:9).

**Isobutyl chloroformate (IBCF)-mediated coupling of monomers 1a and 1c (optimized conditions with 2.1 equivalents of monomer acid 1c).**

To a solution of monomer acid **1c** (200 mg, 0.47 mmol, 2.1 equiv) in DMF (1.75 mL) at −10 °C under argon atmosphere was added *N*-methylmorpholine (NMM) (32 µL, 0.45 mmol, 1.0 equiv) followed by IBCF (58 µL, 0.45 mmol, 2.0 equiv). The reaction mixture was stirred at −10 °C for 10 min, then a solution of monomer **1a** (66 mg, 0.22 mmol, 1 equiv) in DMF (0.56 mL) was added and the temperature was allowed to rise to room temperature. Stirring was maintained for 24 h at room temperature, after which the medium was diluted with water and extracted with ethyl acetate. The organic phase was dried over $MgSO_4$, concentrated under reduced pressure and finally purified on a silica gel column (cyclohexane/EtOAc 4:1), to produce dimer **2d** (121 mg, 0.17 mmol) as a colourless oil in 78% yield.

***N*-Ethoxycarbonyl-2-ethoxy-1,2-dihydroquinoline (EEDQ)-mediated coupling of monomers 1a and 1c (optimized conditions with 1.5 equivalents of monomer acid 1c).**

To a solution of monomer acid **1c** (652 mg, 1.53 mmol, 1.5 equiv) in 1,4-dioxane (1.5 mL) was added monomer **1a** (300 mg, 1.02 mmol, 1.0 equiv) dissolved in 1,4-dioxane (1.0 mL) followed by addition of EEDQ (378 mg, 1.53 mmol, 1.5 equiv) and the mixture was stirred for 24 h at 60 °C. The solution was then diluted with ethyl acetate and successively washed with 1M HCl (3 ×), aq. saturated $NaHCO_3$ (3 ×), water, and brine. The organic layer was dried over $MgSO_4$, filtrated, and concentrated under reduced pressure. The remaining residue was purified on a silica gel column (cyclohexane/EtOAc 4:1), to produce dimer **2d** as a colourless oil (680 mg, 0.67 mmol) in 95% yield.

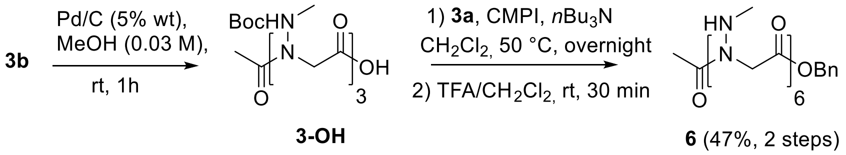


**Scheme S3.** Synthesis of peptoid **6** by a (3+3) segment coupling of trimer Ac-*N*(NBocMe)$_3$-OH and trimer H-N(NBocMe)$_3$-OBn, using Mukaiyama's reagent, 2-chloro-1-methylpyridinium iodide (CMPI).

**Hydrogenolysis of the benzyl ester of trimer 3b.**

To a solution of trimer **3b** (0.60 g, 0.85 mmol) in MeOH (28 mL) was added 10% Pd/C (30 mg, 5 wt %). Hydrogen was bubbled into the solution until it was saturated ($H_2$ inflated balloon) and the mixture was stirred for 30 min, while maintaining a hydrogen atmosphere above the solution. Pd/C was then filtered off through a pad of celite and the filtrate was evaporated under reduced pressure to give the trimer **3-OH** (0.479 g, 0.78 mmol, 92%) as a white solid, used in the coupling reaction without any purification.

**(3+3) Segment coupling of trimers 3-OH and 3a using Mukaiyama's reagent, 2-chloro-1-methylpyridinium iodide.**

To a solution of hydrazine **3a** (66.6 mg, 0.1 mmol, 1.0 equiv), and acid **3-OH** (61.8 mg, 0.1 mmol, 1.0 equiv) in $CH_2Cl_2$ (1 mL) was added 2-chloro-1-methylpyridinium iodide (30.7 mg, 0.12 mmol, 1.2 equiv) under argon atmosphere, followed by the addition of *n*-NBu$_3$ (57 µL, 0.24 mmol, 2.4 equiv). The reaction mixture was heated under reflux for 5 h, after which TLC monitoring indicated complete conversion of the substrates. The solution was cooled, diluted with 10 mL of $CH_2Cl_2$, and successively washed with aq. saturated $NaHCO_3$ (3 ×), water, aq. saturated $NH_4Cl$ (3 ×), and brine. The organic layer was dried over $MgSO_4$, filtered, and concentrated under vacuum. Purification of the crude by flash chromatography on silica gel (cyclohexane/EtOAc 1:1) gave the Boc-protected hexamer as a yellowish oil (90 mg, 0.071 mmol, 71% yield). The Boc groups were removed following general procedure **D**, producing 66% of hexamer **6** as a colourless oil (31 mg, 0.047 mmol) after column chromatography ($SiO_2$, $CH_2Cl_2$/MeOH 96:4).

$R_f$ = 0,34 ($CH_2Cl_2$/MeOH 95:5), HRMS (TOF MS ES$^+$): *m/z* calcd for $C_{27}H_{47}N_{12}O_8$ $[M+H]^+$ 667.3634 found 667.3625. $^1H$ NMR (400 MHz, DMSO-d6): $\delta$ (ppm): 1.86-2.03 (m, 3H, Ac), 2.41-2.47 (m, 18H, 6×C***H***$_3$NH), 4.18-4.27 (m, 2H, C***H***$_2$CO$_2$Bn), 4.42-4.48 (m, 10H, 5×NC***H***$_2$C=O), 4.63-4.96 (m, 6H, 6×NH), 5.14-5.19 (m, 2H, C***H***$_2$Ph), 7.34-7.36 (m, 5H, $C_6H_5$).

## 3. X-ray crystallographic data for peptoid 2

### Crystal structure report for dimer 2 (Ac-*N*NMe-*N*NMe-OBn)

Crystals of dimer **2** suitable for X-ray crystallography were grown by slow evaporation from chloroform solution.

A specimen of $C_{15}H_{22}N_4O_4$, approximate dimensions 0.134 mm × 0.142 mm × 0.624 mm, was used for the X-ray crystallographic analysis. The X-ray intensity data were measured (λ = 0.71073 Å).

The total exposure time was 8.83 hours. The frames were integrated with the Bruker SAINT software package using a narrow-frame algorithm. The integration of the data using an orthorhombic unit cell yielded a total of 122188 reflections to a maximum θ angle of 30.51° (0.70 Å resolution), of which 5015 were independent (average redundancy 24.365, completeness = 99.9%, $R_{int}$ = 7.96%, $R_{sig}$ = 3.12%) and 3393 (67.66%) were greater than $2\sigma(F^2)$. The final cell constants of a = 10.3108(12) Å, b = 11.2142(12) Å, c = 28.442(3) Å, volume = 3288.7(6) $Å^3$, are based upon the refinement of the XYZ-centroids of 523 reflections above 20 σ(I) with 4.921° < 2θ < 56.00°. Data were corrected for absorption effects using the Numerical Mu From Formula method (SADABS). The ratio of minimum to maximum apparent transmission was 0.920. The calculated minimum and maximum transmission coefficients (based on crystal size) are 0.9430 and 0.9870.

The structure was solved and refined using the Bruker SHELXTL Software Package, using the space group *Pbca*, with Z = 8 for the formula unit, $C_{15}H_{22}N_4O_4$. The final anisotropic full-matrix least-squares refinement on $F^2$ with 219 variables converged at R1 = 4.10%, for the observed data and wR2 = 10.42% for all data. The goodness-of-fit was 1.005. The largest peak in the final difference electron density synthesis was 0.230 $e^-/Å^3$ and the largest hole was −0.222 $e^-/Å^3$ with an RMS deviation of 0.045 $e^-/Å^3$. On the basis of the final model, the calculated density was 1.302 $g/cm^3$ and F(000), 1376 $e^-$.

***Thermal ellipsoid plot at 50% probability levels for dimer 2***

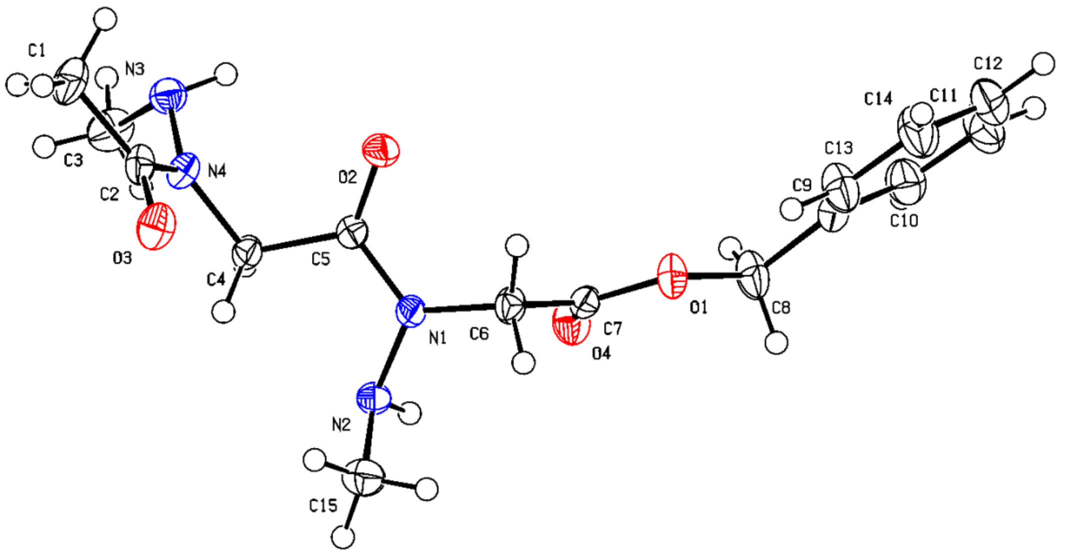

## Sample and crystal data for dimer 2.

| | | |
|---|---|---|
| **Identification code** | Dimer 2 | |
| **Chemical formula** | $C_{15}H_{22}N_4O_4$ | |
| **Formula weight** | 322.36 g/mol | |
| **Temperature** | 173(2) K | |
| **Wavelength** | 0.71073 Å | |
| **Crystal size** | 0.134 x 0.142 x 0.624 mm | |
| **Crystal system** | orthorhombic | |
| **Space group** | P b c a | |
| **Unit cell dimensions** | a = 10.3108(12) Å | α = 90° |
| | b = 11.2142(12) Å | β = 90° |
| | c = 28.442(3) Å | γ = 90° |
| **Volume** | 3288.7(6) $Å^3$ | |
| **Z** | 8 | |
| **Density (calculated)** | 1.302 $g/cm^3$ | |
| **Absorption coefficient** | 0.096 $mm^{-1}$ | |
| **F(000)** | 1376 | |

## Data collection and structure refinement for dimer 2.

| | | |
|---|---|---|
| **Theta range for data collection** | 2.78 to 30.51° | |
| **Index ranges** | -14<=h<=14, -15<=k<=15, -40<=l<=40 | |
| **Reflections collected** | 122188 | |
| **Independent reflections** | 5015 [R(int) = 0.0796] | |
| **Coverage of independent reflections** | 99.9% | |
| **Absorption correction** | Numerical Mu From Formula | |
| **Max. and min. transmission** | 0.9870 and 0.9430 | |
| **Structure solution technique** | direct methods | |
| **Structure solution program** | SHELXT 2018/2 (Sheldrick, 2018) | |
| **Refinement method** | Full-matrix least-squares on $F^2$ | |
| **Refinement program** | SHELXL-2018/3 (Sheldrick, 2018) | |
| **Function minimized** | $\Sigma$ w($F_o^2$ - $F_c^2$)$^2$ | |
| **Data / restraints / parameters** | 5015 / 0 / 219 | |
| **Goodness-of-fit on $F^2$** | 1.005 | |
| **$\Delta/\sigma_{max}$** | 0.001 | |
| **Final R indices** | 3393 data; I>2σ(I) | R1 = 0.0410, wR2 = 0.0889 |
| | all data | R1 = 0.0753, wR2 = 0.1042 |
| **Weighting scheme** | w=1/[$\sigma^2$($F_o^2$)+(0.0396P)$^2$+1.1109P]<br>where P=($F_o^2$+2$F_c^2$)/3 | |
| **Largest diff. peak and hole** | 0.230 and -0.222 e$Å^{-3}$ | |
| **R.M.S. deviation from mean** | 0.045 e$Å^{-3}$ | |

## Atomic coordinates and equivalent isotropic atomic displacement parameters (Å$^2$) for Dimer 2.

U(eq) is defined as one third of the trace of the orthogonalized $U_{ij}$ tensor.

| | x/a | y/b | z/c | U(eq) |
|---|---|---|---|---|
| O1 | 0.23723(9) | 0.37377(8) | 0.64455(3) | 0.0274(2) |
| O2 | 0.34197(8) | 0.53731(7) | 0.51078(3) | 0.02509(19) |
| O3 | 0.16219(9) | 0.62881(8) | 0.40681(3) | 0.0322(2) |
| O4 | 0.28570(9) | 0.23785(8) | 0.58921(3) | 0.0308(2) |
| N1 | 0.19208(10) | 0.39190(9) | 0.51903(3) | 0.0215(2) |
| N2 | 0.13331(10) | 0.29388(9) | 0.49668(4) | 0.0226(2) |
| N3 | 0.47836(10) | 0.51133(10) | 0.40615(4) | 0.0258(2) |
| C1 | 0.35167(15) | 0.70303(12) | 0.36809(5) | 0.0329(3) |
| C2 | 0.27802(12) | 0.61597(11) | 0.39809(4) | 0.0243(3) |
| N4 | 0.34383(10) | 0.52029(9) | 0.41434(3) | 0.0229(2) |
| C4 | 0.27706(12) | 0.42942(10) | 0.44125(4) | 0.0224(2) |
| C5 | 0.27471(11) | 0.45849(10) | 0.49340(4) | 0.0189(2) |
| C6 | 0.16960(12) | 0.41735(11) | 0.56840(4) | 0.0234(2) |
| C7 | 0.23911(12) | 0.33153(11) | 0.60033(4) | 0.0217(2) |
| C8 | 0.29476(15) | 0.29743(12) | 0.67966(4) | 0.0334(3) |
| C9 | 0.32829(12) | 0.37030(12) | 0.72216(4) | 0.0270(3) |
| C10 | 0.41336(14) | 0.32179(13) | 0.75465(5) | 0.0356(3) |
| C11 | 0.44338(16) | 0.38345(16) | 0.79525(5) | 0.0460(4) |
| C12 | 0.39198(16) | 0.49446(15) | 0.80348(5) | 0.0434(4) |
| C13 | 0.27565(15) | 0.48148(13) | 0.73088(5) | 0.0370(3) |
| C14 | 0.30825(17) | 0.54396(14) | 0.77135(5) | 0.0433(4) |
| C15 | 0.99346(13) | 0.30385(13) | 0.49262(5) | 0.0349(3) |
| C3 | 0.50929(14) | 0.41495(13) | 0.37358(5) | 0.0357(3) |

## Bond lengths (Å) for dimer 2.

| | | | |
|---|---|---|---|
| O1-C7 | 1.3441(14) | O1-C8 | 1.4428(15) |
| O2-C5 | 1.2275(13) | O3-C2 | 1.2282(16) |
| O4-C7 | 1.1977(15) | N1-C5 | 1.3472(15) |
| N1-N2 | 1.4071(13) | N1-C6 | 1.4513(15) |
| N2-C15 | 1.4509(17) | N2-H10 | 0.874(16) |
| N3-N4 | 1.4102(14) | N3-C3 | 1.4588(17) |
| N3-H11 | 0.887(16) | C1-C2 | 1.5027(17) |
| C1-H14 | 0.98 | C1-H13 | 0.98 |
| C1-H1 | 0.98 | C2-N4 | 1.3511(15) |
| N4-C4 | 1.4484(15) | C4-C5 | 1.5189(16) |
| C4-H4A | 0.99 | C4-H4B | 0.99 |
| C6-C7 | 1.5050(17) | C6-H21 | 0.99 |
| C6-H20 | 0.99 | C8-C9 | 1.4996(18) |
| C8-H5 | 0.99 | C8-H6 | 0.99 |
| C9-C13 | 1.3822(19) | C9-C10 | 1.3854(18) |
| C10-C11 | 1.381(2) | C10-H22 | 0.95 |
| C11-C12 | 1.373(2) | C11-H23 | 0.95 |

| | | | |
|---|---|---|---|
| C12-C14 | 1.374(2) | C12-H2 | 0.95 |
| C13-C14 | 1.389(2) | C13-H3 | 0.95 |
| C14-H4 | 0.95 | C15-H8 | 0.98 |
| C15-H7 | 0.98 | C15-H9 | 0.98 |
| C3-H17 | 0.98 | C3-H16 | 0.98 |
| C3-H15 | 0.98 | | |

**Bond angles (°) for dimer 2.**

| | | | |
|---|---|---|---|
| C7-O1-C8 | 115.63(10) | C5-N1-N2 | 117.44(9) |
| C5-N1-C6 | 121.03(10) | N2-N1-C6 | 121.47(9) |
| N1-N2-C15 | 113.81(10) | N1-N2-H10 | 108.0(10) |
| C15-N2-H10 | 113.9(10) | N4-N3-C3 | 111.89(10) |
| N4-N3-H11 | 108.8(10) | C3-N3-H11 | 110.1(10) |
| C2-C1-H14 | 109.5 | C2-C1-H13 | 109.5 |
| H14-C1-H13 | 109.5 | C2-C1-H1 | 109.5 |
| H14-C1-H1 | 109.5 | H13-C1-H1 | 109.5 |
| O3-C2-N4 | 120.82(11) | O3-C2-C1 | 122.00(12) |
| N4-C2-C1 | 117.15(11) | C2-N4-N3 | 119.61(10) |
| C2-N4-C4 | 120.05(10) | N3-N4-C4 | 120.32(10) |
| N4-C4-C5 | 111.87(10) | N4-C4-H4A | 109.2 |
| C5-C4-H4A | 109.2 | N4-C4-H4B | 109.2 |
| C5-C4-H4B | 109.2 | H4A-C4-H4B | 107.9 |
| O2-C5-N1 | 122.57(10) | O2-C5-C4 | 122.61(10) |
| N1-C5-C4 | 114.82(10) | N1-C6-C7 | 112.46(10) |
| N1-C6-H21 | 109.1 | C7-C6-H21 | 109.1 |
| N1-C6-H20 | 109.1 | C7-C6-H20 | 109.1 |
| H21-C6-H20 | 107.8 | O4-C7-O1 | 124.20(11) |
| O4-C7-C6 | 126.34(11) | O1-C7-C6 | 109.42(10) |
| O1-C8-C9 | 109.23(11) | O1-C8-H5 | 109.8 |
| C9-C8-H5 | 109.8 | O1-C8-H6 | 109.8 |
| C9-C8-H6 | 109.8 | H5-C8-H6 | 108.3 |
| C13-C9-C10 | 118.89(12) | C13-C9-C8 | 123.07(12) |
| C10-C9-C8 | 118.01(12) | C11-C10-C9 | 120.20(14) |
| C11-C10-H22 | 119.9 | C9-C10-H22 | 119.9 |
| C12-C11-C10 | 120.66(14) | C12-C11-H23 | 119.7 |
| C10-C11-H23 | 119.7 | C11-C12-C14 | 119.69(14) |
| C11-C12-H2 | 120.2 | C14-C12-H2 | 120.2 |
| C9-C13-C14 | 120.58(13) | C9-C13-H3 | 119.7 |
| C14-C13-H3 | 119.7 | C12-C14-C13 | 119.96(15) |
| C12-C14-H4 | 120.0 | C13-C14-H4 | 120.0 |
| N2-C15-H8 | 109.5 | N2-C15-H7 | 109.5 |
| H8-C15-H7 | 109.5 | N2-C15-H9 | 109.5 |
| H8-C15-H9 | 109.5 | H7-C15-H9 | 109.5 |
| N3-C3-H17 | 109.5 | N3-C3-H16 | 109.5 |
| H17-C3-H16 | 109.5 | N3-C3-H15 | 109.5 |
| H17-C3-H15 | 109.5 | H16-C3-H15 | 109.5 |

## Torsion angles (°) for dimer 2.

| | | | |
|---|---|---|---|
| C5-N1-N2-C15 | -116.06(12) | C6-N1-N2-C15 | 66.78(14) |
| O3-C2-N4-N3 | -176.29(11) | C1-C2-N4-N3 | 5.36(16) |
| O3-C2-N4-C4 | 1.83(17) | C1-C2-N4-C4 | -176.53(11) |
| C3-N3-N4-C2 | -111.59(13) | C3-N3-N4-C4 | 70.30(14) |
| C2-N4-C4-C5 | -87.79(13) | N3-N4-C4-C5 | 90.31(13) |
| N2-N1-C5-O2 | -171.57(10) | C6-N1-C5-O2 | 5.60(17) |
| N2-N1-C5-C4 | 9.09(15) | C6-N1-C5-C4 | -173.73(10) |
| N4-C4-C5-O2 | -12.53(16) | N4-C4-C5-N1 | 166.80(10) |
| C5-N1-C6-C7 | -103.36(13) | N2-N1-C6-C7 | 73.70(14) |
| C8-O1-C7-O4 | -0.79(18) | C8-O1-C7-C6 | 176.91(11) |
| N1-C6-C7-O4 | -15.19(17) | N1-C6-C7-O1 | 167.17(10) |
| C7-O1-C8-C9 | 160.12(11) | O1-C8-C9-C13 | 18.09(19) |
| O1-C8-C9-C10 | -163.74(12) | C13-C9-C10-C11 | 0.7(2) |
| C8-C9-C10-C11 | -177.50(14) | C9-C10-C11-C12 | -1.5(2) |
| C10-C11-C12-C14 | 1.0(3) | C10-C9-C13-C14 | 0.5(2) |
| C8-C9-C13-C14 | 178.67(14) | C11-C12-C14-C13 | 0.3(3) |
| C9-C13-C14-C12 | -1.0(2) | | |

## Anisotropic atomic displacement parameters (Å$^2$) for dimer 2.

The anisotropic atomic displacement factor exponent takes the form: $-2\pi^2[ h^2 a^{*2} U_{11} + ... + 2 h k a^* b^* U_{12} ]$

| | $U_{11}$ | $U_{22}$ | $U_{33}$ | $U_{23}$ | $U_{13}$ | $U_{12}$ |
|---|---|---|---|---|---|---|
| O1 | 0.0384(5) | 0.0273(5) | 0.0166(4) | 0.0004(3) | -0.0003(4) | 0.0053(4) |
| O2 | 0.0285(4) | 0.0207(4) | 0.0261(4) | -0.0007(3) | -0.0024(3) | -0.0045(4) |
| O3 | 0.0331(5) | 0.0342(5) | 0.0293(5) | 0.0031(4) | 0.0054(4) | 0.0084(4) |
| O4 | 0.0397(5) | 0.0260(5) | 0.0265(5) | -0.0028(4) | 0.0002(4) | 0.0047(4) |
| N1 | 0.0286(5) | 0.0192(5) | 0.0167(4) | -0.0013(4) | 0.0006(4) | -0.0055(4) |
| N2 | 0.0288(5) | 0.0165(5) | 0.0225(5) | -0.0008(4) | -0.0008(4) | -0.0045(4) |
| N3 | 0.0243(5) | 0.0262(5) | 0.0268(5) | 0.0016(4) | -0.0003(4) | -0.0007(4) |
| C1 | 0.0467(8) | 0.0252(6) | 0.0268(6) | 0.0086(5) | 0.0067(6) | 0.0033(6) |
| C2 | 0.0337(7) | 0.0226(6) | 0.0166(5) | -0.0001(4) | 0.0021(5) | 0.0025(5) |
| N4 | 0.0252(5) | 0.0220(5) | 0.0216(5) | 0.0050(4) | 0.0036(4) | 0.0000(4) |
| C4 | 0.0290(6) | 0.0201(5) | 0.0181(5) | 0.0020(4) | 0.0009(5) | -0.0035(5) |
| C5 | 0.0220(5) | 0.0147(5) | 0.0199(5) | 0.0018(4) | -0.0014(4) | 0.0019(4) |
| C6 | 0.0297(6) | 0.0226(6) | 0.0177(5) | -0.0017(4) | 0.0032(5) | 0.0000(5) |
| C7 | 0.0246(6) | 0.0225(6) | 0.0181(5) | -0.0001(4) | 0.0033(4) | -0.0040(5) |
| C8 | 0.0489(8) | 0.0296(7) | 0.0218(6) | 0.0022(5) | -0.0080(6) | 0.0057(6) |
| C9 | 0.0306(6) | 0.0310(7) | 0.0196(5) | 0.0037(5) | 0.0002(5) | -0.0032(5) |
| C10 | 0.0352(7) | 0.0396(8) | 0.0322(7) | 0.0017(6) | -0.0049(6) | 0.0048(6) |
| C11 | 0.0448(9) | 0.0593(10) | 0.0338(8) | 0.0025(7) | -0.0159(7) | 0.0022(8) |
| C12 | 0.0548(10) | 0.0486(9) | 0.0267(7) | -0.0044(6) | -0.0082(7) | -0.0093(8) |
| C13 | 0.0517(9) | 0.0325(7) | 0.0269(7) | 0.0001(6) | -0.0096(6) | 0.0045(7) |
| C14 | 0.0644(10) | 0.0339(8) | 0.0316(7) | -0.0054(6) | -0.0062(7) | 0.0014(7) |

| | $U_{11}$ | $U_{22}$ | $U_{33}$ | $U_{23}$ | $U_{13}$ | $U_{12}$ |
|---|---|---|---|---|---|---|
| C15 | 0.0293(7) | 0.0395(8) | 0.0361(8) | -0.0024(6) | -0.0006(6) | -0.0068(6) |
| C3 | 0.0332(7) | 0.0317(7) | 0.0423(8) | -0.0036(6) | 0.0092(6) | 0.0027(6) |

**Hydrogen atomic coordinates and isotropic atomic displacement parameters (Å$^2$) for Dimer 2.**

| | x/a | y/b | z/c | U(eq) |
|---|---|---|---|---|
| H10 | 0.1592(14) | 0.2291(14) | 0.5109(5) | 0.034(4) |
| H11 | 0.5182(15) | 0.4997(14) | 0.4334(6) | 0.036(4) |
| H14 | 0.2933 | 0.7669 | 0.3578 | 0.049 |
| H13 | 0.3866 | 0.6617 | 0.3405 | 0.049 |
| H1 | 0.4231 | 0.7373 | 0.3864 | 0.049 |
| H4A | 0.3209 | 0.3518 | 0.4365 | 0.027 |
| H4B | 0.1869 | 0.4219 | 0.4296 | 0.027 |
| H21 | 0.0753 | 0.4134 | 0.5748 | 0.028 |
| H20 | 0.1992 | 0.4995 | 0.5754 | 0.028 |
| H5 | 0.3740 | 0.2596 | 0.6669 | 0.04 |
| H6 | 0.2330 | 0.2336 | 0.6884 | 0.04 |
| H22 | 0.4512 | 0.2459 | 0.7490 | 0.043 |
| H23 | 0.5002 | 0.3487 | 0.8177 | 0.055 |
| H2 | 0.4142 | 0.5369 | 0.8313 | 0.052 |
| H3 | 0.2166 | 0.5154 | 0.7090 | 0.044 |
| H4 | 0.2727 | 0.6209 | 0.7768 | 0.052 |
| H8 | -0.0442 | 0.3170 | 0.5238 | 0.052 |
| H7 | -0.0417 | 0.2301 | 0.4792 | 0.052 |
| H9 | -0.0282 | 0.3711 | 0.4721 | 0.052 |
| H17 | 0.4719 | 0.3402 | 0.3852 | 0.054 |
| H16 | 0.6037 | 0.4066 | 0.3711 | 0.054 |
| H15 | 0.4731 | 0.4333 | 0.3425 | 0.054 |

## 4. Analytical HPLC chromatograms of peptoids 1–6 and monomer A

Purity of each final product was confirmed by analytical RP-HPLC using a 150 × 4.6 mm Nucleodur C18 column (Macherey-Nagel) (3 µm, 4.6 mm × 125 mm, 110 Å) and a gradient of 5–95% of ACN in water (0.1% TFA) over 7 min with a flow rate of 1.0 mL/min. Absorbance signal at 214 nm.

| peptoid | retention time (min) | Purity (%) |
|---|---|---|
| 1 | 8.18 | 100 |
| 2 | 7.71 | 99 |
| 3 | 7.57 | 98 |
| 4 | 7.55 | 93 |
| 5 | 7.55 | 92 |
| 6 | 7.84 | 98 |

### Compound 1

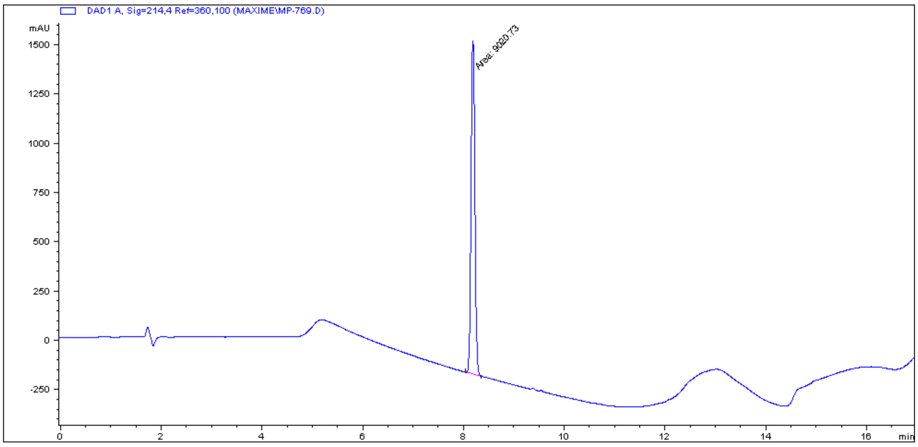


Signal 1: DAD1 A, Sig=214,4 Ref=360,100

| Peak # | RetTime [min] | Type | Width [min] | Area [mAU*s] | Height [mAU] | Area % |
|---|---|---|---|---|---|---|
| 1 | 8.181 | MM | 0.0887 | 9014.04395 | 1693.61914 | 100.0000 |

### Compound 2

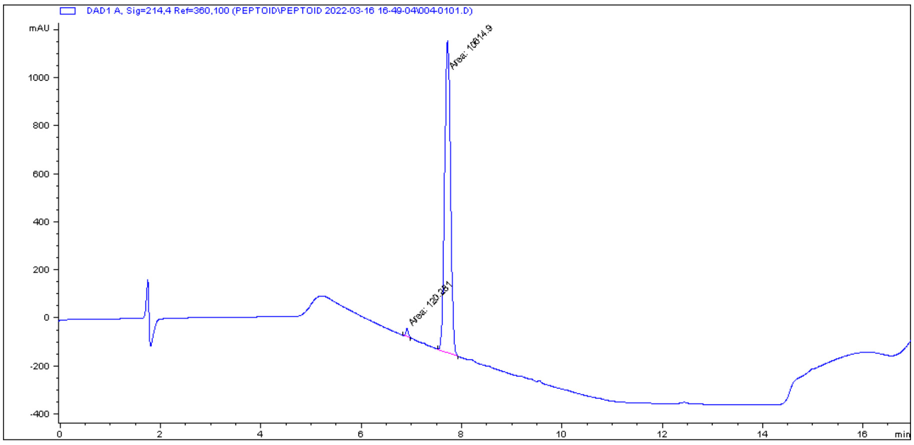

Signal 1: DAD1 A, Sig=214,4 Ref=360,100

| Peak # | RetTime [min] | Type | Width [min] | Area [mAU*s] | Height [mAU] | Area % |
|---|---|---|---|---|---|---|
| 1 | 6.905 | MM | 0.0606 | 130.27127 | 35.82507 | 1.2112 |
| 2 | 7.713 | MM | 0.1363 | 1.06252e4 | 1299.44299 | 98.7888 |

**Compound 3**

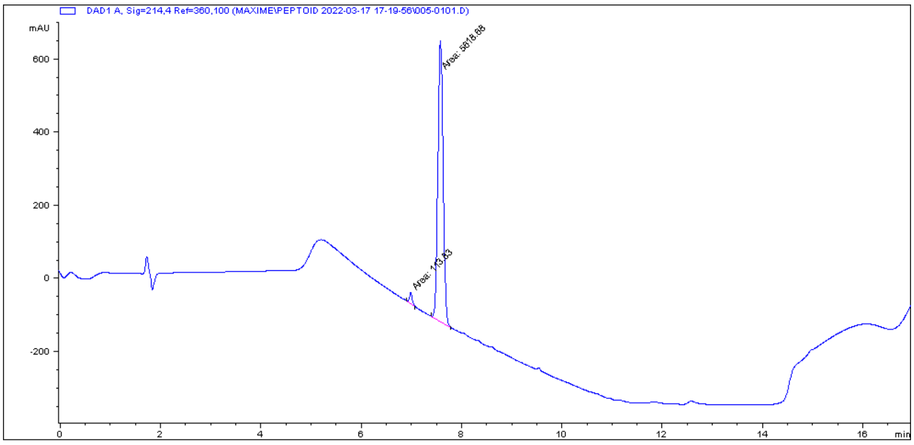


Signal 1: DAD1 A, Sig=214,4 Ref=360,100

| Peak # | RetTime [min] | Type | Width [min] | Area [mAU*s] | Height [mAU] | Area % |
|---|---|---|---|---|---|---|
| 1 | 6.979 | MM | 0.0655 | 128.82013 | 32.78517 | 2.2325 |
| 2 | 7.571 | MM | 0.1222 | 5641.36621 | 769.24115 | 97.7675 |

**Compound 4**

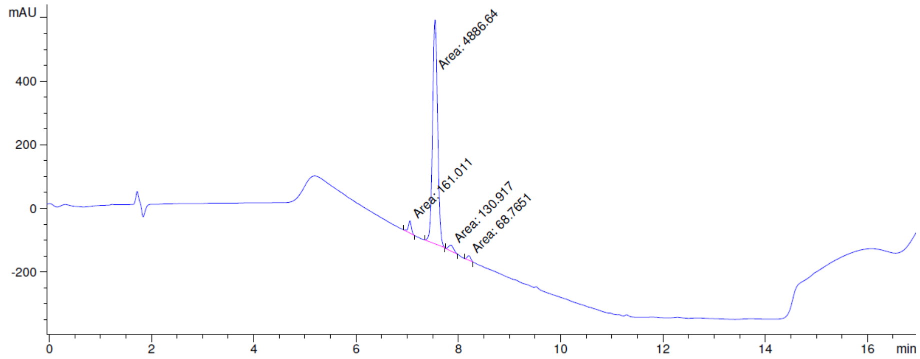


Signal 1: DAD1 A, Sig=214,4 Ref=360,100

| Peak # | RetTime [min] | Type | Width [min] | Area [mAU*s] | Height [mAU] | Area % |
|---|---|---|---|---|---|---|
| 1 | 7.056 | MM | 0.0689 | 161.01077 | 38.94917 | 3.0684 |
| 2 | 7.546 | MM | 0.1153 | 4886.63672 | 706.31873 | 93.1262 |
| 3 | 7.862 | MM | 0.1166 | 130.91669 | 18.71139 | 2.4949 |
| 4 | 8.209 | MM | 0.0774 | 68.76509 | 14.80008 | 1.3105 |

**Compound 5**

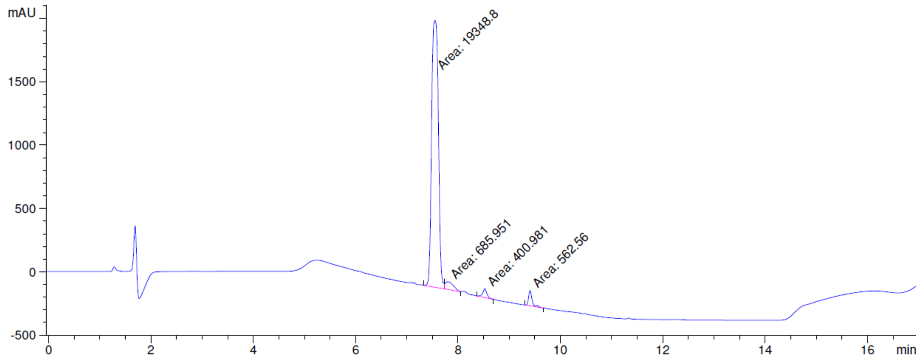


Signal 1: DAD1 A, Sig=214,4 Ref=360,100

| Peak # | RetTime [min] | Type | Width [min] | Area [mAU*s] | Height [mAU] | Area % |
|---|---|---|---|---|---|---|
| 1 | 7.550 | MF | 0.1530 | 1.93488e4 | 2108.26660 | 92.1447 |
| 2 | 7.807 | FM | 0.1862 | 685.95123 | 61.41019 | 3.2667 |
| 3 | 8.527 | MM | 0.0966 | 400.98114 | 69.19804 | 1.9096 |
| 4 | 9.408 | MM | 0.0773 | 562.56024 | 121.30586 | 2.6791 |

**Compound 6**

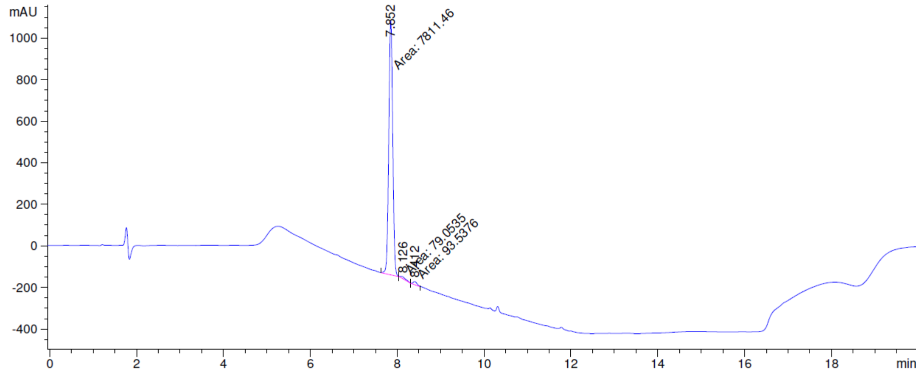


Signal 1: DAD1 A, Sig=214,4 Ref=360,100

| Peak # | RetTime [min] | Type | Width [min] | Area [mAU*s] | Height [mAU] | Area % |
|---|---|---|---|---|---|---|
| 1 | 7.852 | MM | 0.1063 | 7811.45703 | 1224.27087 | 97.8383 |
| 2 | 8.126 | MM | 0.1506 | 79.05353 | 8.75064 | 0.9901 |
| 3 | 8.412 | MM | 0.1017 | 93.53758 | 15.32347 | 1.1716 |

**Monomer A**

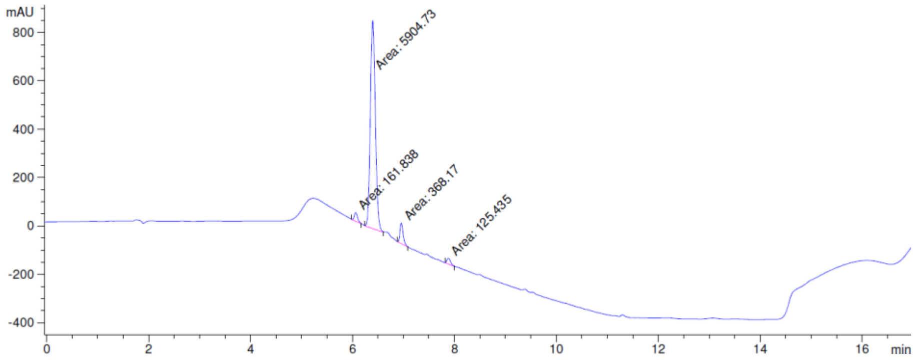


Signal 1: DAD1 A, Sig=214,4 Ref=360,100

| Peak # | RetTime [min] | Type | Width [min] | Area [mAU*s] | Height [mAU] | Area % |
|---|---|---|---|---|---|---|
| 1 | 6.063 | MM | 0.0747 | 161.83788 | 36.13095 | 2.4670 |
| 2 | 6.397 | MM | 0.1142 | 5904.72949 | 861.72980 | 90.0087 |
| 3 | 6.957 | MM | 0.0715 | 368.17004 | 85.81860 | 5.6122 |
| 4 | 7.883 | MM | 0.0844 | 125.43545 | 24.76782 | 1.9121 |

## 5. NMR spectra

**Monomer 1 (Ac-*N*NMe-OBn)**

$^{1}$H NMR, $CDCl_3$, 8 mM

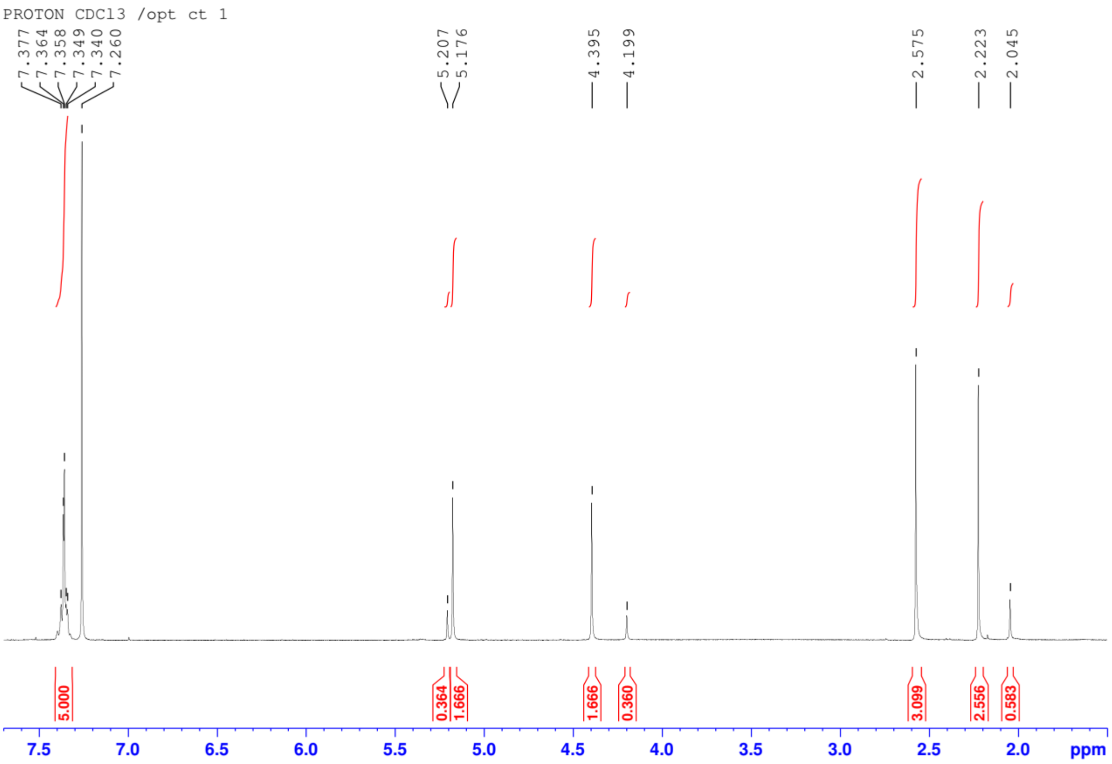

$^{1}H$ NMR, $CD_3CN$, 8 mM

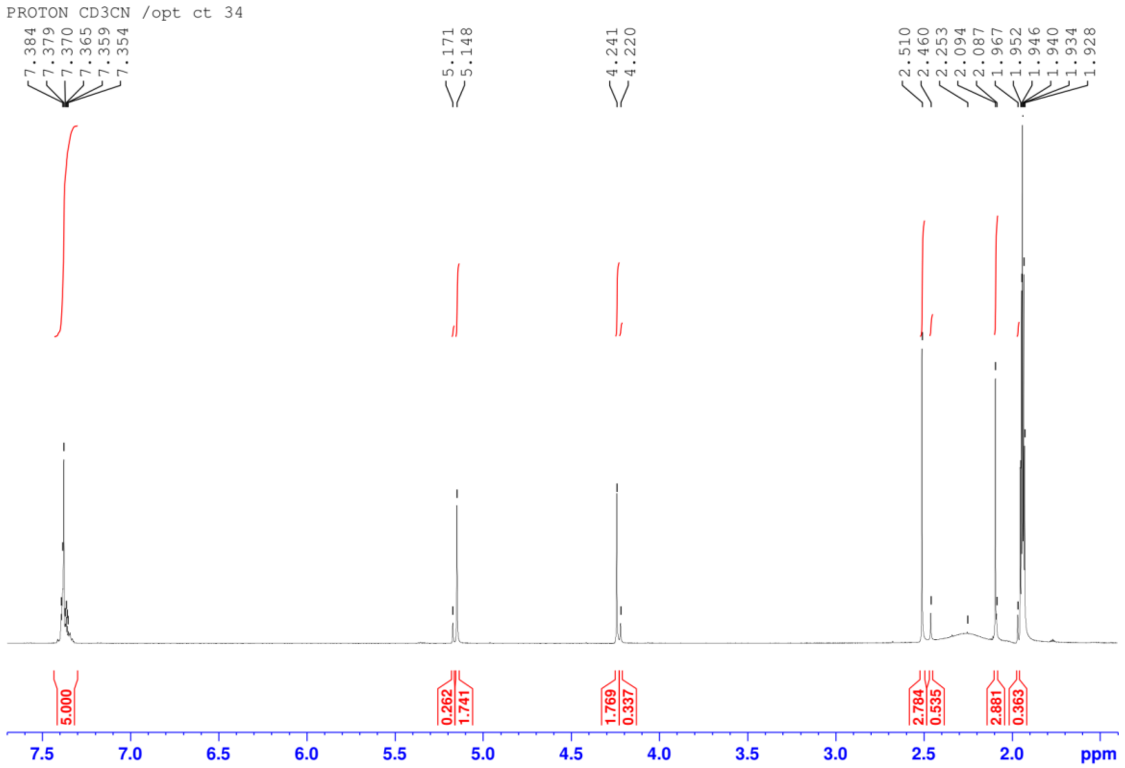


$^{1}H$ NMR, $C_6D_6$, 8 mM

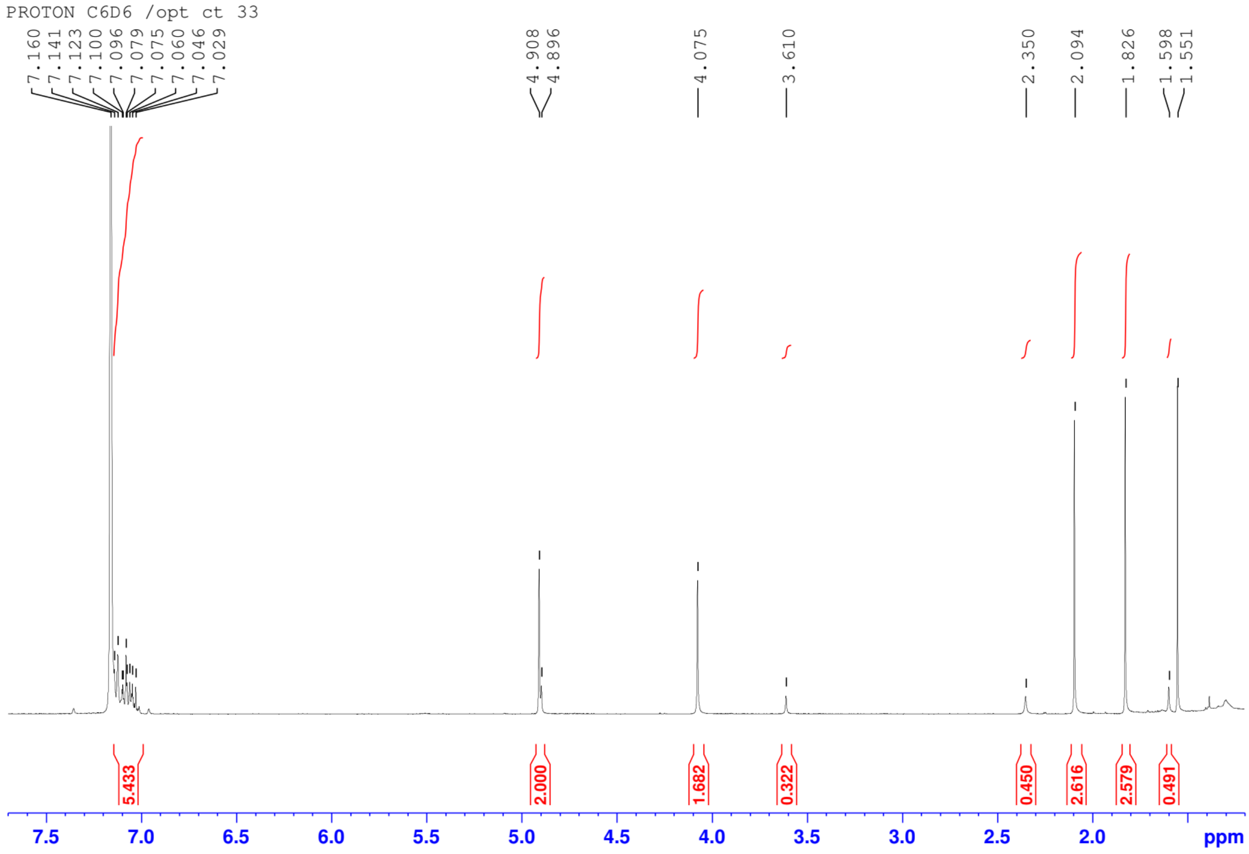

$^1$H NMR, DMSO-$d_6$, 8 mM

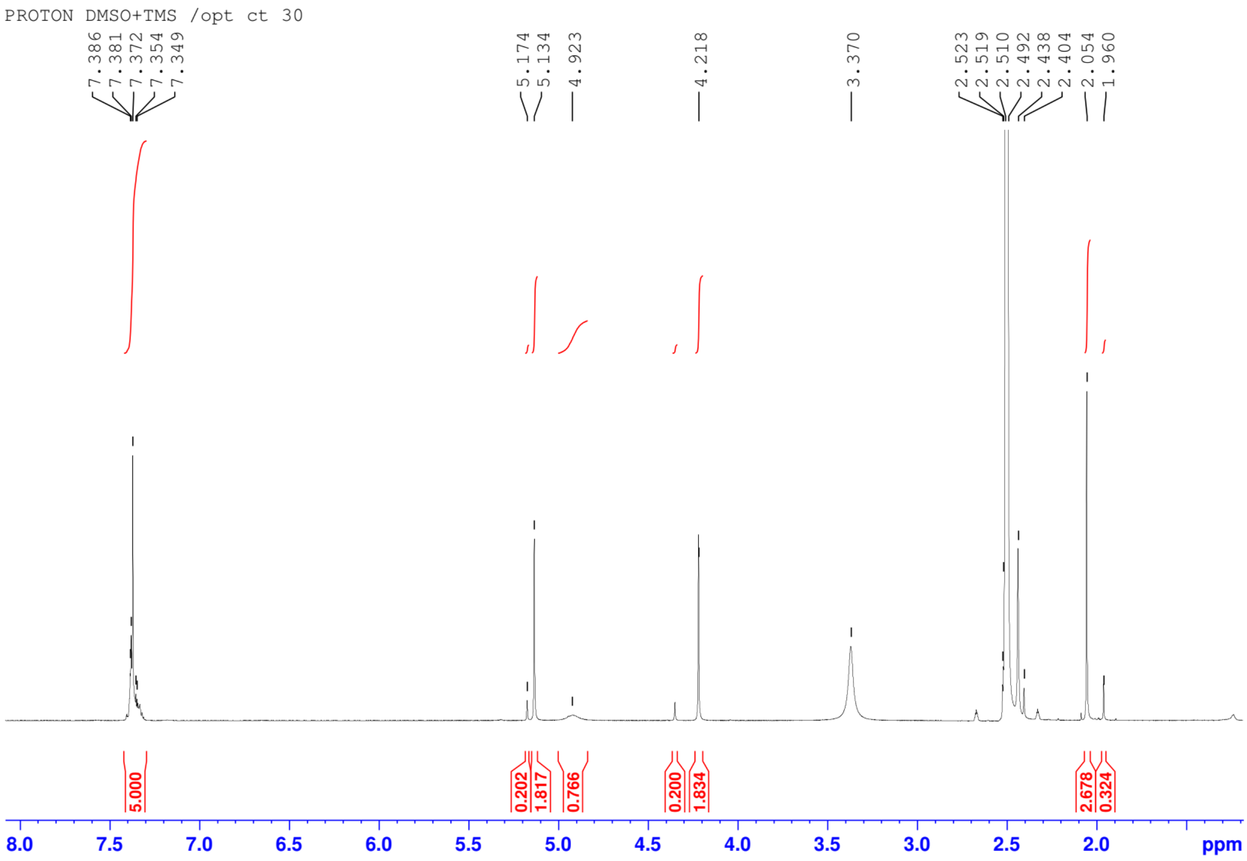


$^1$H NMR, $CD_3OD$, 8 mM

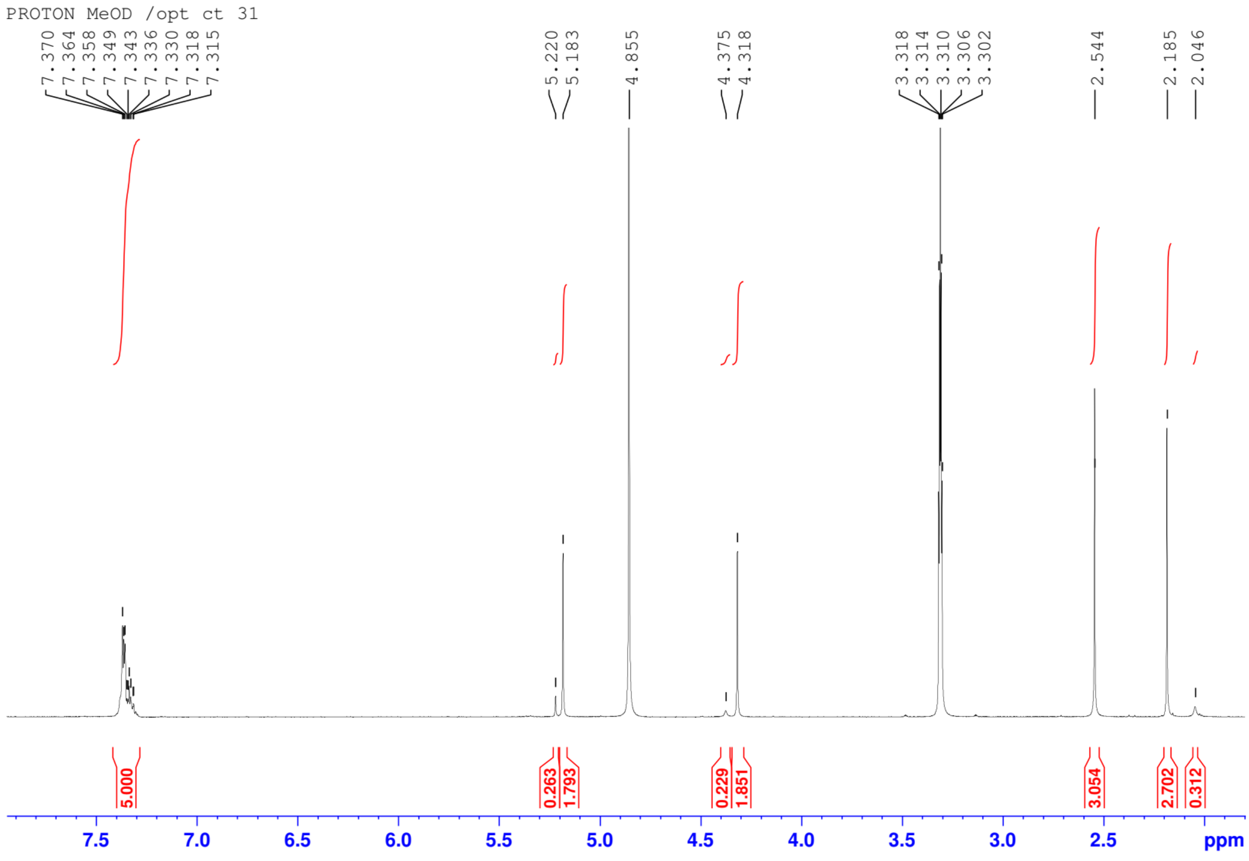

$^1H$ NMR, $D_2O$, 8 mM

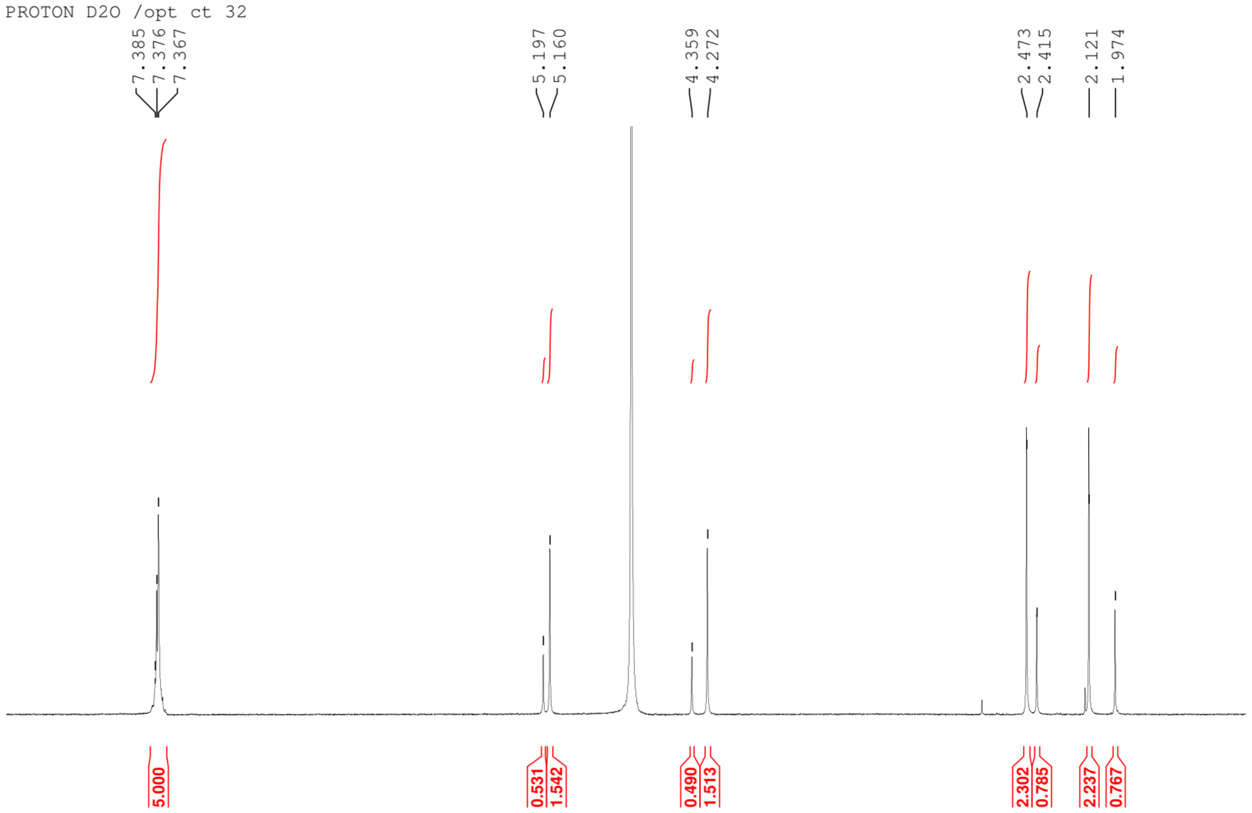


$^{13}C$ NMR, $CDCl_3$

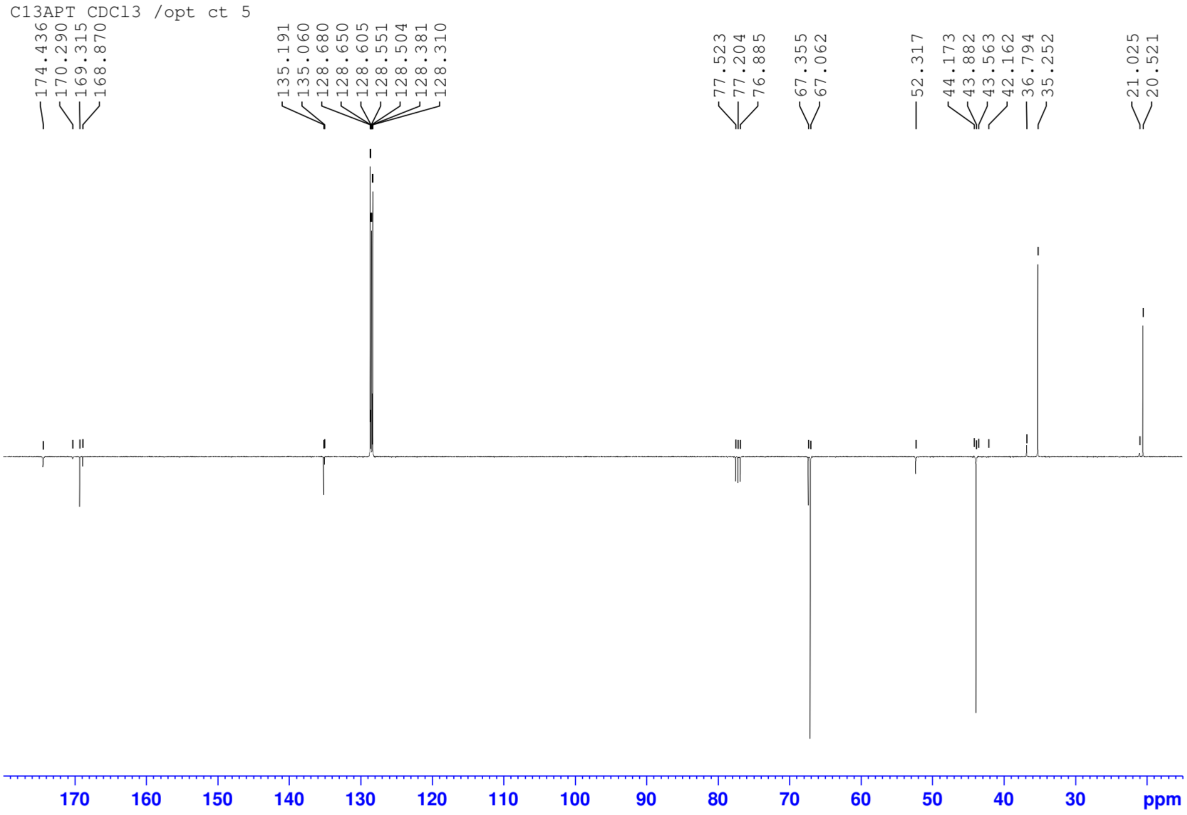

2D HSQC NMR, $CDCl_3$, 5 mM

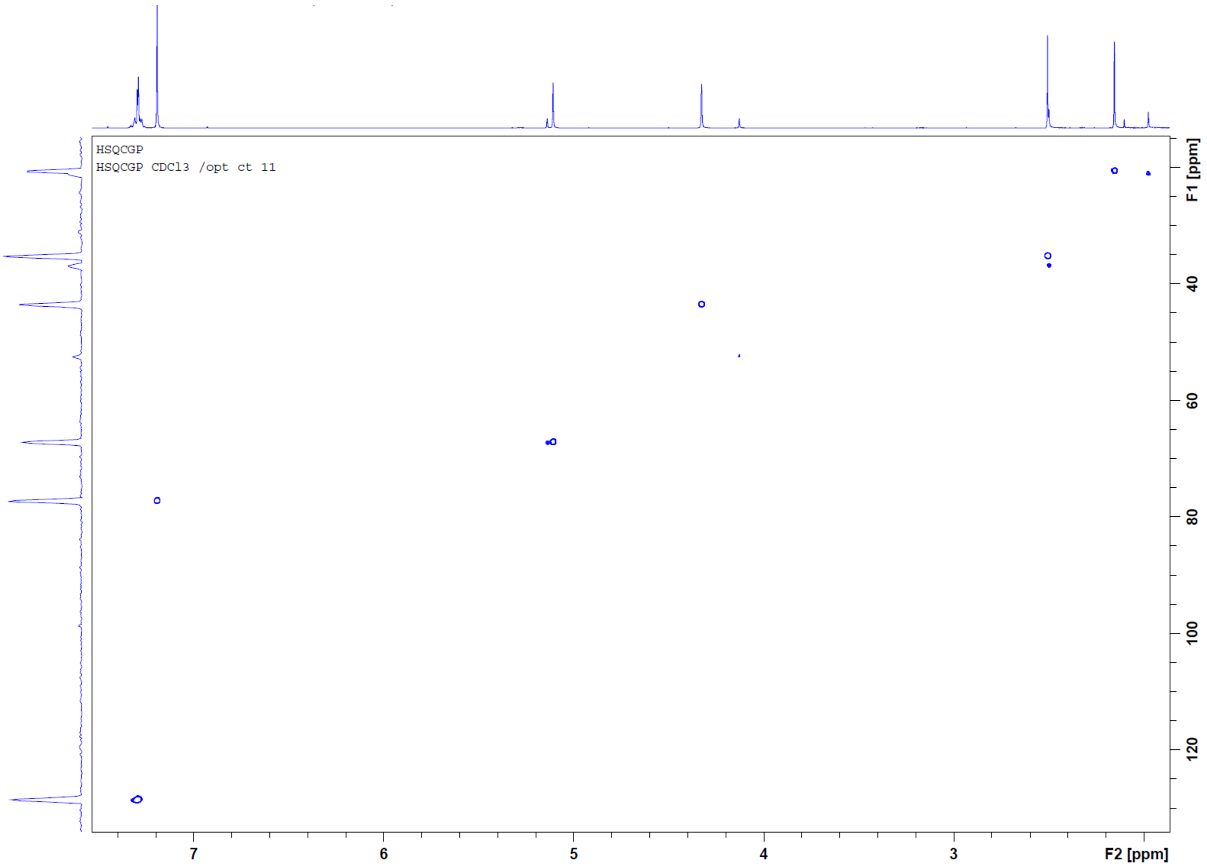


2D HMBC NMR, $CDCl_3$, 5 mM

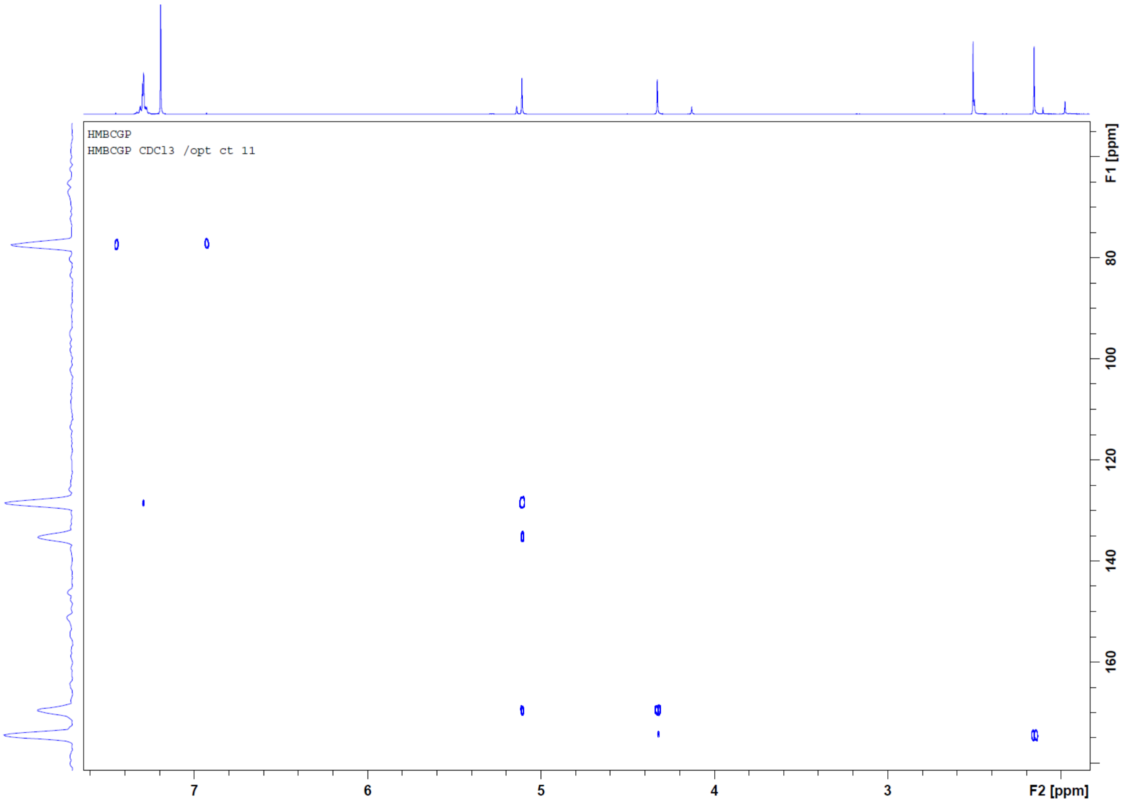

Portions of 2D NOESY spectrum of peptoid **1** in DMSO-$d_6$ showing correlations between protons H2 and H4 and H3 and H4 in the predominant trans-conformation and between protons H3 and H2 in the minor cis-conformation

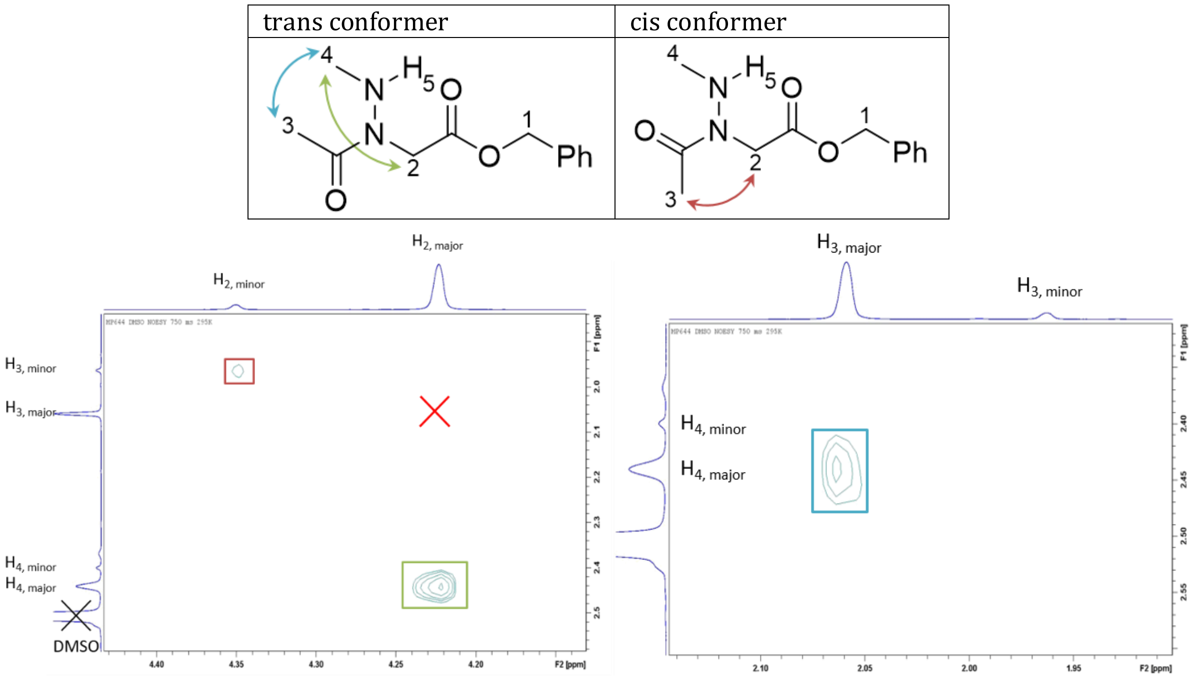


## Dimer 2 (Ac-(*N*NMe)$_2$-OBn)

$^1$H NMR, $CDCl_3$, 8 mM

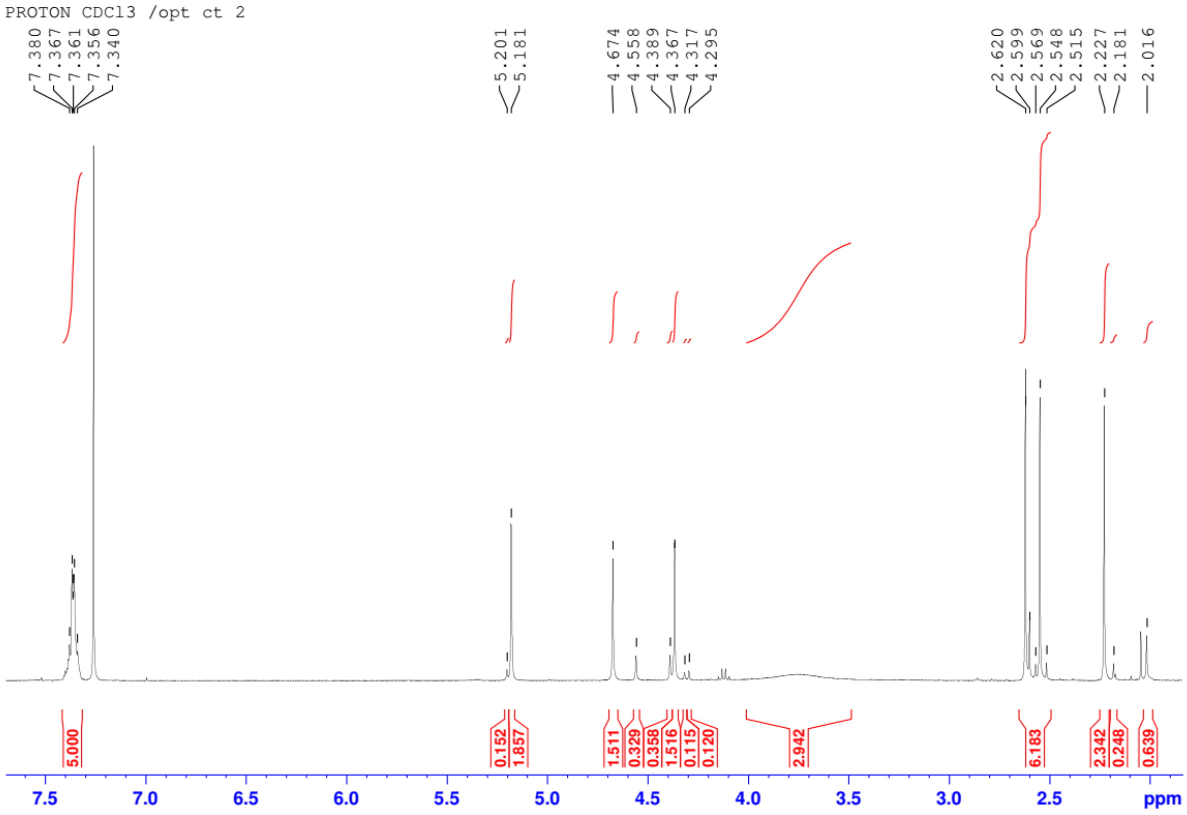

$^{1}H$ NMR, $CD_3CN$, 8 mM

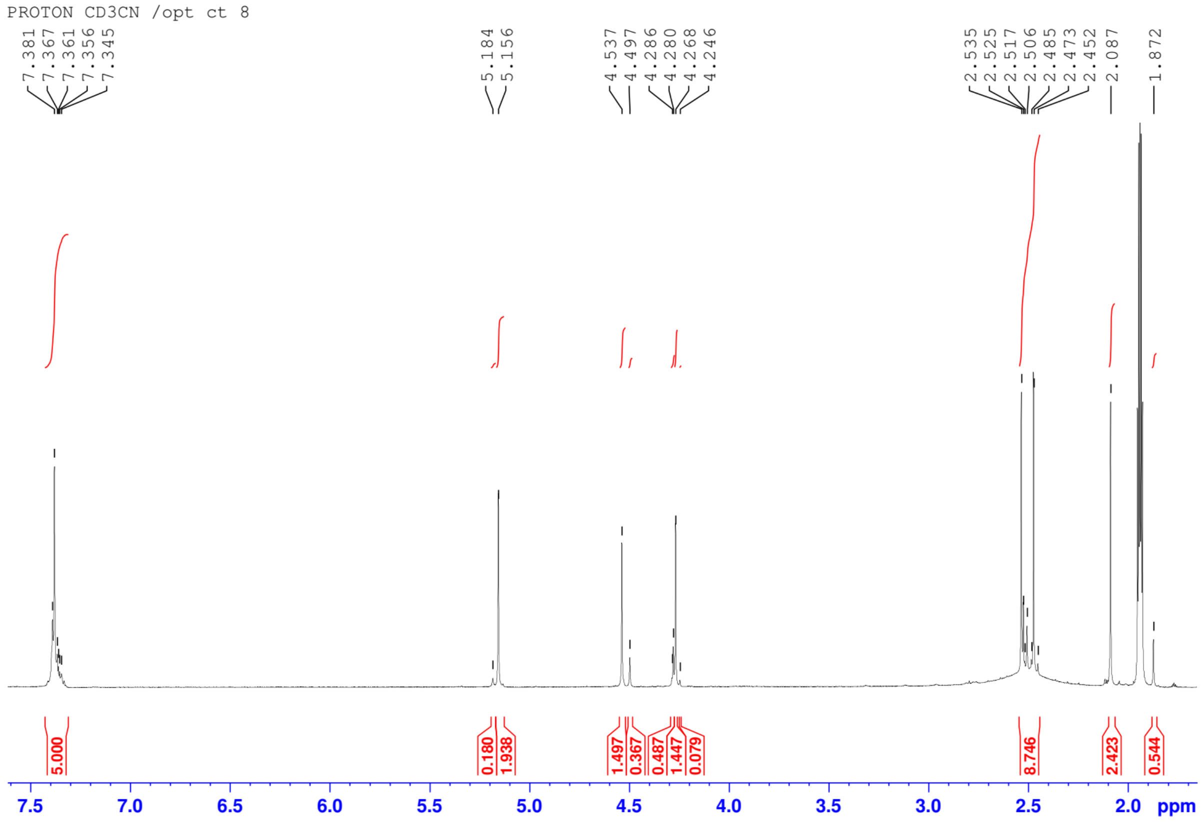

$^{1}H$ NMR, $C_6D_6$, 8 mM

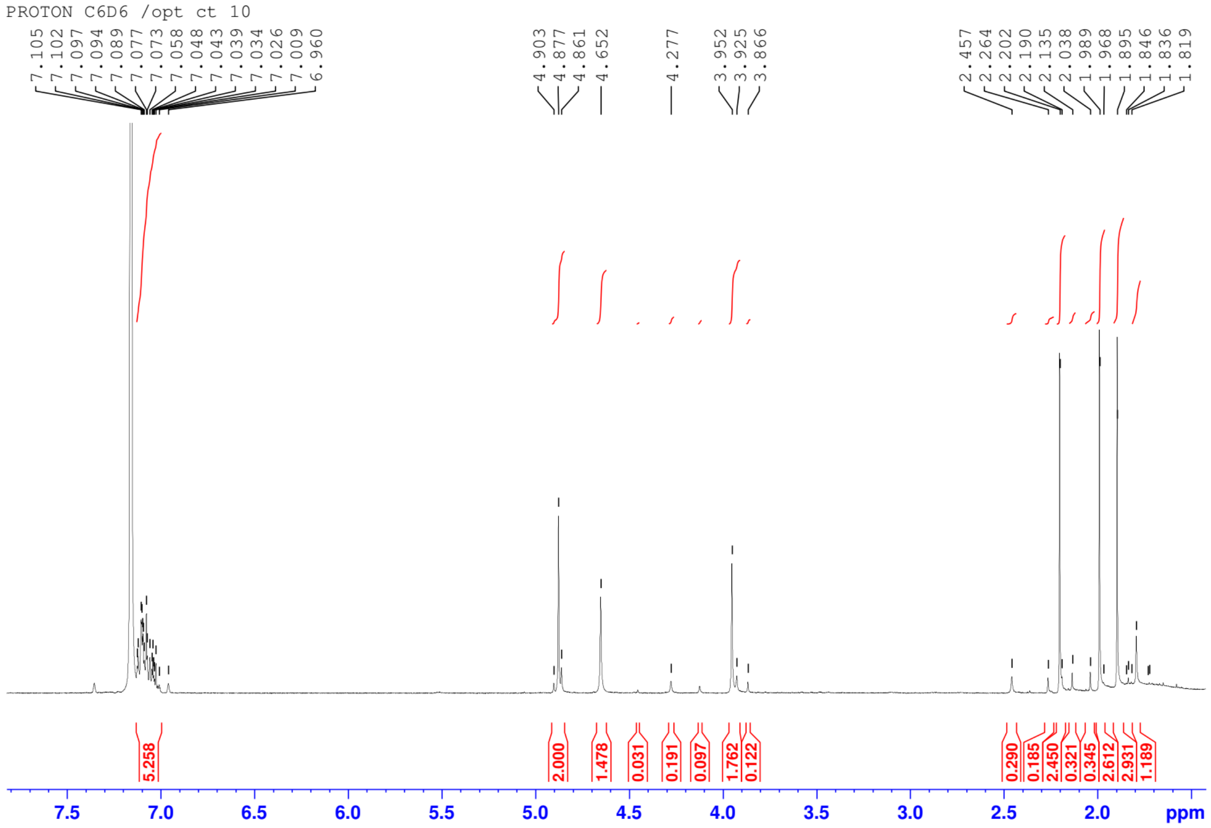

$^{1}H$ NMR, DMSO-$d_6$, 8 mM

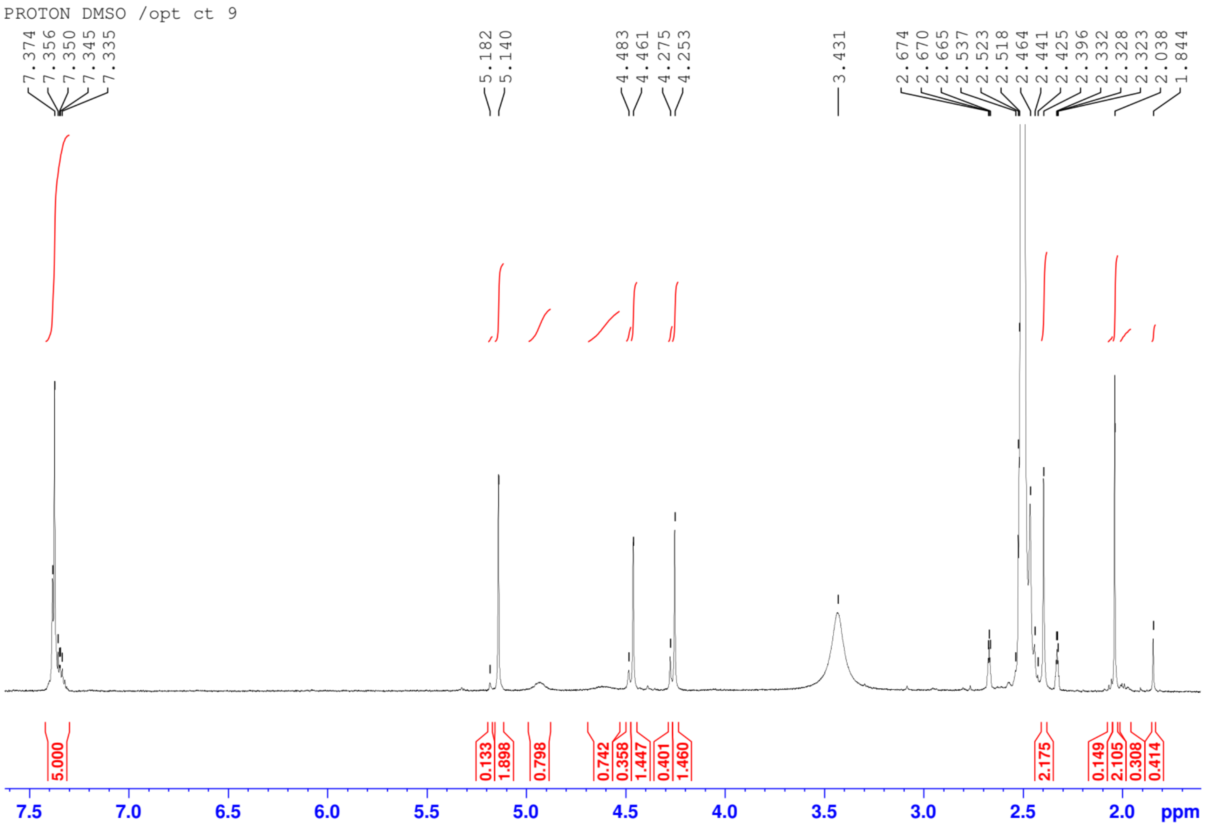


$^{1}H$ NMR, $CD_3OD$, 8 mM

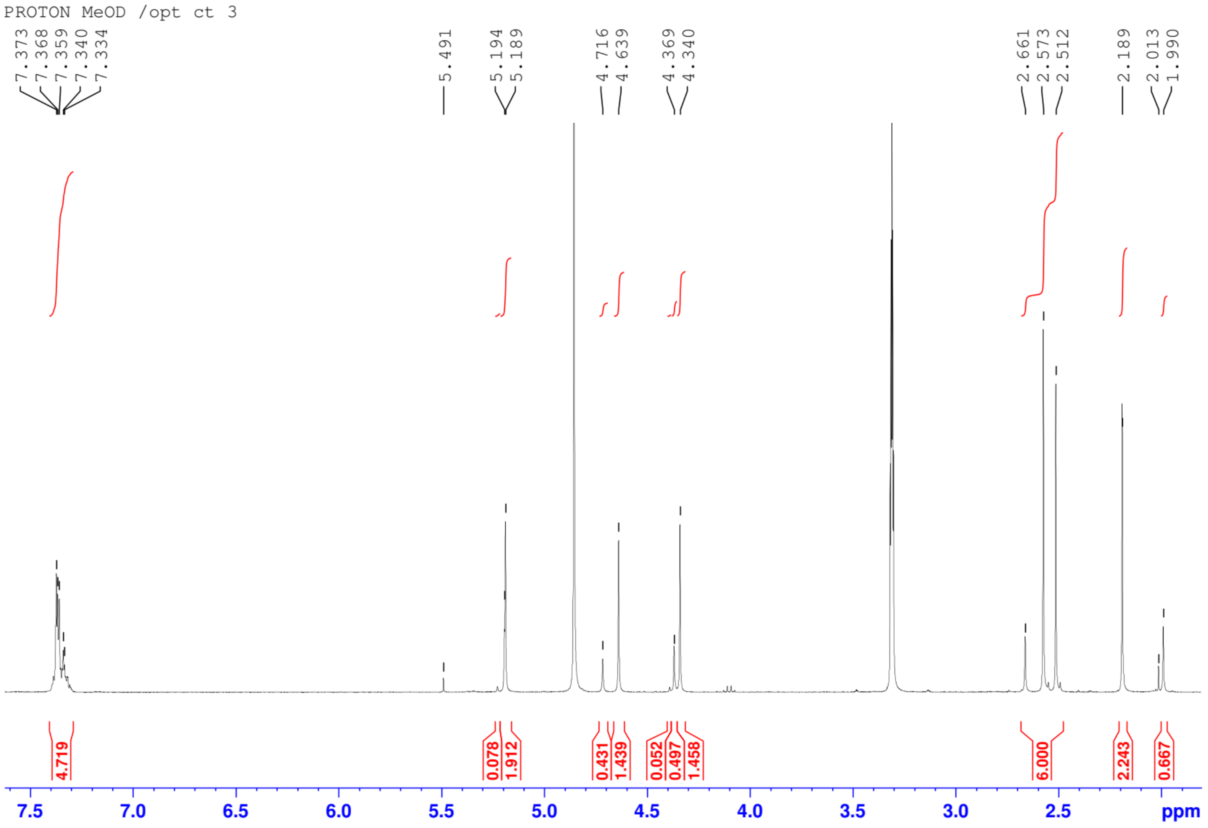

$^{1}H$ NMR, $D_2O$, 8 mM

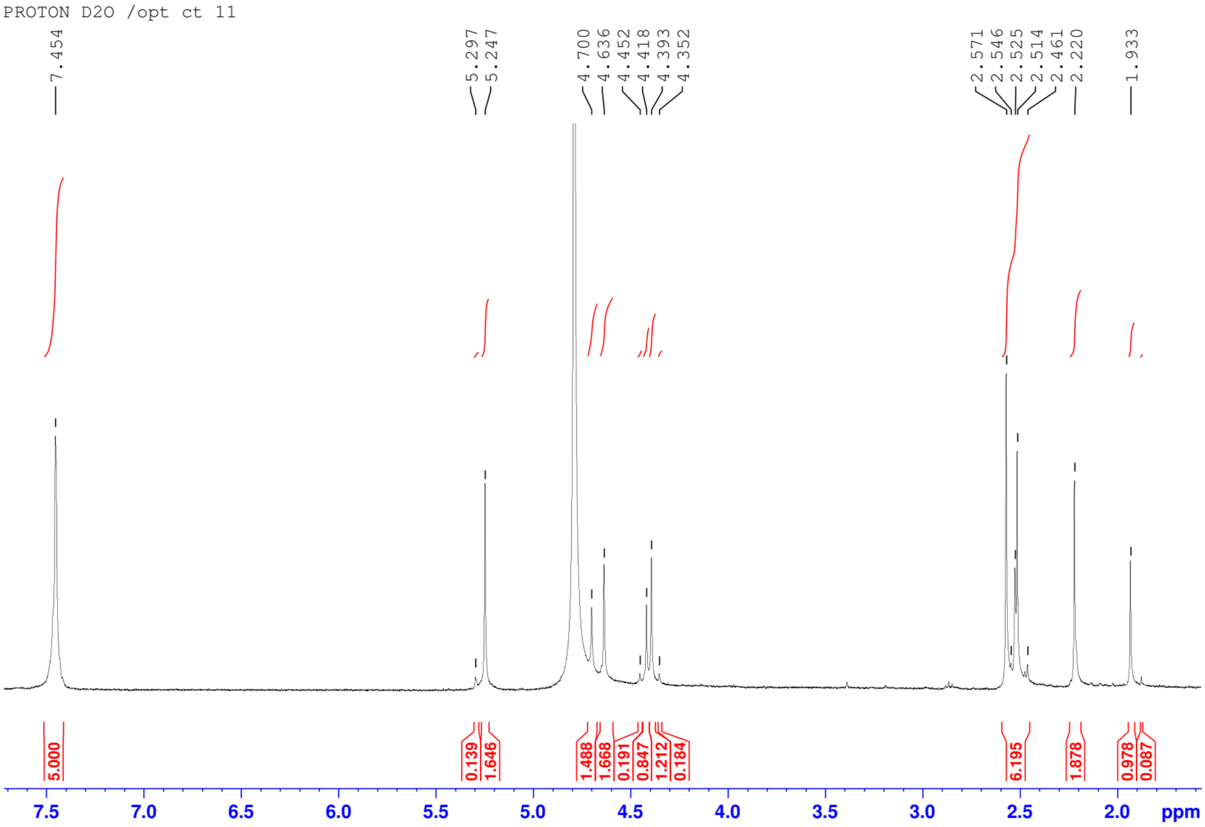


$^{13}C$ NMR, $CDCl_3$

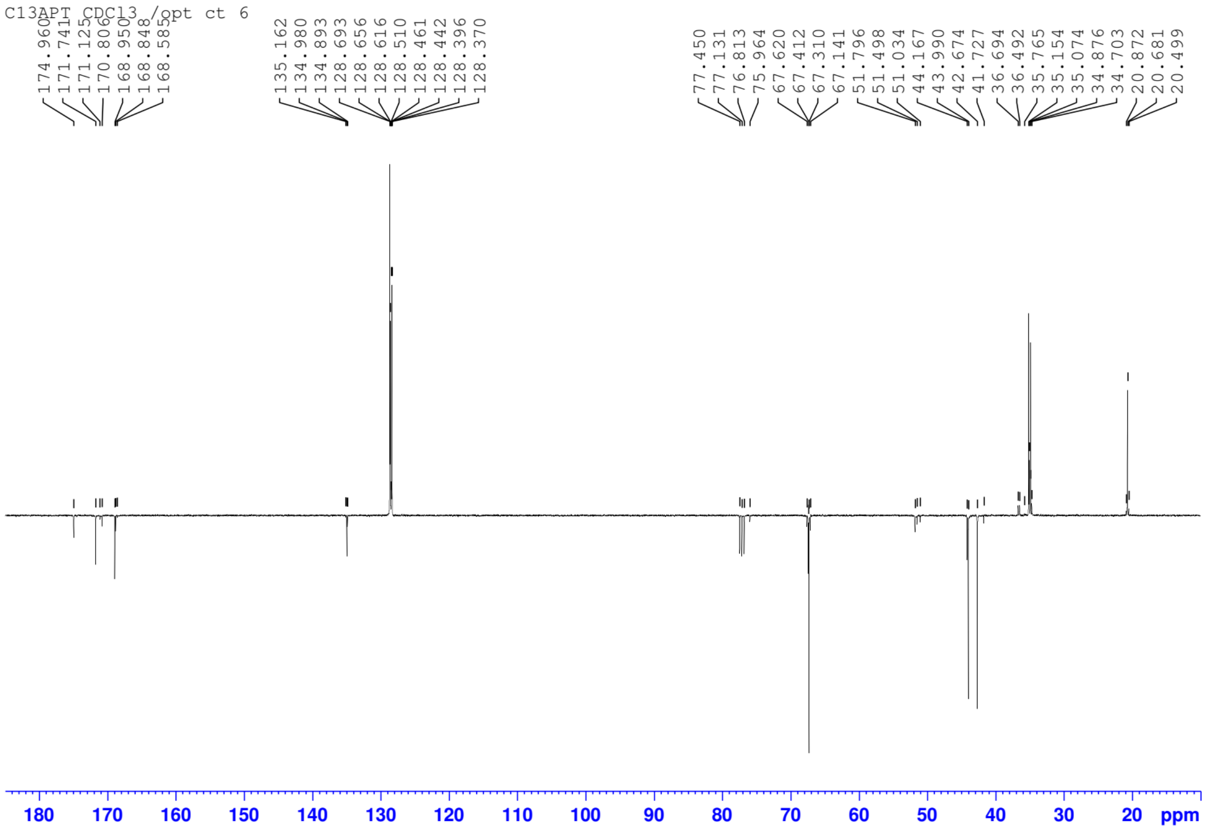

2D HSQC NMR, $CDCl_3$, 5 mM

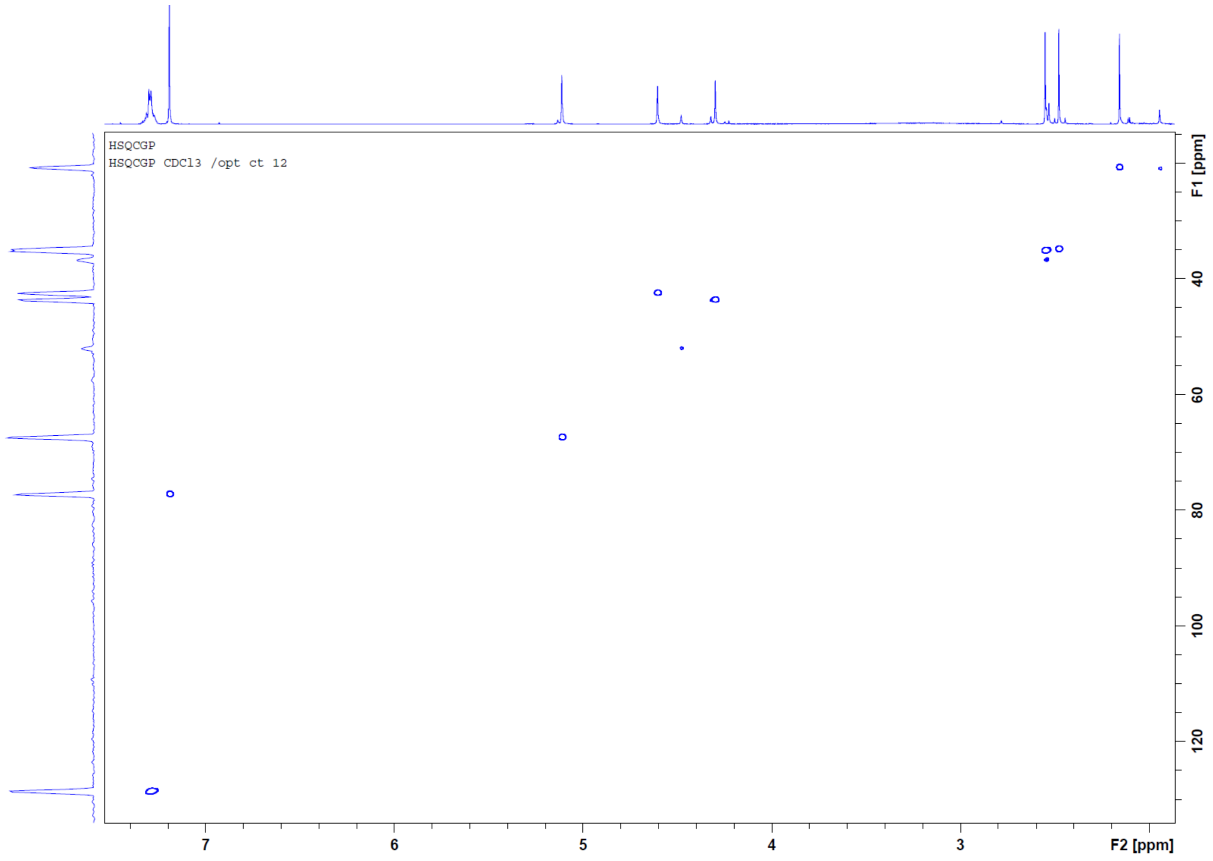


2D HMBC NMR, $CDCl_3$, 5 mM

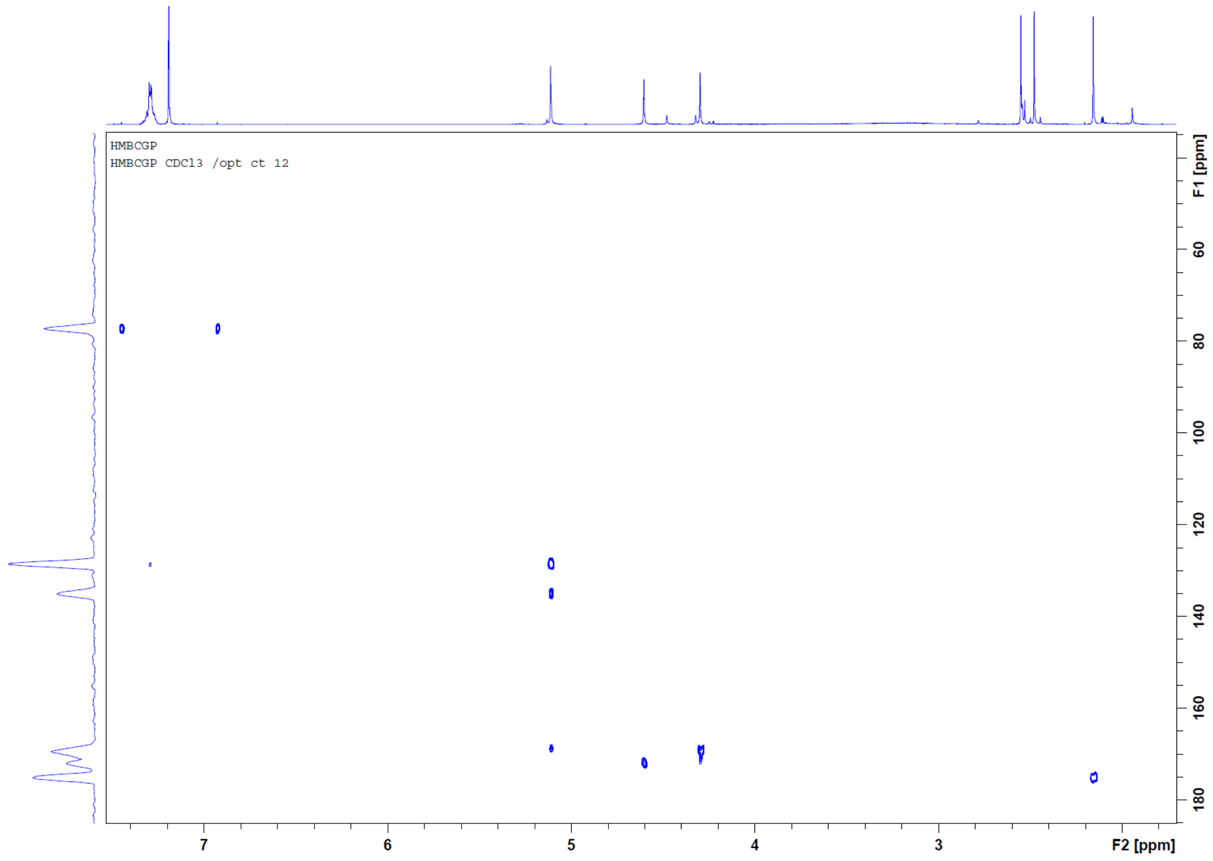

2D NOESY spectrum of peptoid **2** in DMSO-$d_6$, showing a major conformer in the *trans-trans* conformation. The NOESY correlation are shown by colored squares in the spectrum and by colored arrows in the structure drawing.

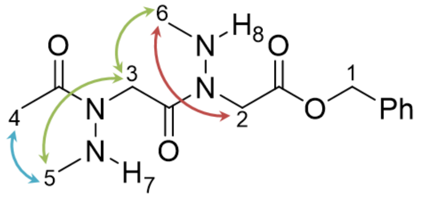

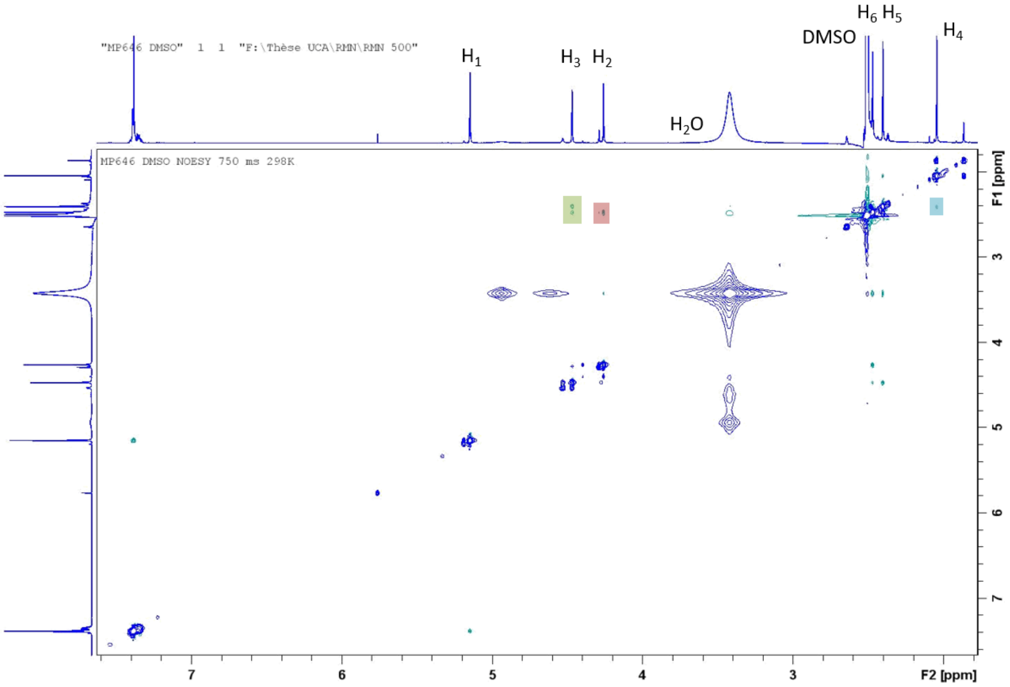


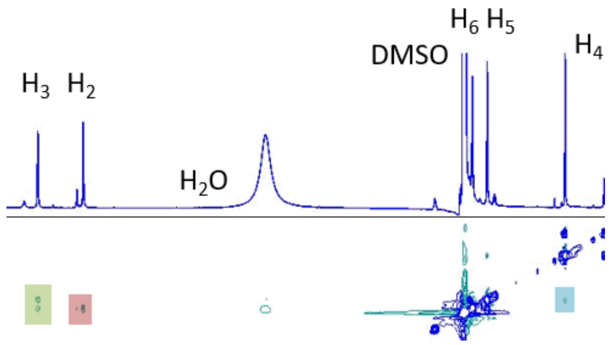

**Trimer 3 (Ac-(*N*NMe)$_3$-OBn)**

$^1$H NMR, $CDCl_3$, 8 mM

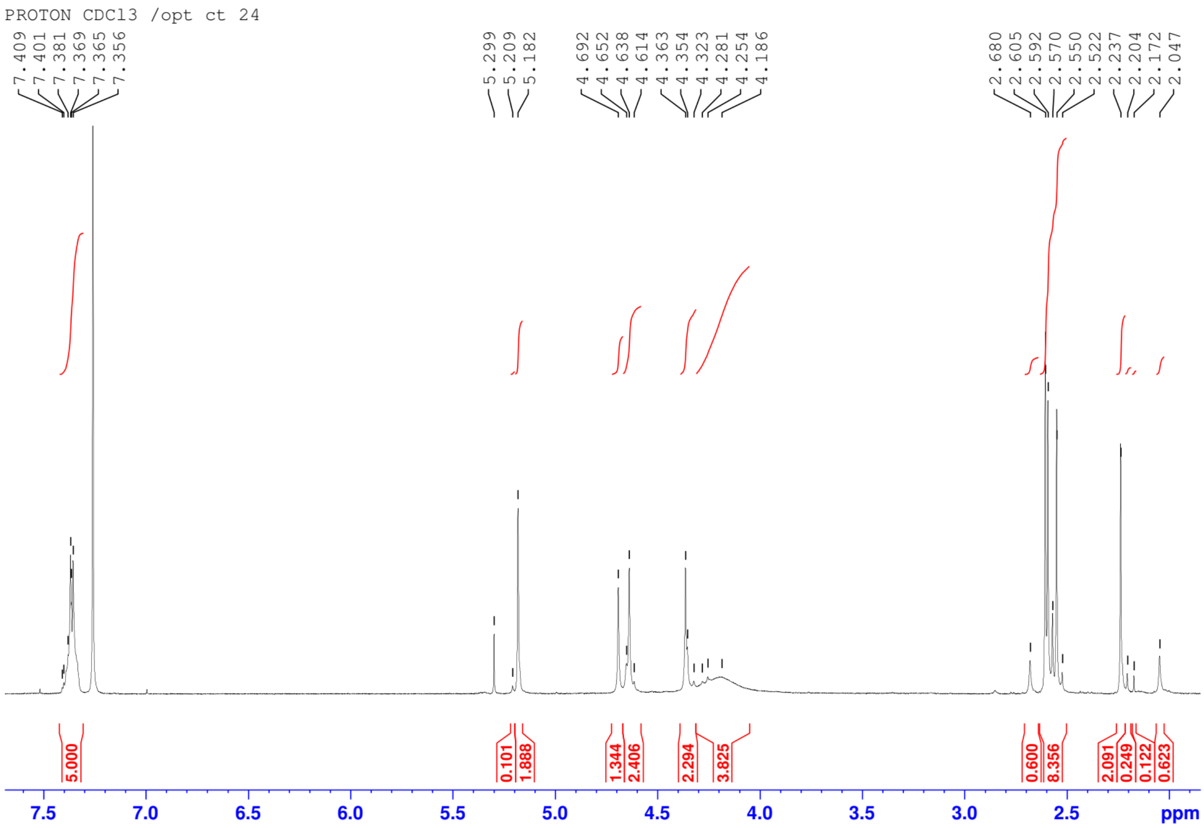

$^1$H NMR, $CD_3CN$, 8 mM

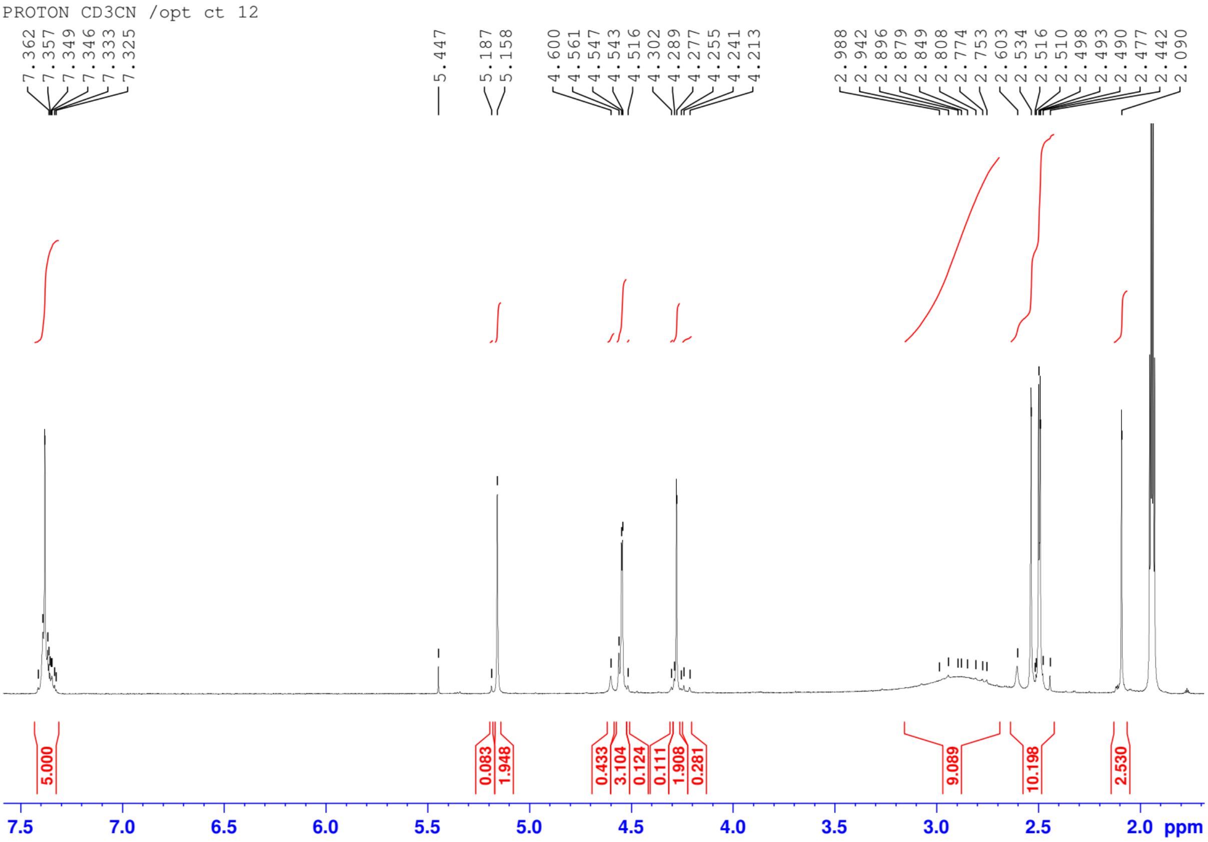

$^{1}H$ NMR, $C_6D_6$, 8 mM

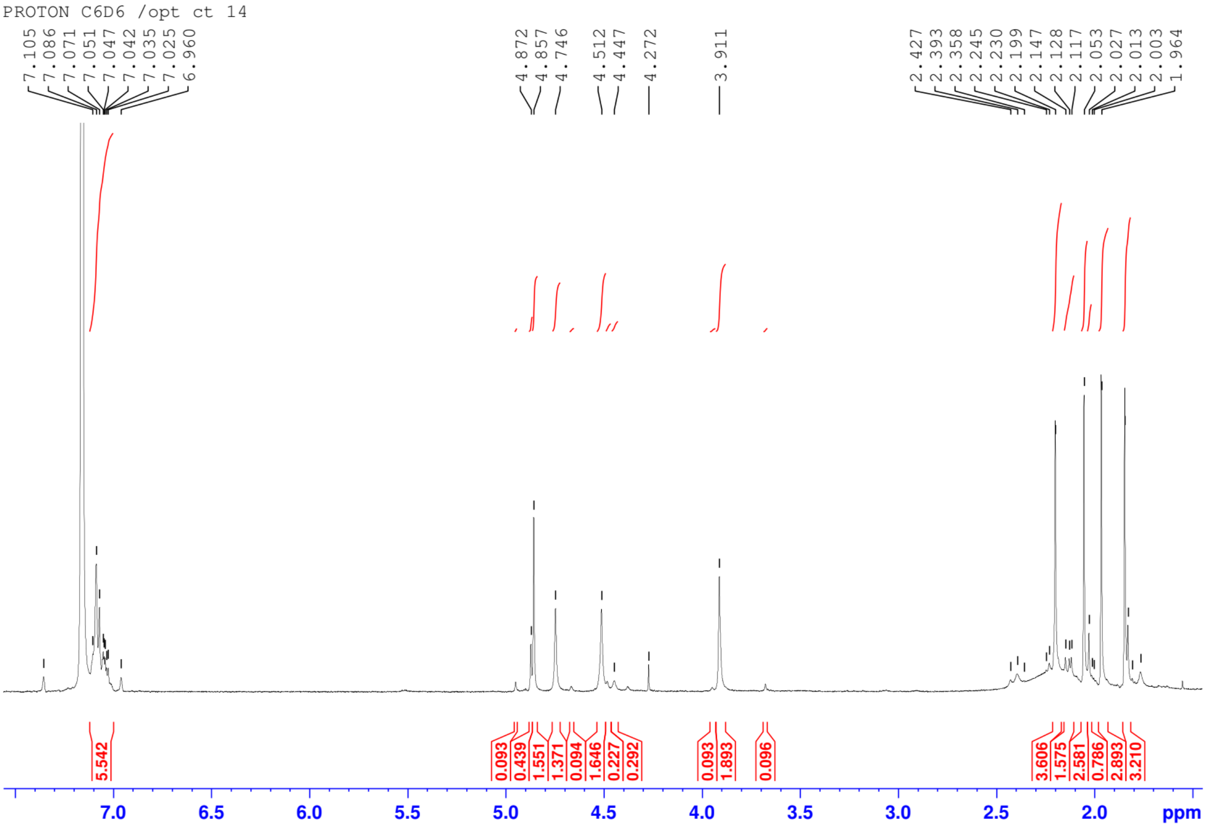


$^{1}H$ NMR, DMSO-$d_6$, 8 mM

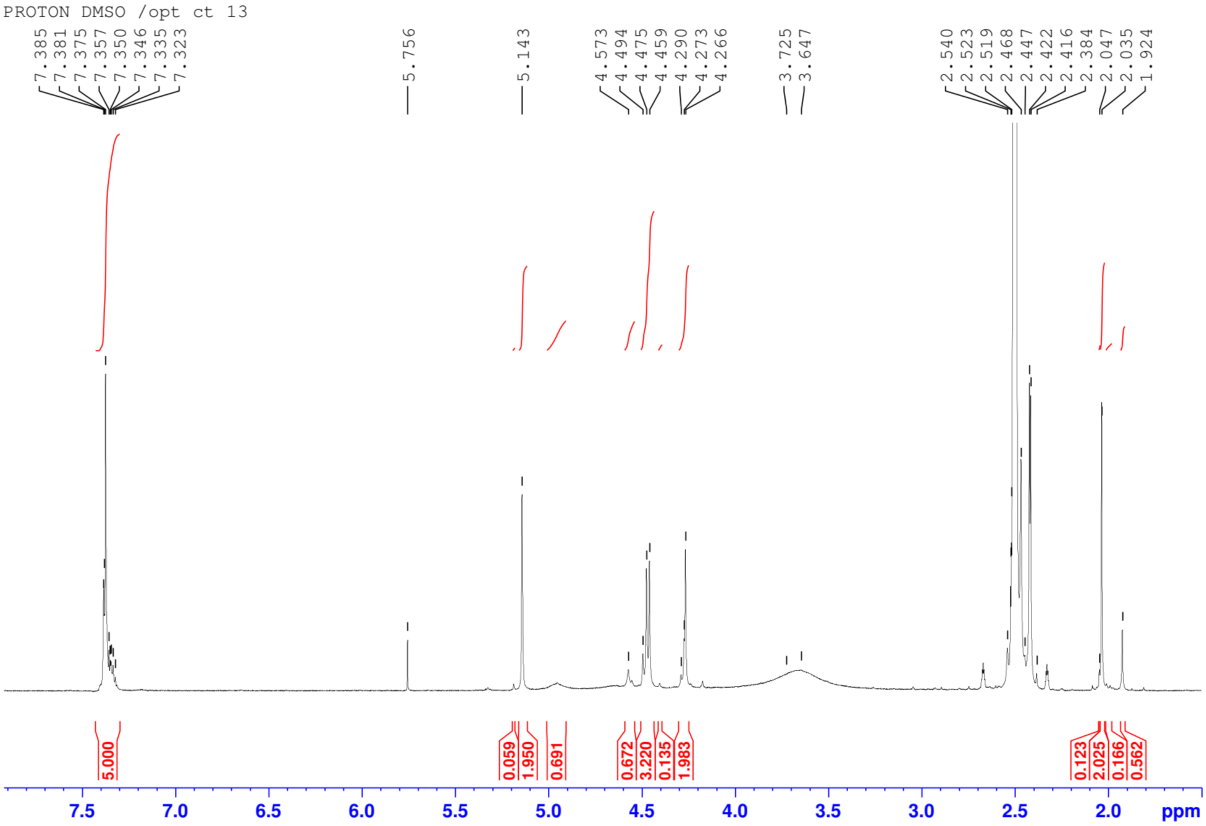

$^{1}H$ NMR, $CD_3OD$, 8 mM

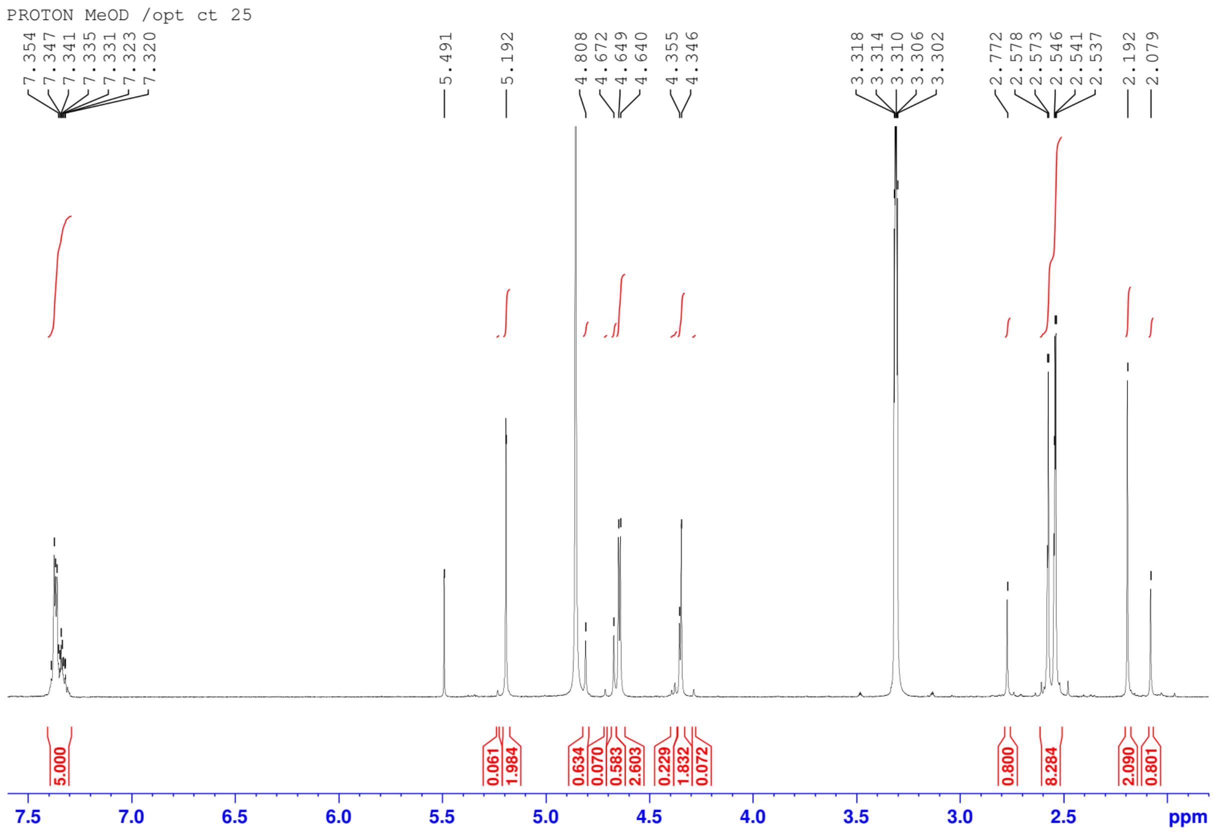


$^{1}H$ NMR, $D_2O$, 8 mM

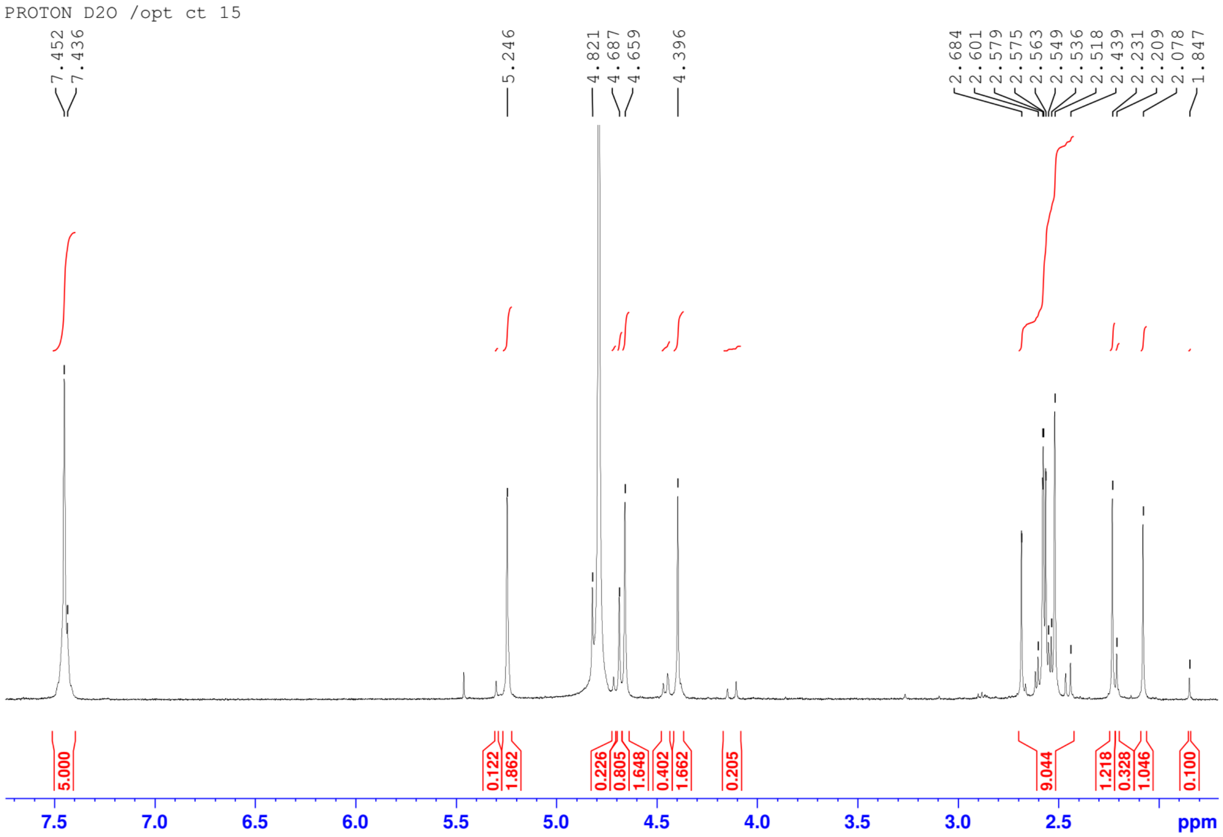

$^{13}C$ NMR, $CDCl_3$

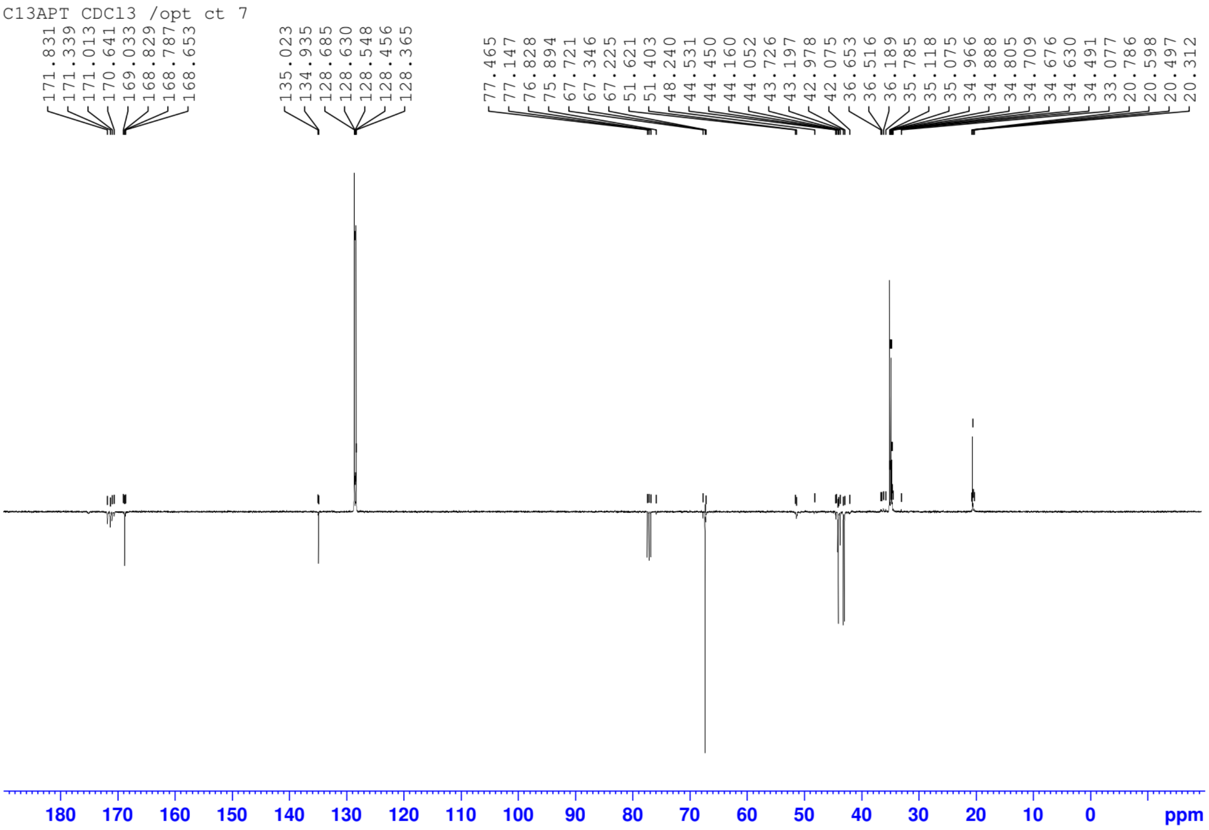


2D HSQC NMR, $CDCl_3$, 5 mM

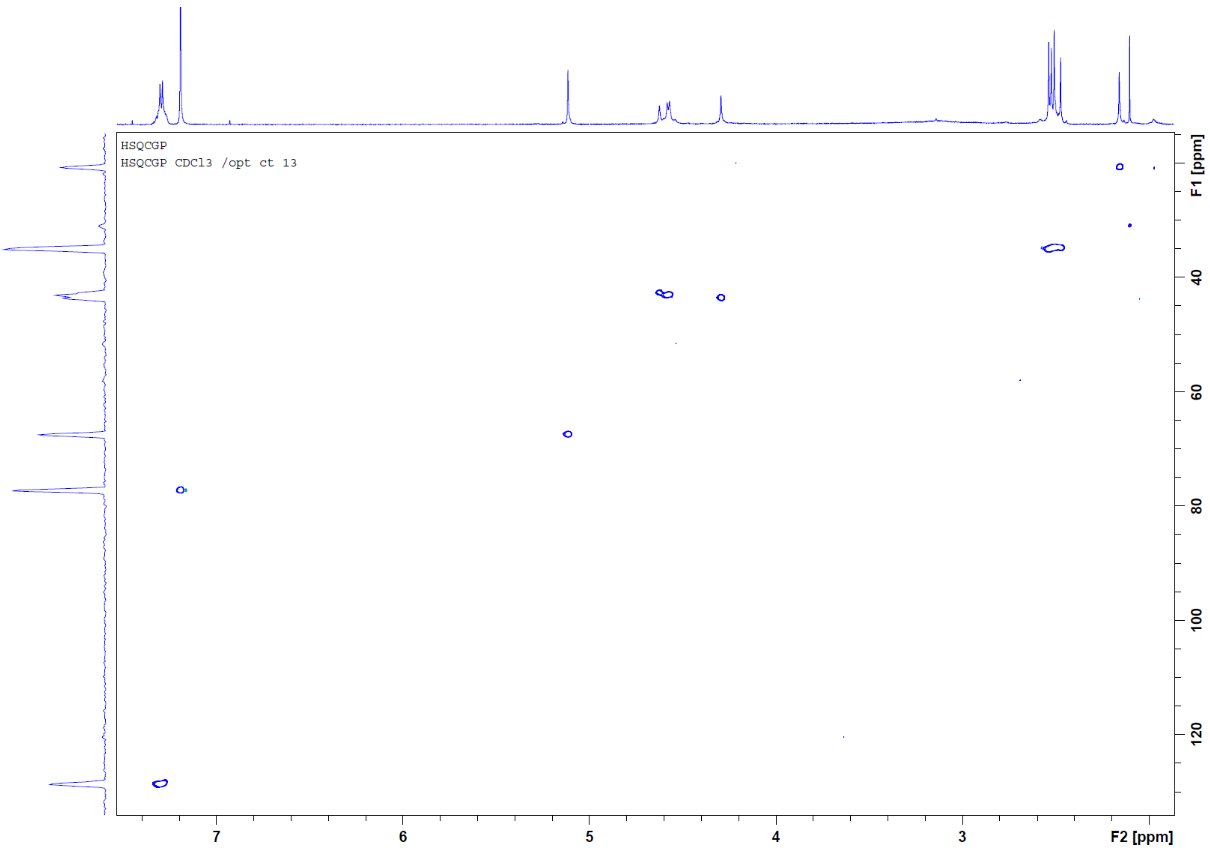

2D HMBC NMR, $CDCl_3$, 5 mM

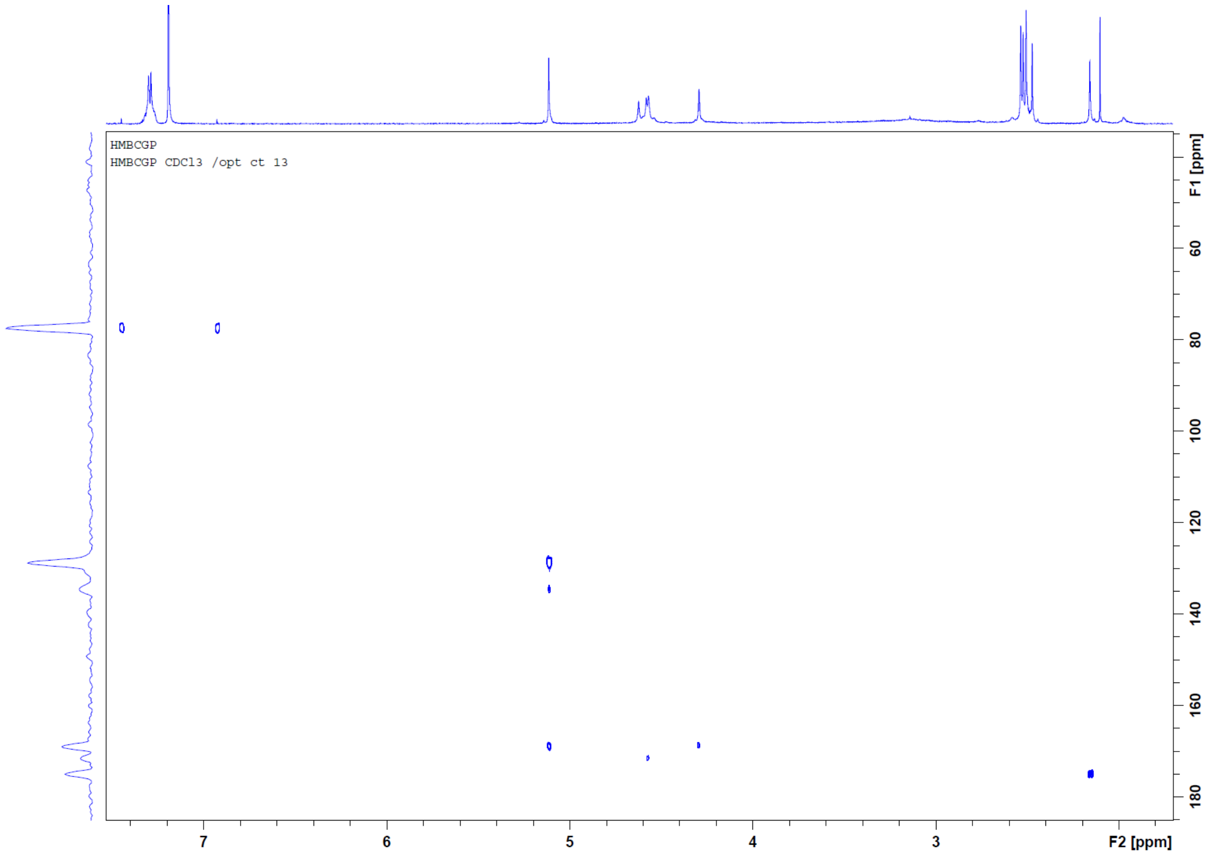

2D NOESY spectrum of peptoid **3** in DMSO-$d_6$ (5 mM)

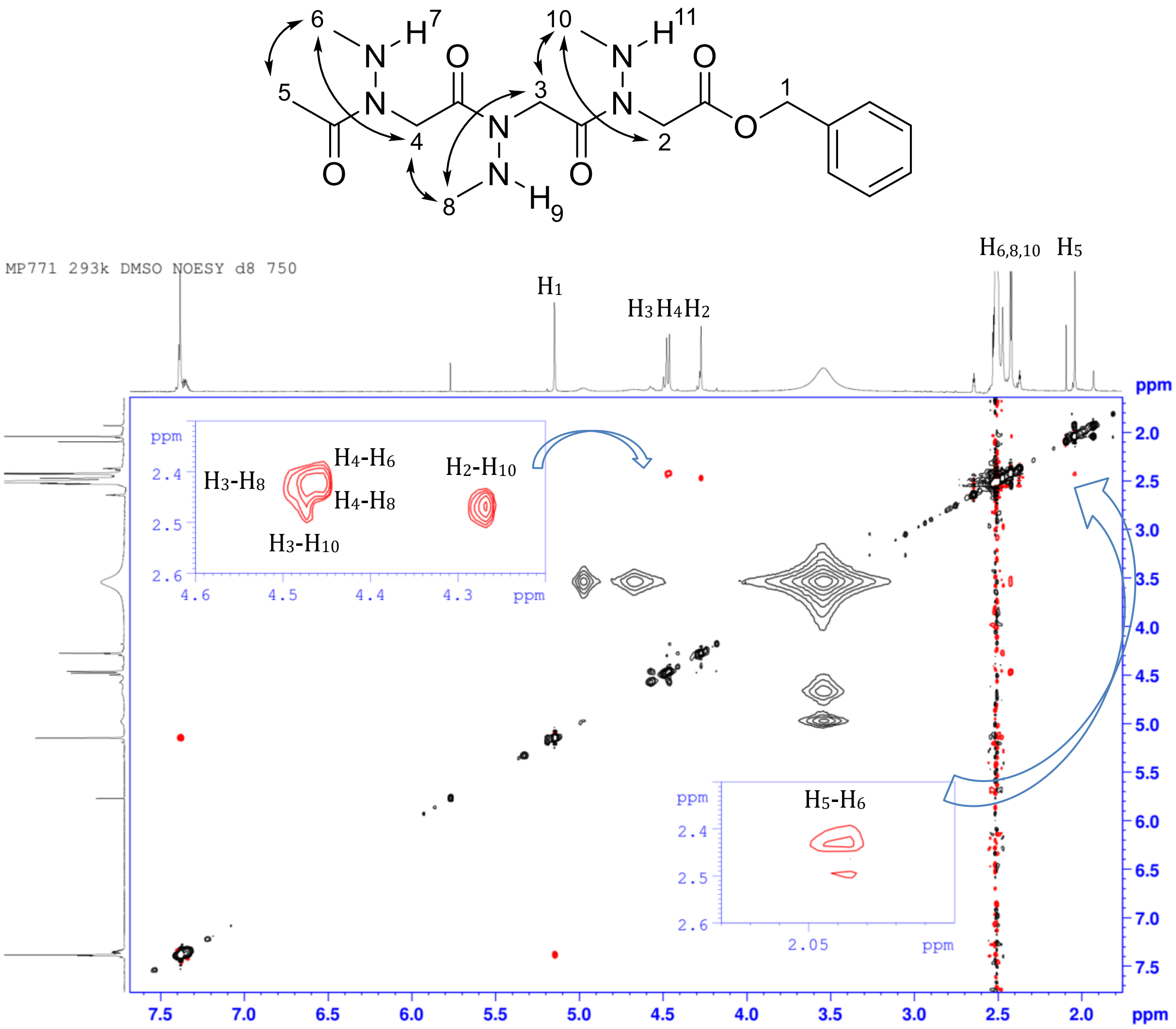

**Tetramer 4 (Ac-(*N*NMe)$_4$-OBn)**

$^1$H NMR, $CD_3OD$, 8 mM

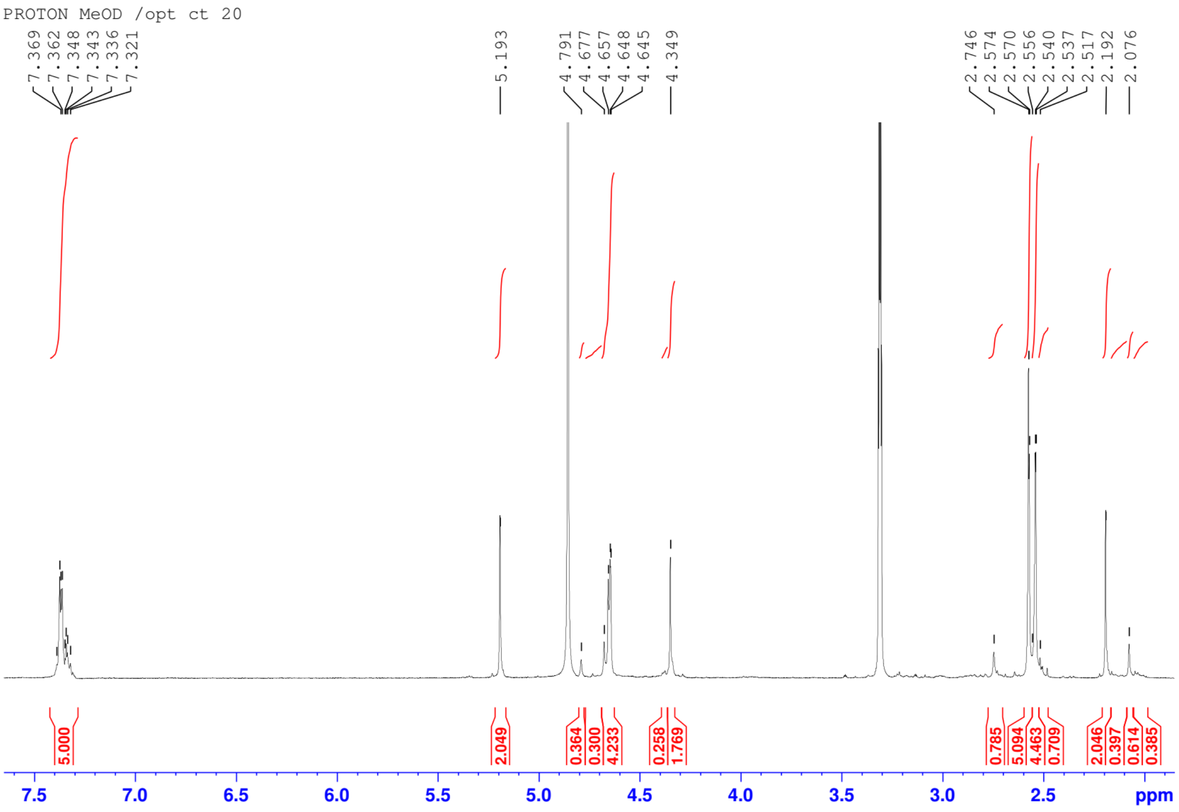


$^1$H NMR, DMSO-$d_6$, 8 mM

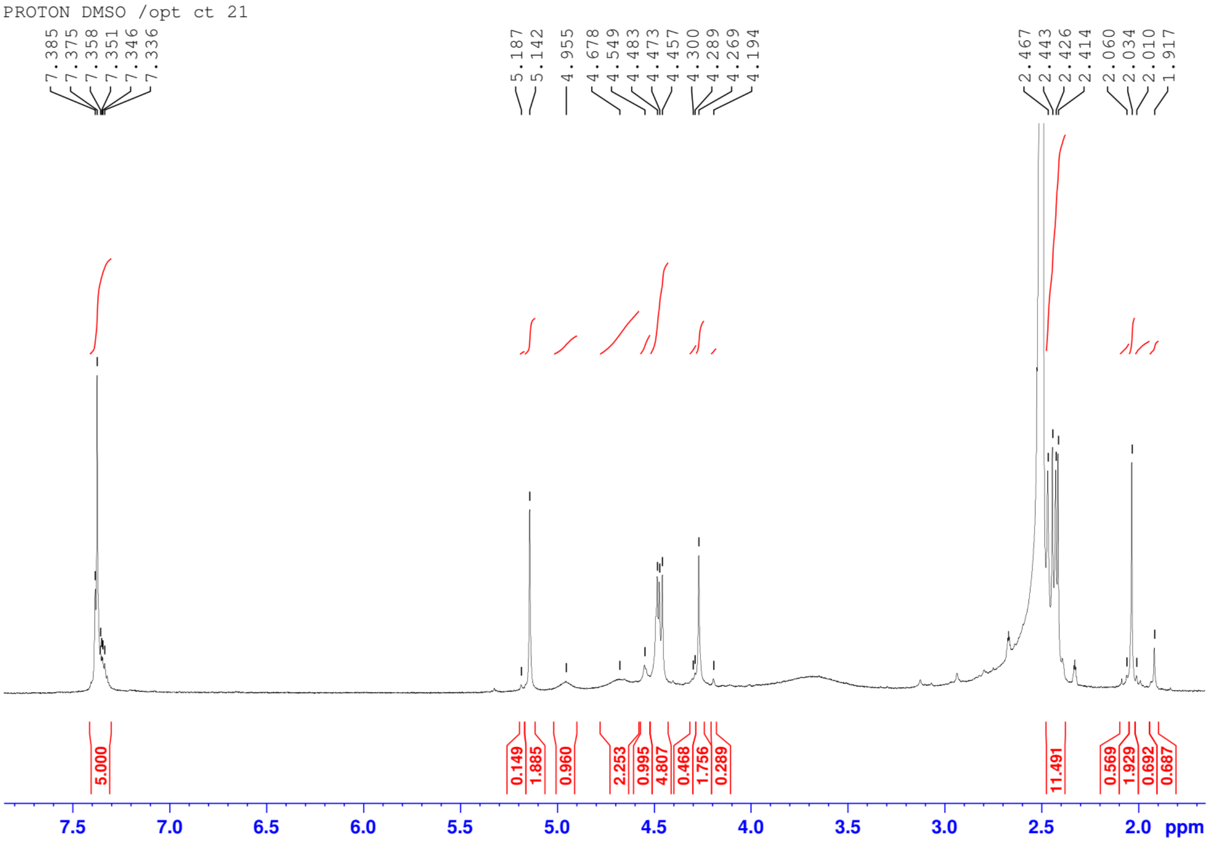

$^{13}C$ NMR, $CDCl_3$

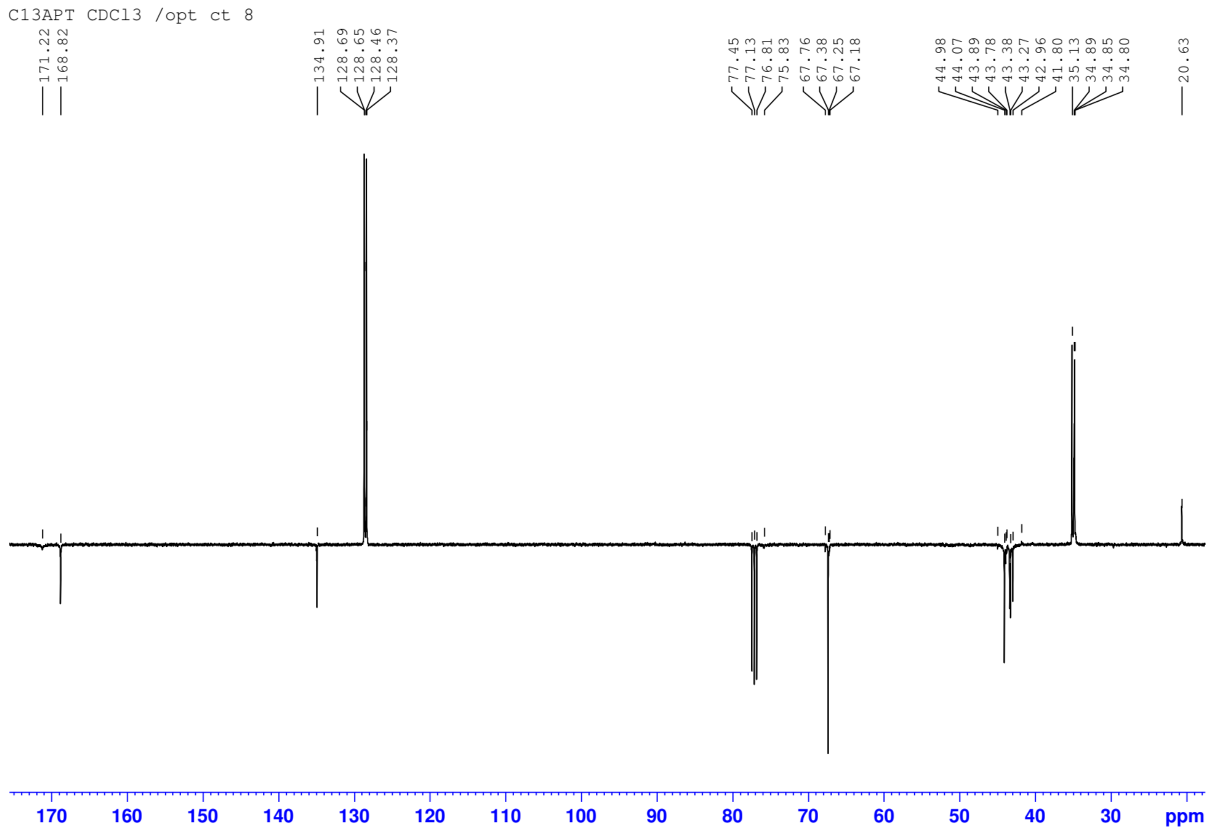


2D HSQC NMR, $CDCl_3$, 8 mM

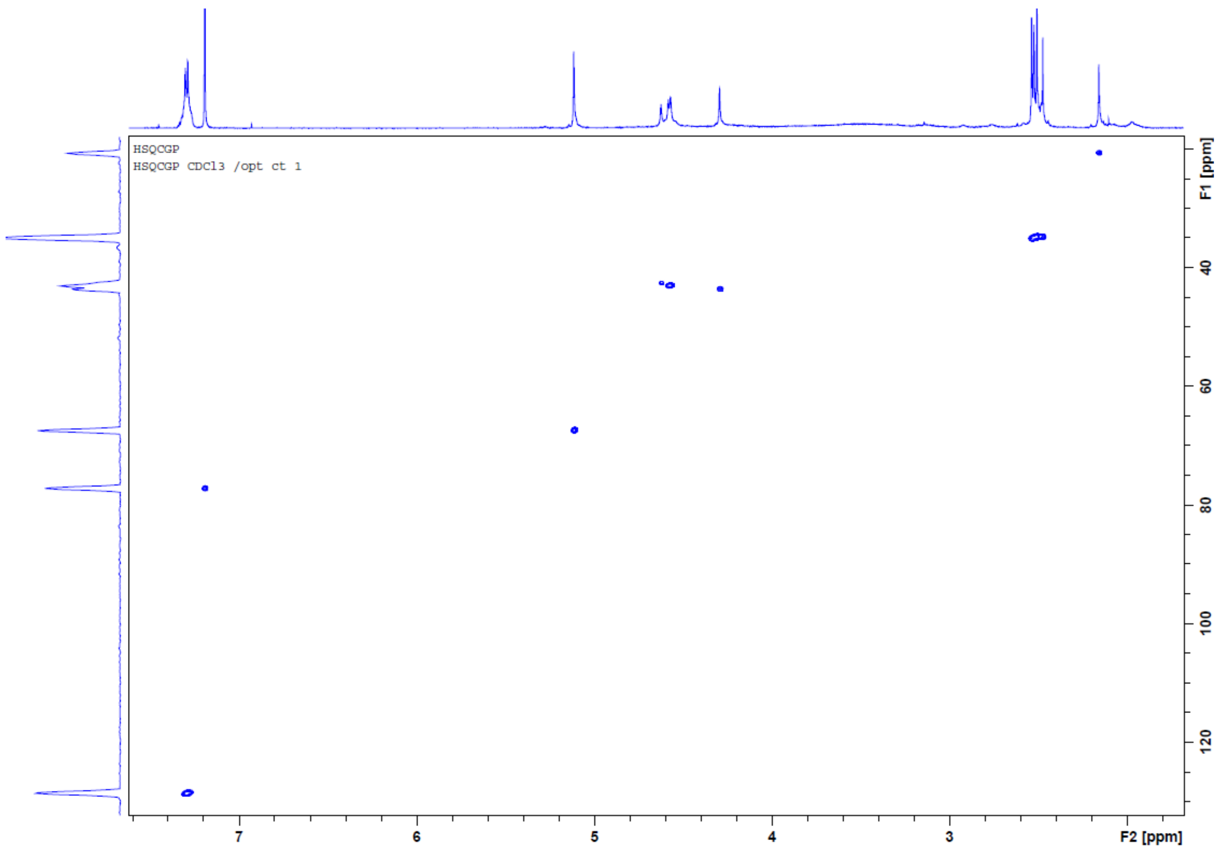

2D HMBC NMR, $CDCl_3$, 8 mM

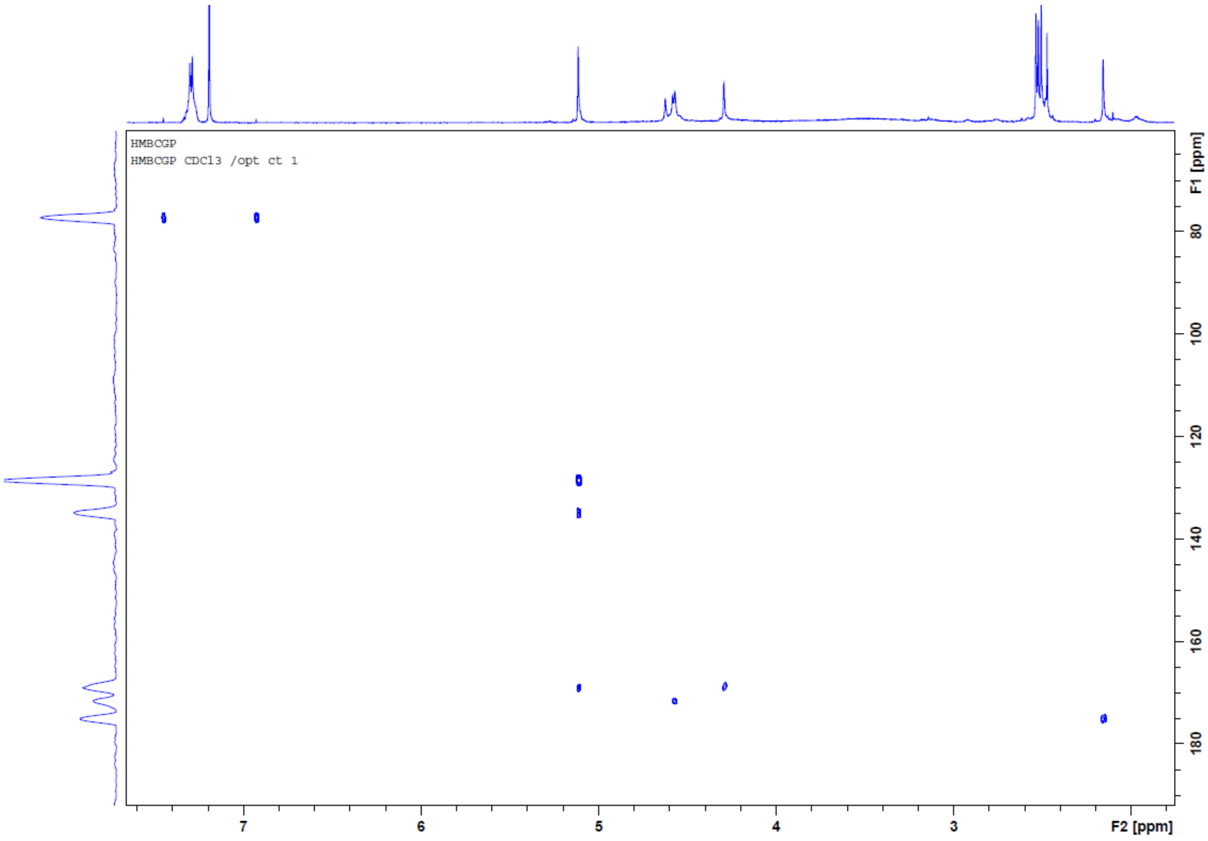

**Pentamer 5 (Ac-(*N*NMe)$_5$-OBn)**

$^1H$ NMR, $CD_3OD$, 8 mM

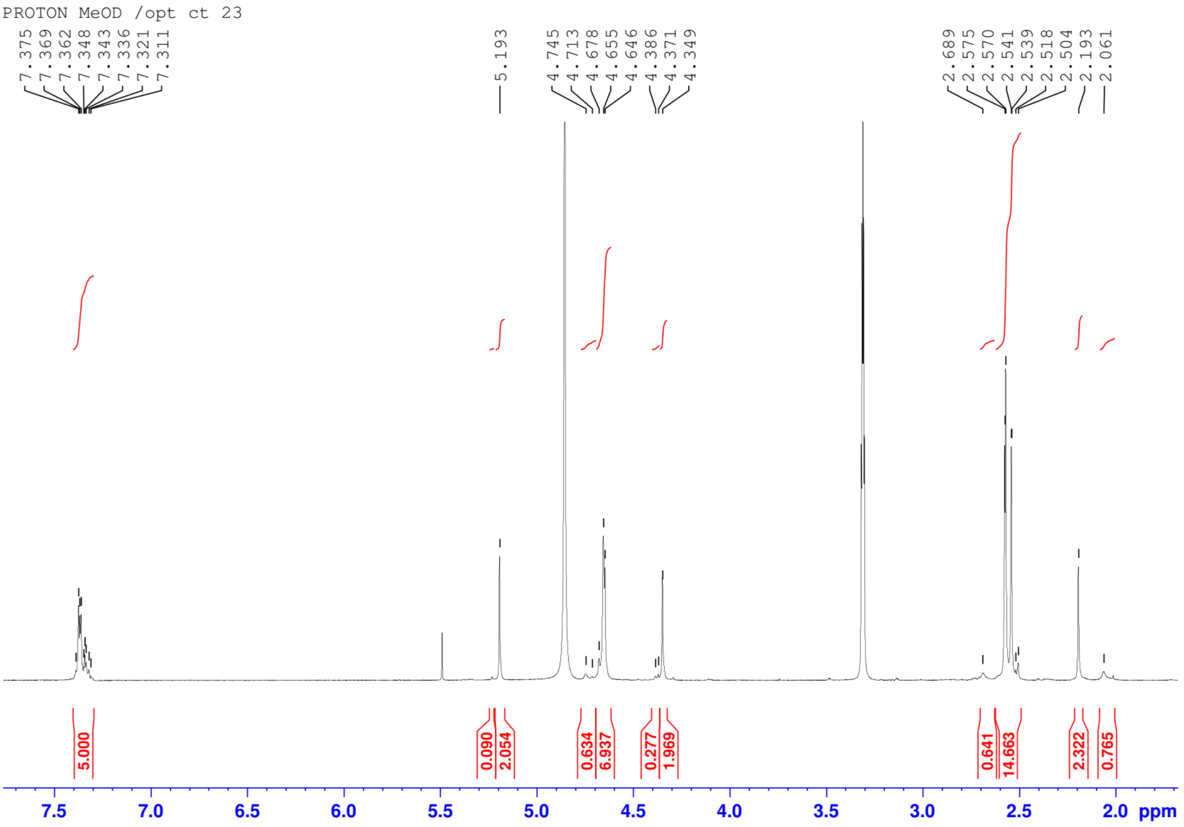

$^{1}H$ NMR, DMSO-$d_6$, 8 mM

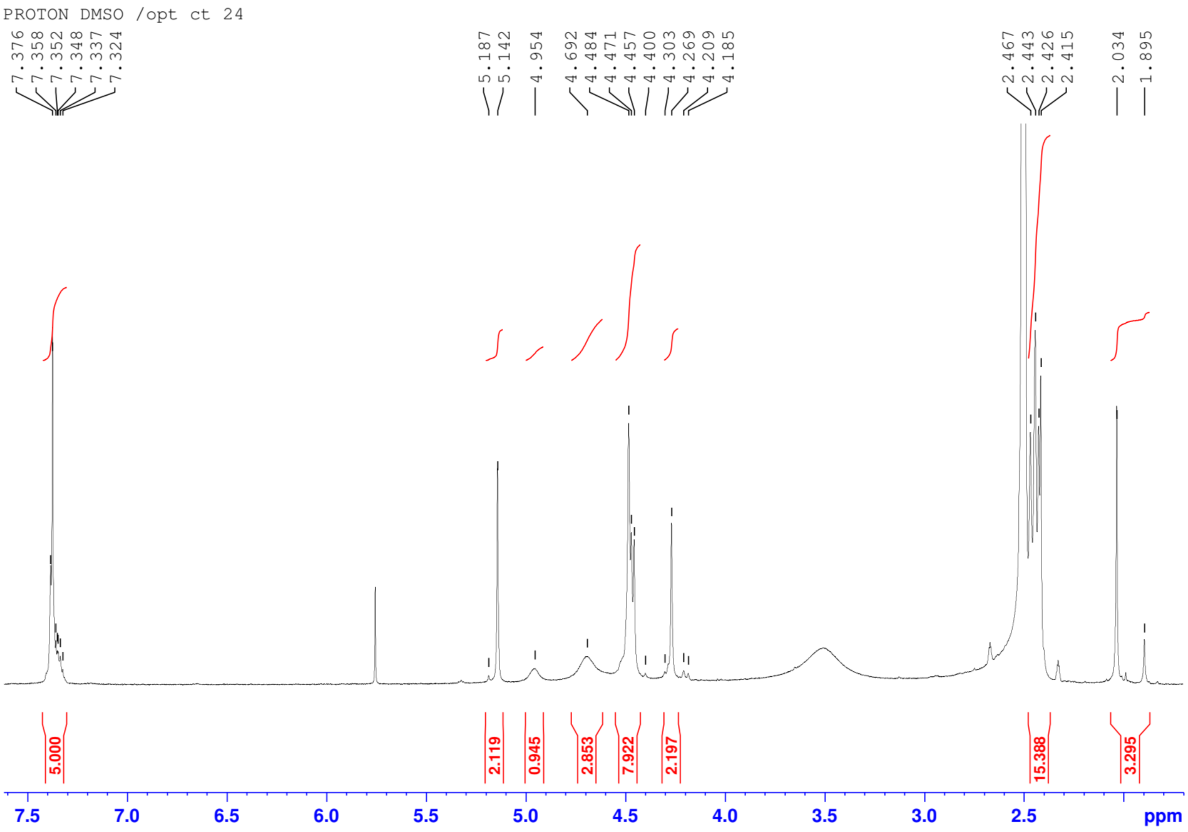


$^{13}C$ NMR, $CDCl_3$

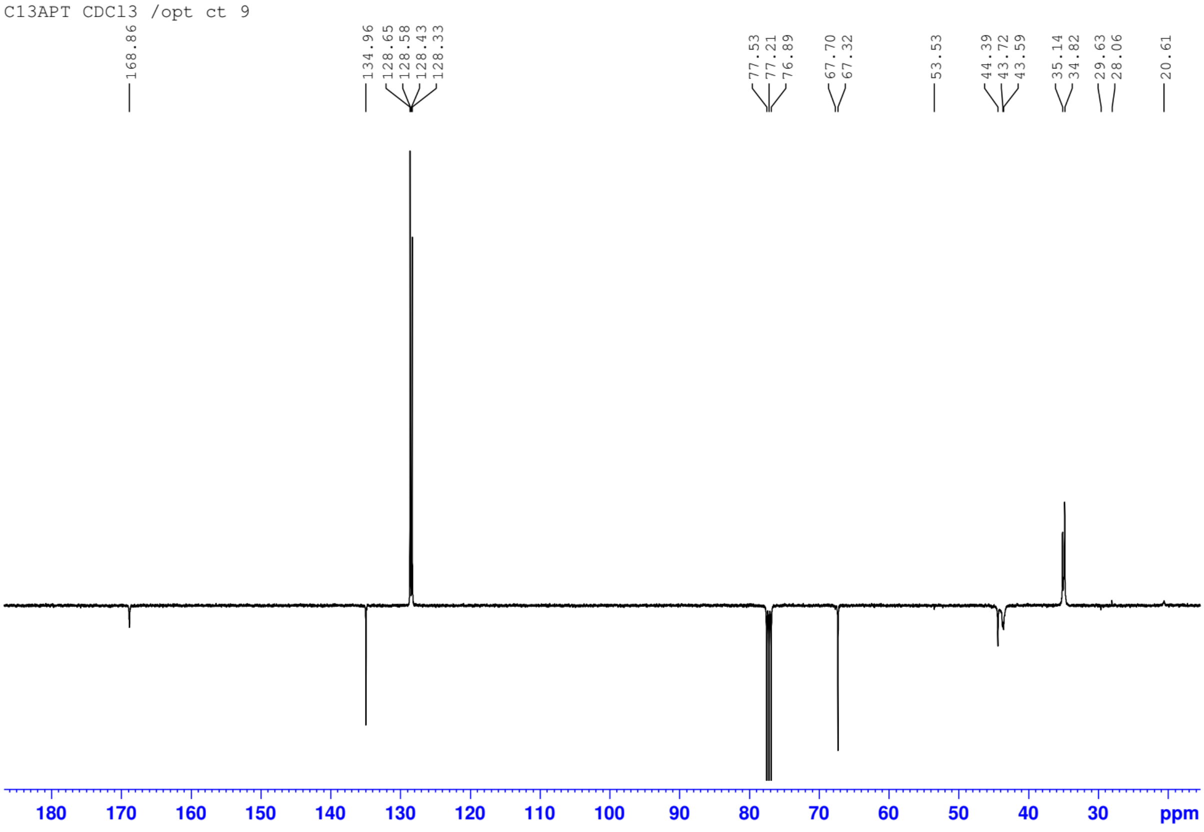

2D HSQC NMR, $CDCl_3$, 8 mM

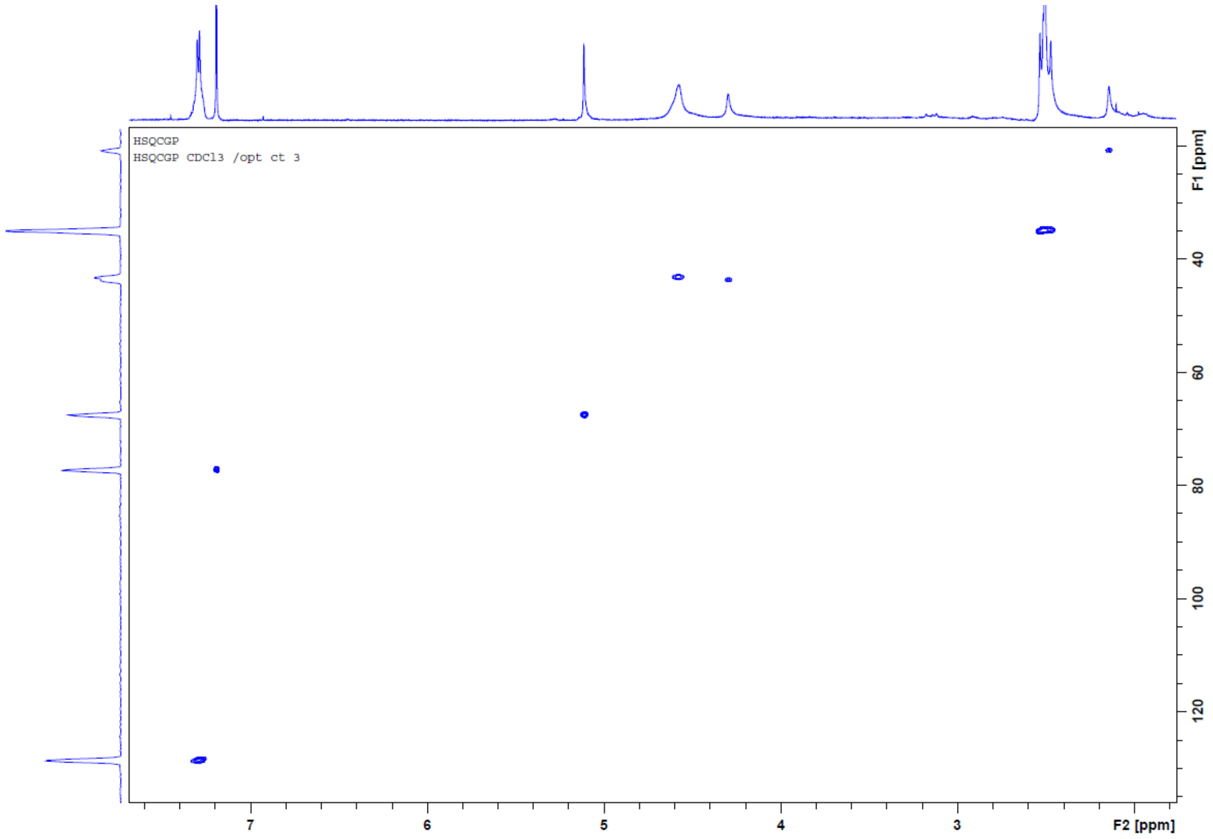


2D HMBC NMR, $CDCl_3$, 8 mM

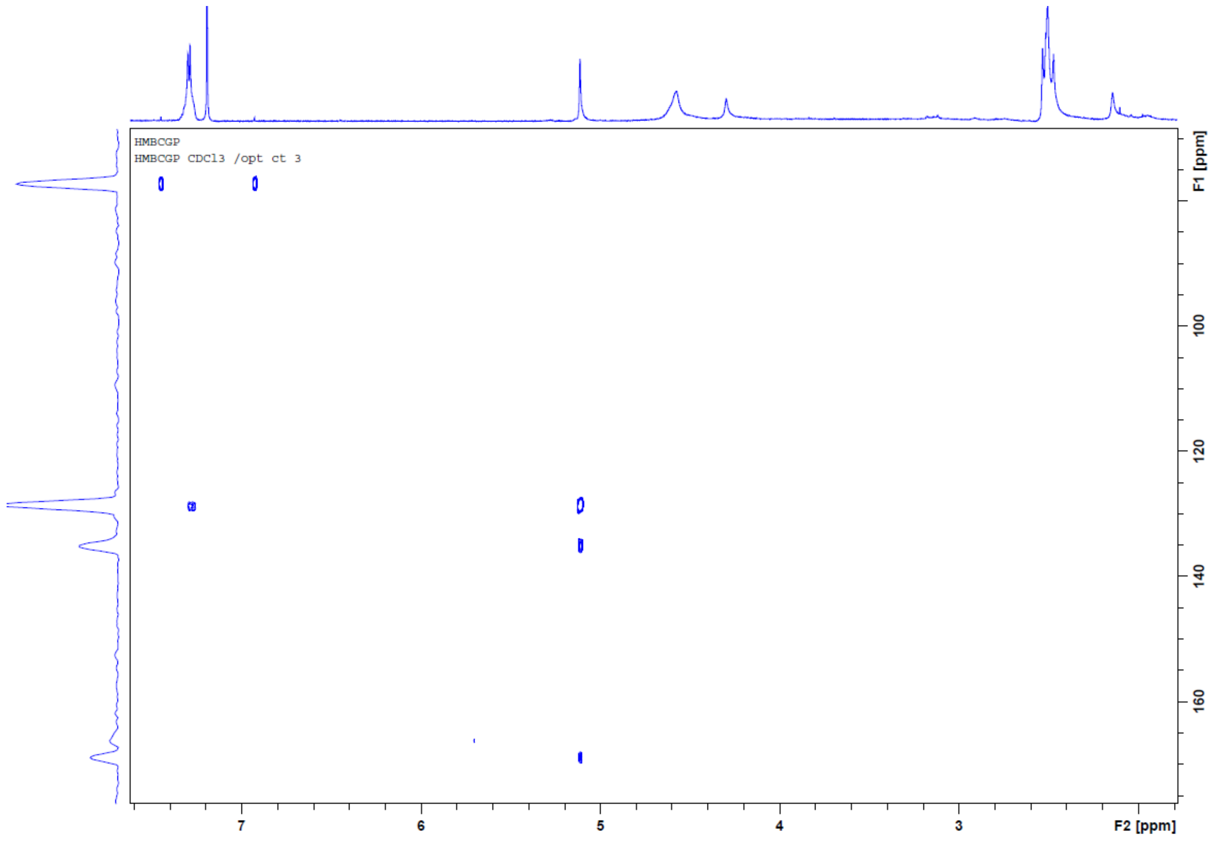

**Hexamer 6 (Ac-(*N*NMe)$_6$-OBn)**

$^{1}$H NMR, DMSO-$d_6$, 15 mM

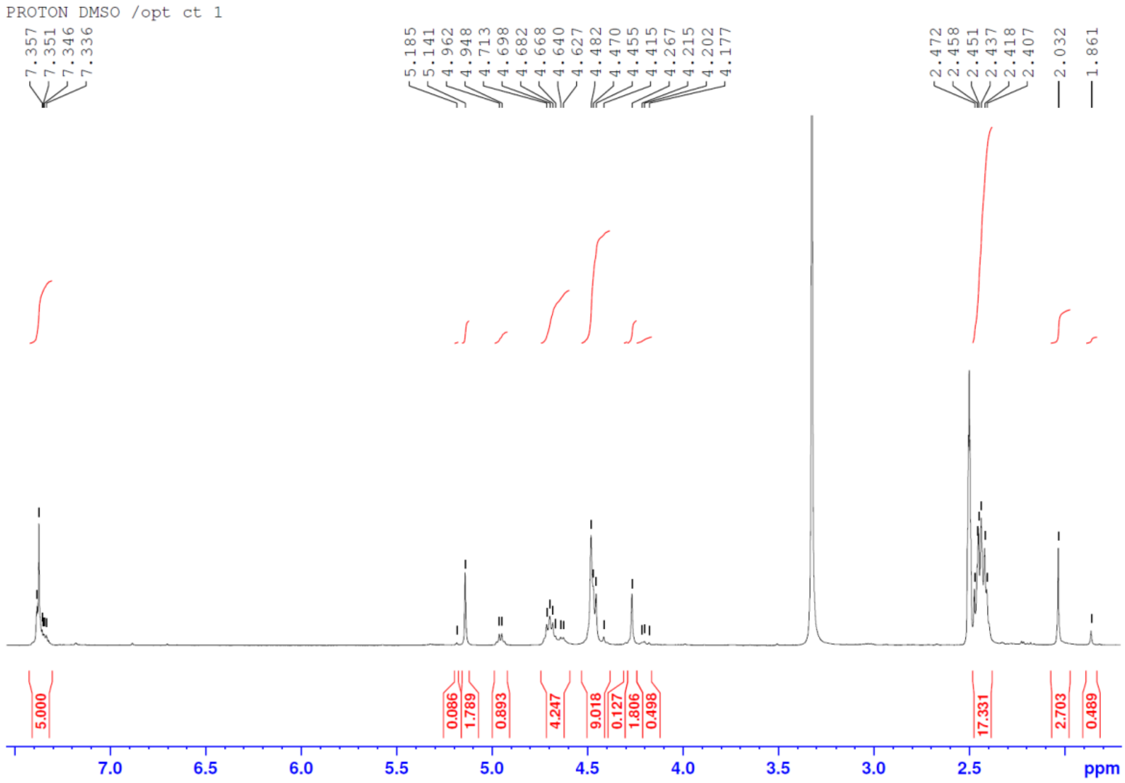


2D HSQC NMR, DMSO-$d_6$, 15 mM

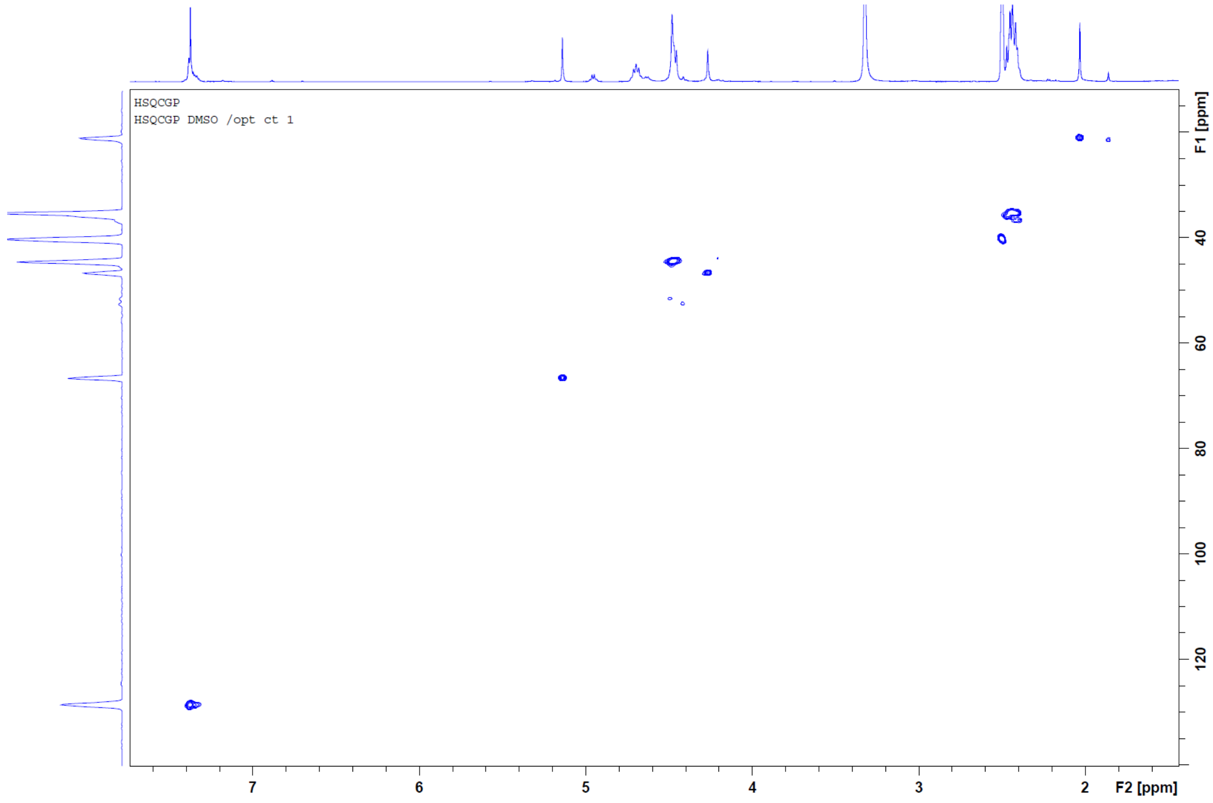

2D HMBC NMR, DMSO-$d_6$, 15 mM

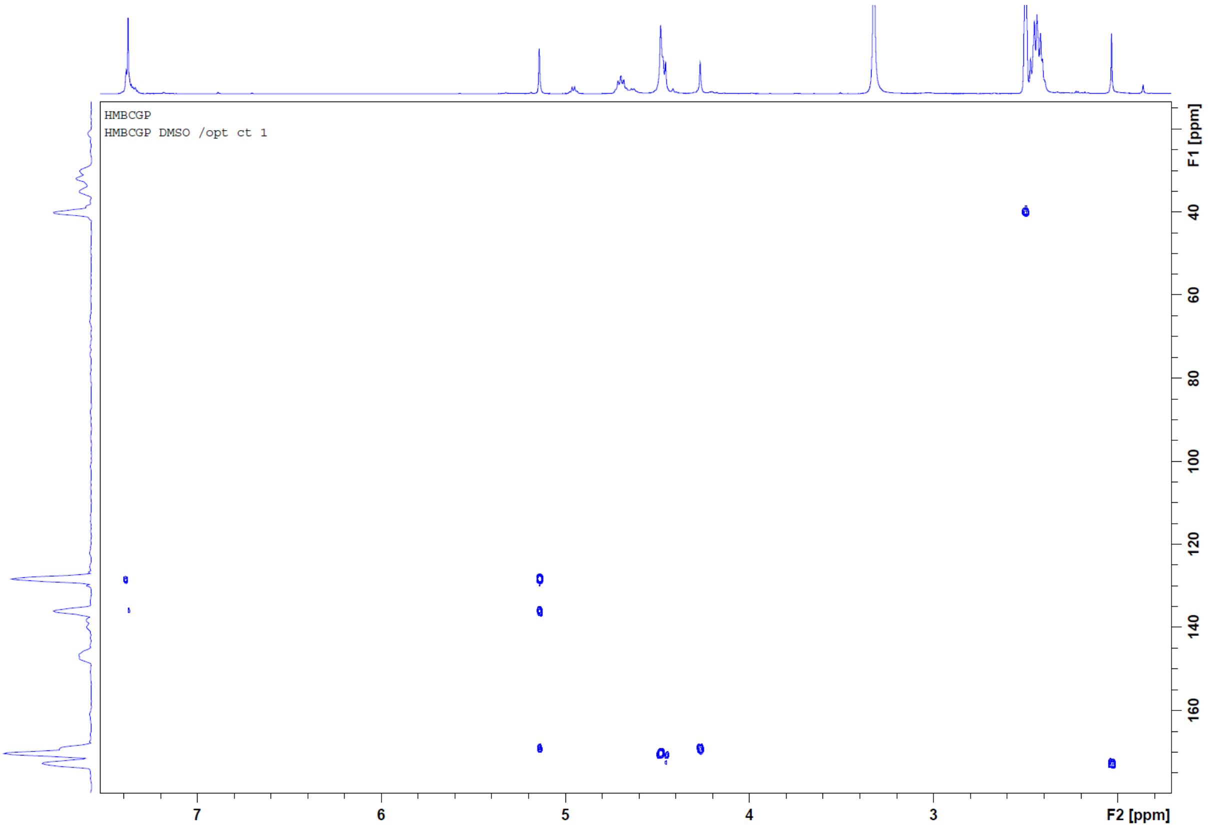


## 6. Variable concentration study

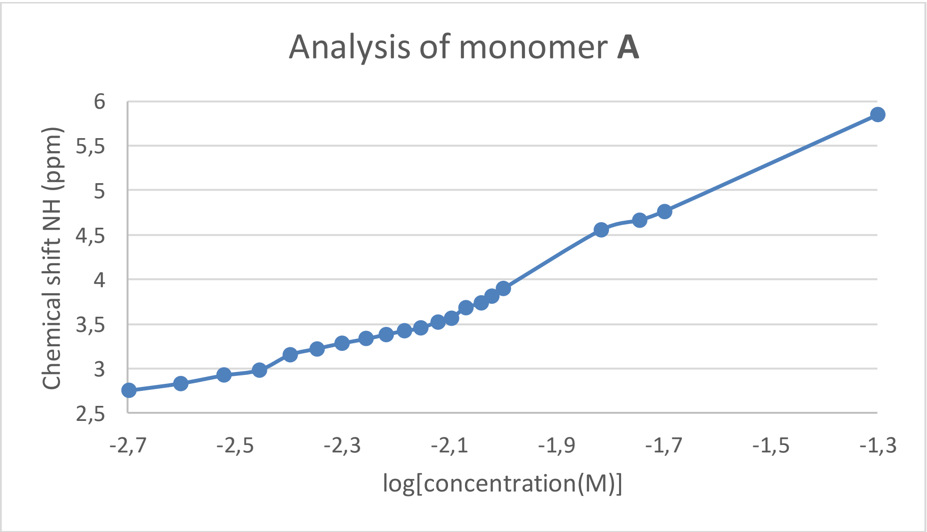


**Figure S1:** NMR chemical shift of the NH proton of the side chain of monomer **A** in $CDCl_3$ at room temperature, as a function of the logarithm of concentration.

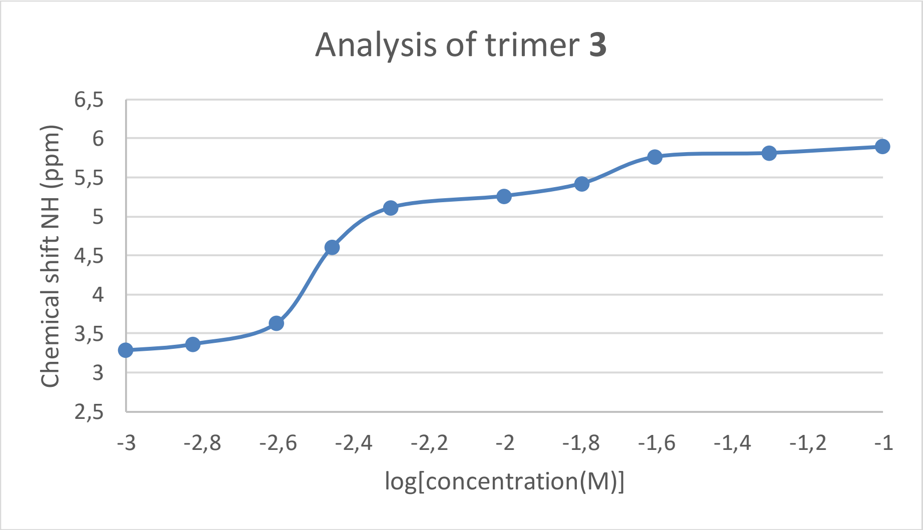


**Figure S2:** NMR chemical shift of the NH proton of the side chain of trimer **3** in $CDCl_3$ at room temperature, as a function of the logarithm of concentration.

## 7. Infrared spectra

### Monomer 1

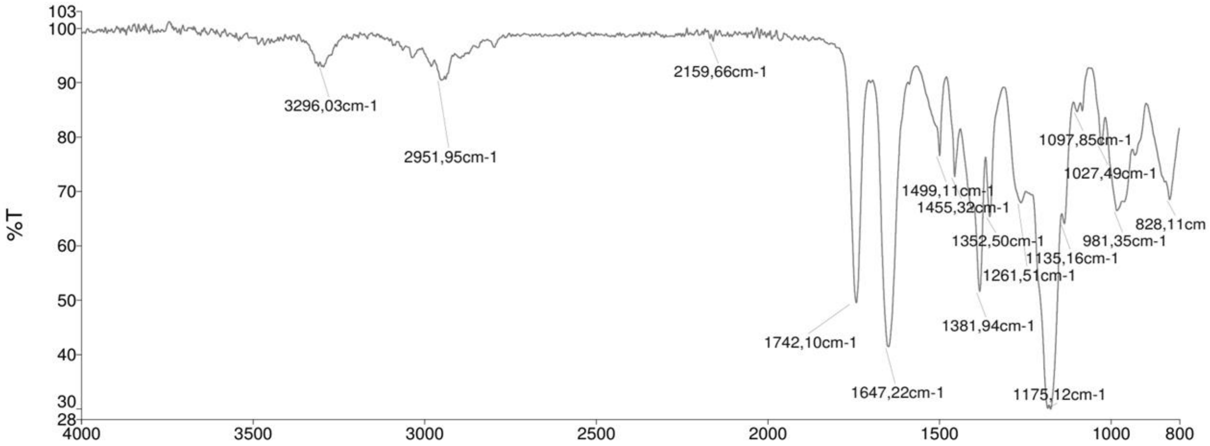

**Dimer 2**

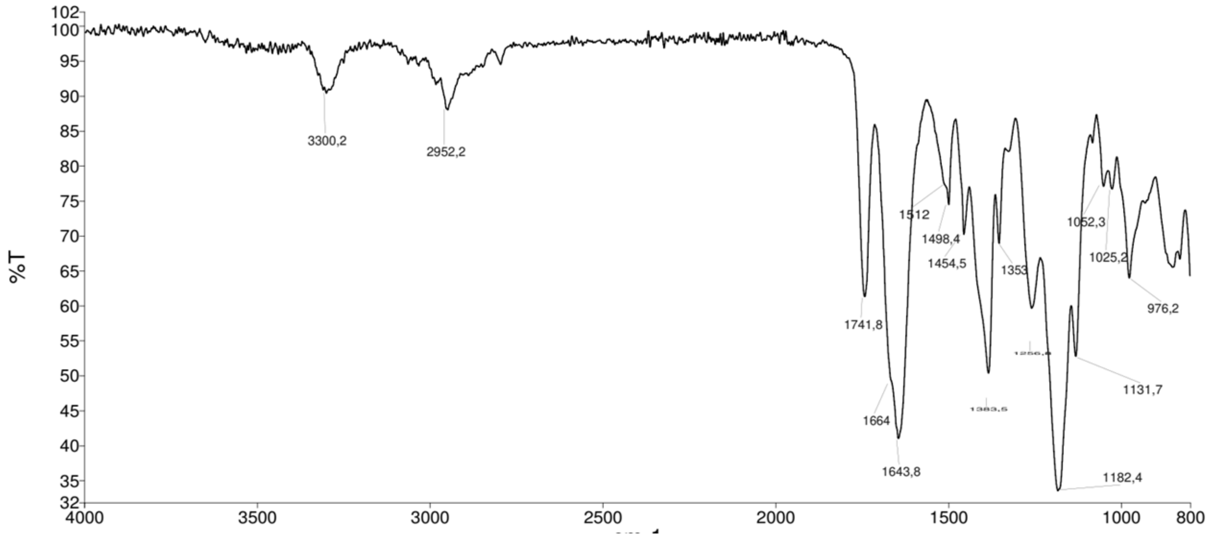


**Trimer 3**

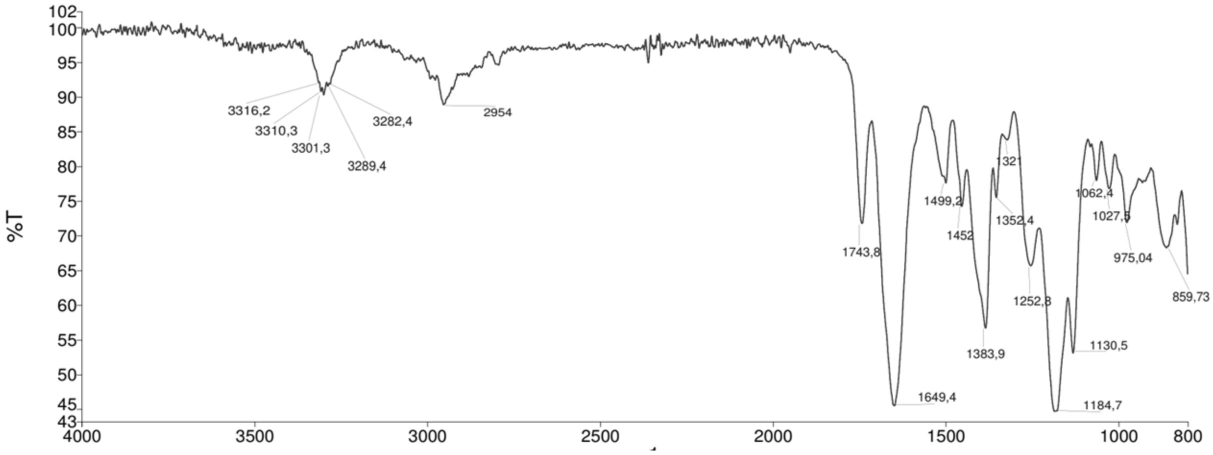


**Tetramer 4**

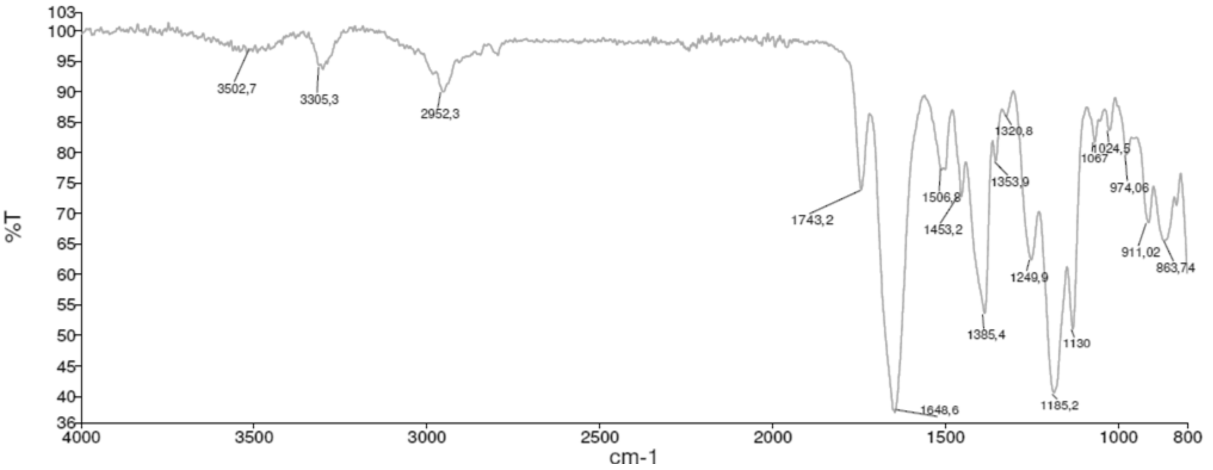

**Pentamer 5**

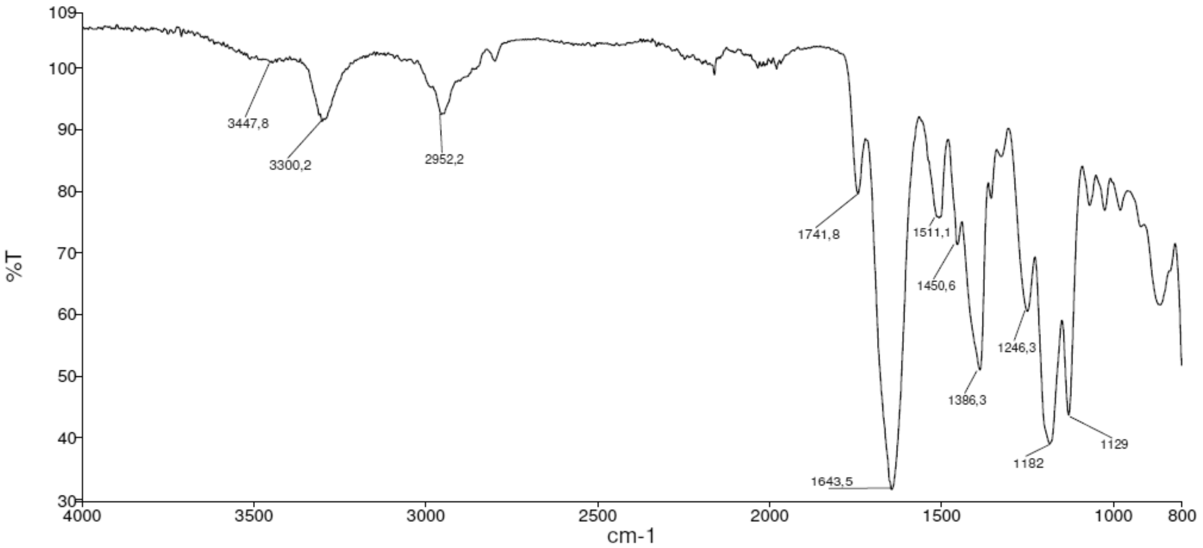


## 8. Computation

All computational studies were performed using Gaussian 16. All structures were optimized using B3LYP functional with the 6-31G(d,p) basis set and using tight convergence criteria (opt = tight) in chloroform (scrf = (solvent = chloroform)). Frequency calculations were performed at the same level of the theory to confirm that the obtained structures were a stationary point (no imaginary frequencies). The model structures were generated by editing the X-ray crystal structure of dimer **2** which exists in a repeating (pp) conformation. The *C*-terminal benzyl ester was switched to a dimethylamide end-cap. For the models featuring alternating conformations (pm), mirror images conformations were generated.

### a) Geometry optimization of monomer model Ac-*N*NMe-NMe$_2$

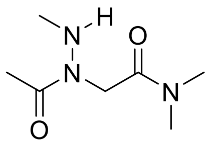


**Table S1.** Dihedral angles, N···O distance and NHO angle measured in the model monomer

| conformation[a] | $\omega$ (°) | $\varphi$ (°) | $\psi$ (°) | $\chi 1$ (°) | d N...O (Å) | NH...O (°) |
|---|---|---|---|---|---|---|
| p | -173.71 | 101.36 | -175.71 | 124.59 | 2.88 | 125.71 |

[a]The conformation is denoted p when the $\varphi$ angle is positive.

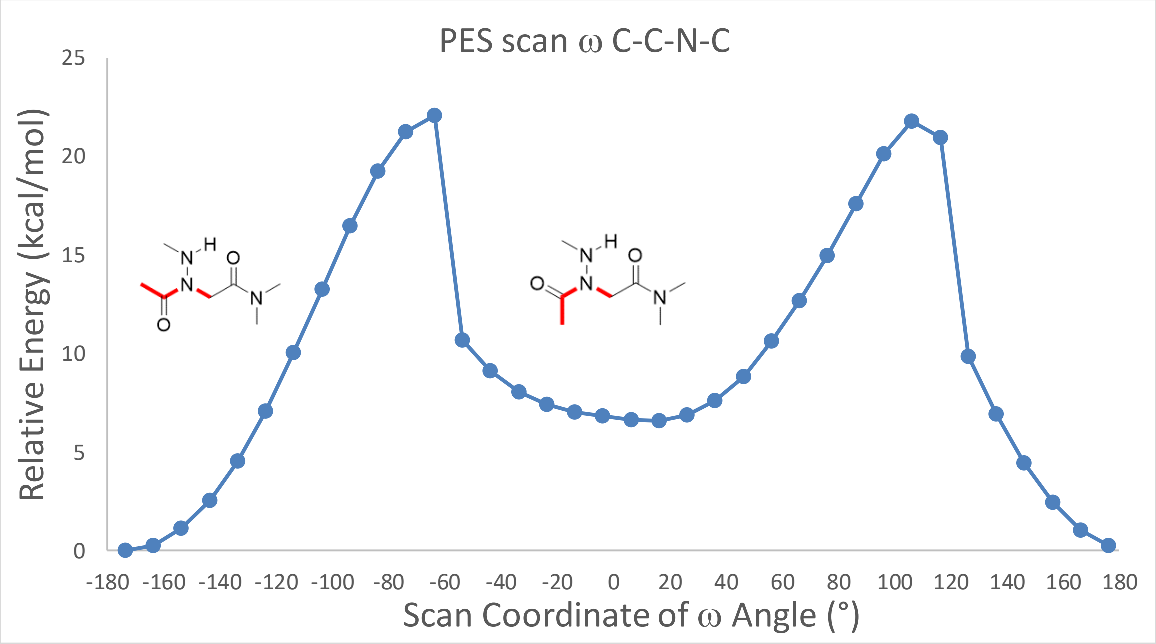


**Figure S3.** Relative energy for relaxed potential energy surface scan about the ω dihedral angle with 35 steps of 10°.

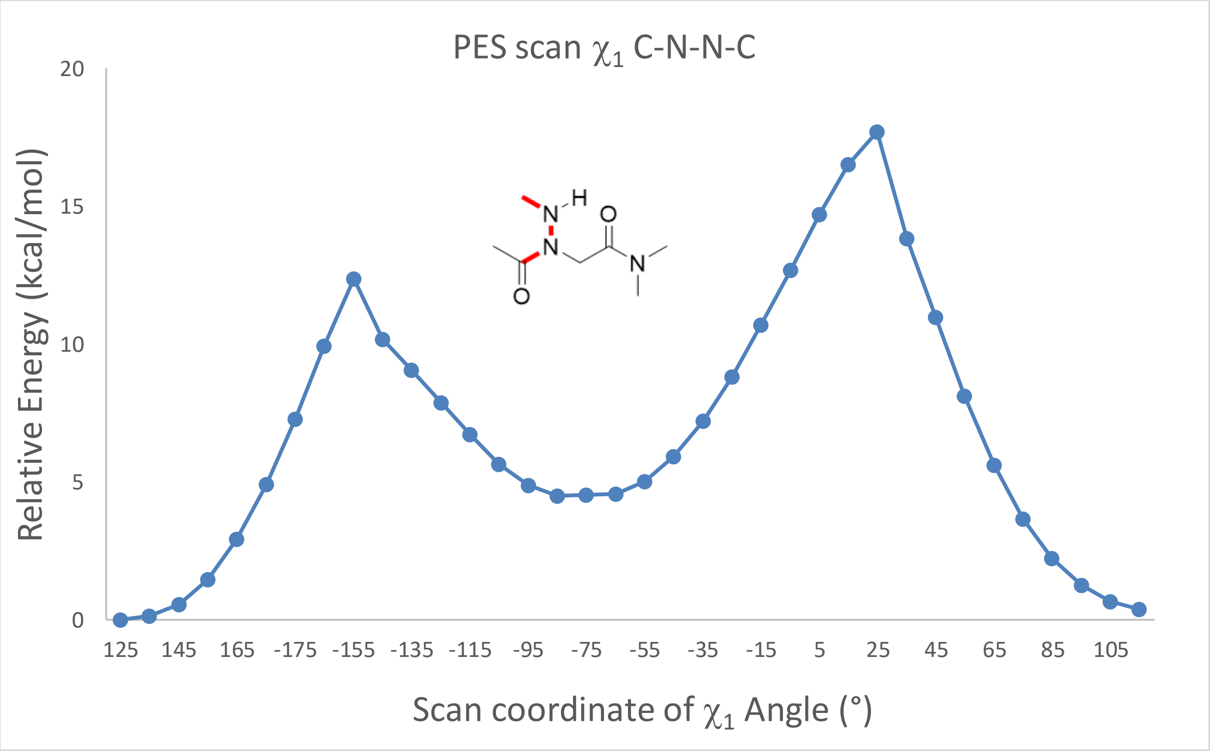


**Figure S4.** Relaxed potential energy scan was about the $\chi^1$ dihedral angle with 35 steps of 10° with freeze coordinate for dihedral angle N-N(-H)-C to avoid nitrogen pyramidal inversion.

### b) Geometry optimization of dimer model Ac-(*N*NMe)$_2$-NMe$_2$

**Table S2.** Dihedral angles, N···O distance and NHO angle measured in the dimer models (pp) and (pm).

| conformation[a,b] | residue | $\omega$ (°) | $\varphi$ (°) | $\psi$ (°) | $\chi 1$ (°) | D N...O | NH...O(°) | relative energy (kcal/mol) |
|---|---|---|---|---|---|---|---|---|
| pp | i | -173.70 | 97.70 | -173.59 | 125.60 | 2.93 | 124.36 | 0 |
| | i+1 | -173.22 | 101.62 | -176.48 | 123.49 | 2.89 | 124.93 | |
| pm | i | -175.06 | 107.94 | 177.71 | -114.7 | 2.91 | 125.60 | 0.65 |
| | i+1 | 175.54 | -104.68 | 176.11 | 112.88 | 2.09 | 126.35 | |

[a]The conformation is denoted p when the $\varphi$ dihedral angle is of positive sign and m when $\varphi$ is negative.

### c) Geometry optimization of hexamer model Ac-(*N*NMe)$_6$-NMe$_2$

**Table S3.** Dihedral angles, N···O distance and NHO angle measured in the hexamer models (p)$_6$ and (pm)$_3$.

| conformation[a,b] | residue | $\omega$ (°) | $\varphi$ (°) | $\psi$ (°) | $\chi 1$ (°) | d N O | NH O(°) | relative energy (kcal/mol) |
|---|---|---|---|---|---|---|---|---|
| (p)$_6$ | i | -173.54 | 97.97 | -173.40 | -111.24 | 2.92 | 124.78 | 0 |
| | i+1 | -173.44 | 100.96 | -173.06 | -113.98 | 2.92 | 124.93 | |
| | i+2 | -173.32 | 101.23 | -174.42 | -113.78 | 2.92 | 124.74 | |
| | i+3 | -172.77 | 100.17 | -173.64 | -113.21 | 2.92 | 124.66 | |
| | i+4 | -172.99 | 98.95 | -173.46 | -112.87 | 2.93 | 123.97 | |
| | i+5 | -172.67 | 101.02 | -176.69 | -112.56 | 2.89 | 124.80 | |
| (pm)$_3$ | I | -175.51 | 111.45 | 177.77 | -116.76 | 2.89 | 126.61 | 2.9 |
| | i+1 | 177.22 | -110.39 | -179.84 | 115.05 | 2.88 | 126.83 | |
| | i+2 | -176.58 | 109.71 | 178.91 | -114.59 | 2.88 | 126.61 | |
| | i+3 | 175.93 | -109.46 | 179.77 | 114.66 | 2.88 | 126.84 | |
| | i+4 | -175.14 | 104.56 | -179.82 | -112.10 | 2.90 | 125.63 | |
| | i+5 | 173.98 | -103.92 | 173.71 | 112.98 | 2.86 | 126.88 | |

[a]The conformation is denoted p when the $\varphi$ dihedral angle is of positive sign and m when $\varphi$ is negative.

## 9. Transmission electron microscopy (TEM)

TEM images were acquired using a JEOL 2100Plus TEM microscope (Tokyo, Japan) operating at 200 kV. Particles contained in a 1 wt % solution of dimer **2** in water were collected onto 400-mesh Cu electron microscopy grid supported with carbon-coated Formvar (Pelanne Instruments, Toulouse, France) by centrifugation at 20.000*g* for 30 min. The grids were individually stained with 2% (w/v) uranyl acetate for 30 s, after which they were rinsed with ultrapure water and immediately dried with absorbent paper.

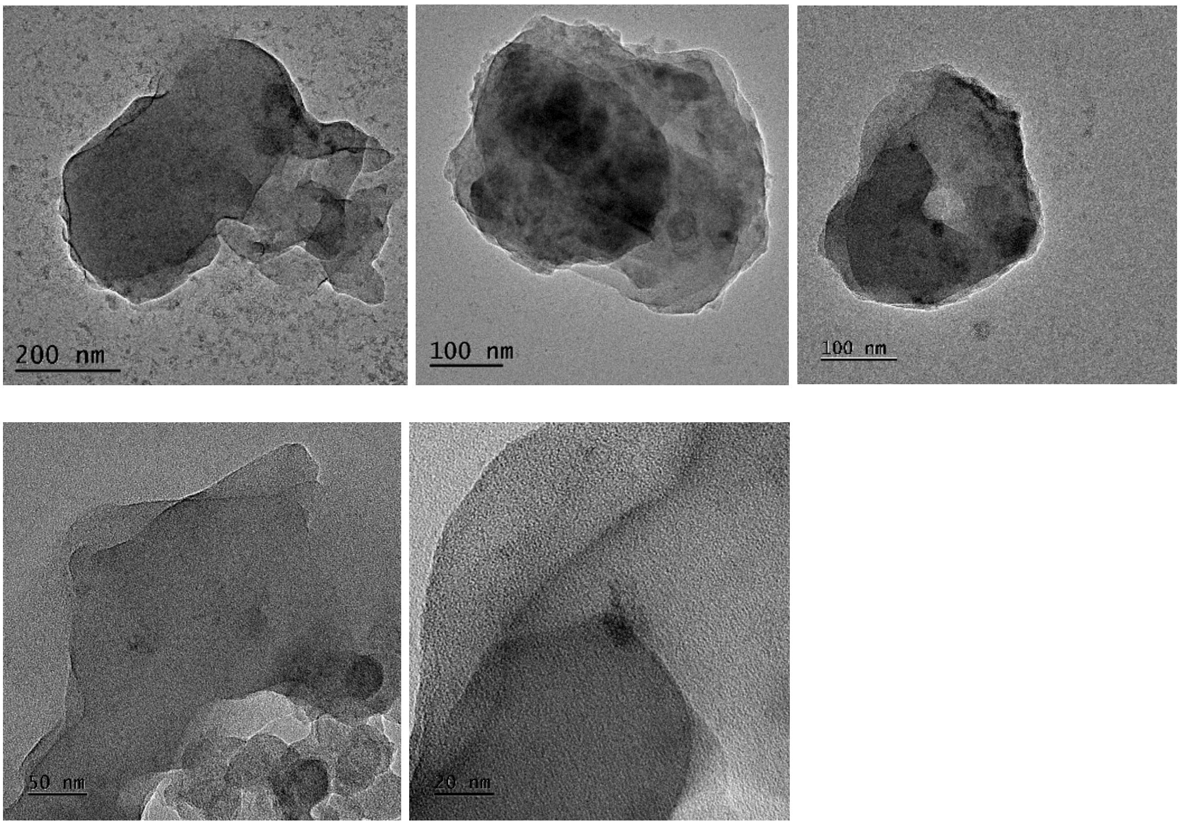

**Figure S5.** TEM images of dimer **2.**
